# Effectiveness of corticosteroids in patients with sepsis or septic shock using the new third international consensus definitions (Sepsis-3): A retrospective observational study

**DOI:** 10.1371/journal.pone.0243149

**Published:** 2020-12-03

**Authors:** Yu-Pu Wu, Julie C. Lauffenburger

**Affiliations:** 1 Department of Epidemiology, Harvard T.H. Chan School of Public Health, Boston, Massachusetts, United States of America; 2 Division of Pharmacoepidemiology and Pharmacoeconomics, Department of Medicine, Brigham and Women’s Hospital and Harvard Medical School, Boston, Massachusetts, United States of America; Vita Salute University of Milan, ITALY

## Abstract

**Background:**

The effects of intravenous corticosteroids in patients with sepsis remain controversial due to mixed results from randomized trials. Moreover, updated definitions of sepsis, Sepsis-3, were proposed in 2016, and findings related to the effects of corticosteroids in patients defined by the Sepsis-3 criteria are scarce.

**Objective:**

To investigate the effectiveness of corticosteroids in patients with sepsis or septic shock using real-world data to complement the findings of randomized controlled trials, and to determine whether the treatment effects differ by sepsis definitions.

**Methods:**

We conducted this study by utilizing a large, multi-center healthcare database, eICU, in which we identified patients with sepsis admitted to 208 intensive care units across the US from 2014 to 2015 based on two different definitions: prior explicit definitions (i.e., based on diagnosis codes) and the Sepsis-3 definitions (i.e., based on SOFA score). The association between intravenous corticosteroids and in-hospital survival up to 50 days in patients with sepsis was retrospectively analyzed. A parametric hazard model with stabilized inverse probability of treatment weight adjustment was used to control for baseline confounders.

**Results:**

Of the 7,158 patients identified based on the explicit definition, 562 (7.9%) received corticosteroids; of the 5,009 patients identified based on the Sepsis-3 definition, 465 (9.3%) received corticosteroids. In the explicit cohort, adjusted in-hospital survival at day 50 was 0.62 in the treated vs 0.57 in the non-treated, with a survival difference of 0.05 (95%CI: -0.11, 0.17). Similar results were seen in the Sepsis-3 cohort (0.58 vs 0.56 in treated and non-treated, respectively), with a 50-day survival difference of 0.02 (95%CI: -0.19, 0.17).

**Conclusions:**

In patients with sepsis or septic shock, intravenous corticosteroids were not associated with a higher in-hospital survival up to 50 days regardless of the sepsis definitions. Further research may be necessary to definitively confirm effectiveness in real-world practice.

## Introduction

Administering corticosteroids to patients suffering from sepsis or septic shock has been controversial for decades [1]. While five meta-analyses of randomized controlled trials (RCTs) suggested that corticosteroid use may reduce mortality for patients with sepsis or septic shock [2–6], four others did not reach the same conclusion [7–10]. In 2018, two large RCTs, ADRENAL [11] and APROCCHSS [12], studying this question revealed differing results: in APROCCHSS, corticosteroid treatment in patients with septic shock significantly reduced 90-day mortality, whereas in ADRENAL, corticosteroids did not reduce 90-day mortality.

One potential reason for these mixed findings may be because prior criteria [13, 14] based on systemic inflammatory response syndrome (SIRS) were too sensitive in clinical settings; many with non-infectious disease also developed SIRS [15]. Therefore, in 2016, to provide more consistent and precise definitions of sepsis, several societies published new consensus definitions for diagnosing sepsis and septic shock (“Sepsis-3”) [16]. The Sepsis-3 definition replaced SIRS with the Sequential Organ Failure Assessment (SOFA) score to better capture patients with a higher rate of organ failure and mortality [17, 18]. As a result, a post hoc analysis of the ADRENAL trial revealed that only 52% of participants met the Sepsis-3 criteria [19], indicating that study populations may differ by the definitions of sepsis.

To our knowledge, no one has yet used real-world data with non-trial populations to examine whether the effectiveness of corticosteroids in patients with sepsis is modified by different sepsis definitions (the older sepsis definitions versus the Sepsis-3 definitions). Therefore, we designed a study utilizing large healthcare data to examine the association between corticosteroids and in-hospital mortality in patients with sepsis or septic shock defined by the prior explicit and sepsis-3 definitions. The objectives were to (1) determine whether effectiveness is modified by different sepsis definitions; and (2) evaluate the effectiveness of corticosteroids in real-world settings as complementary evidence to RCTs.

## Materials and methods

This study was deemed exempt from the Harvard T.H. Chan School of Public Health Institutional Review Board. We followed the extended RECORD checklist recommended for pharmacoepidemiology research (RECORD-PE) [20] for reporting. While we were not specifically replicating a given trial, we followed principles by Hernan and Robins on target trials to design the study [21]. All data were fully anonymized before we accessed them, and all patients who discharged between 2014 and 2015 were accessed.

### Data sources

We utilized a large electronic healthcare database, eICU (https://eicu-crd.mit.edu/), including ≥139,000 unique patients admitted to different Intensive Care Units (ICUs) at 208 hospitals distributed across the US from 2014 to 2015 [22]. The eICU database includes comprehensive demographic and clinical data throughout patient’s hospital stays including, admission and discharge dates to the hospital and the ICU, vital signs, laboratory tests, prescribed and administered medications, and medical diagnoses with past medical history during admissions. These data allow us to study this research question because sepsis-3 definitions [16] require patients’ vital signs and laboratory data to calculate SOFA scores. The eICU search strategies are shown in the S1–S10 Tables.

### Study design and patient population

In order to compare the effectiveness of corticosteroids in patients with sepsis defined by different definitions, we applied two algorithms to identify patients with sepsis. First, we explicitly defined sepsis based on sepsis ICD-9 (995.92; 785.52) or ICD-10 (R65.20; R65.21) diagnoses documented by clinicians during the ICU stay, because a validation study showed these ICD-9 codes had a PPV of 100% for identifying patients with severe sepsis [23]. To our knowledge, no validation study is yet available for the ICD-10 codes; however, these codes largely mirror the ICD-9 codes in eICU. We defined the time of sepsis diagnosis as the onset of sepsis. Because Sepsis-3 was published in 2016, and eICU only included patients admitted to the hospitals from 2014–2015 [22], we assumed that all sepsis-related diagnoses in eICU were based on the old definition [13, 14].

Second, we used explicit sepsis ICD codes combined with a total SOFA score ≥2 prior to the onset of sepsis as a proxy of the Sepsis-3 definition [16]. In this definition, all patients with suspected infection and an acute change in their total SOFA score ≥2 would be identified as having sepsis. However, the lack of complete body fluid cultures in the eICU data affected our ability to accurately define the onset of infection. We therefore used the time of sepsis diagnosis as a surrogate because patients with sepsis must have had an onset of infection prior to the onset of sepsis; the highest total SOFA score was assessed within 48 hours before sepsis onset (S1 Fig). To capture acute changes in total SOFA score, some subcategories were adjusted accordingly for patients who had a medical history of liver, renal, or respiratory failure. We included all patients with total SOFA score ≥2 within that 48-hour assessment window.

The index time was defined as the onset of sepsis (the time of sepsis diagnosis) for the two cohorts (S1 Fig). All patients were followed until discharge from the hospital (dead or alive). For all definitions, we excluded patients who: were <18 years, received any intravenous corticosteroids or etomidate before the onset of sepsis, had a history of long-term corticosteroids or other immunosuppressive drug use, or with HIV/AIDS, autoimmune disease, receiving cancer therapy, hematological or metastatic cancer, or organ transplantation. These criteria mimic enrollment criteria for the prior RCTs [11, 12] and are precautions or contraindications to intravenous corticosteroids or indications of previous receipt [24, 25].

### Exposures

In order to mimic the corticosteroid treatments used in RCTs [4, 11, 26, 27], patients with sepsis who met the following two exposure criteria were classified as treated: (1) received any intravenous corticosteroids ≥24 hours continuously within 72 hours after the onset of sepsis and (2) had a treatment duration of ≤11 days. Those patients who did not meet the exposure criteria in the same exposure window were identified as controls. While an active comparator would be ideal, there is no real-world active comparator, so to mitigate this, we did sensitivity analyses comparing patients with different steroid doses. We handled immortal time bias [28] by excluding all patients who died within 96 hours after onset of sepsis (S1 Fig) and conducted several sensitivity analyses varying this exclusion.

### Outcomes

The outcome of interest was in-hospital survival (which is the survival probability, and we used the term “survival” in the whole manuscript) up to 50 days beginning with the index time. Those patients with longer lengths of hospitalization had their follow-up administratively censored at day 50 after index. We chose 50 days of follow-up not only because the mean length of hospital stay among patients with sepsis from the RCTs [11, 26, 29–31] ranged from 21 to 43 days but also because this window covered the follow-up time for almost the entire population and reduced any potential bias by outliers. Patients discharged alive were censored at the discharge date. Because the data are directly derived from inpatient medical records, in-hospital mortality and survival can be considered accurate.

### Confounding variables

We measured covariates thought to be associated with intravenous corticosteroid treatment or in-hospital mortality based on clinical judgement and prior literature as potential confounders [18, 32–35]. All variables were evaluated before the index time. These variables included age, sex, race, primary reason for hospitalization (surgery vs non-surgery), total and six subcategories of SOFA score, mechanical ventilation use, comorbidities (i.e. diabetes mellitus, arrhythmia, asthma, chronic obstructive pulmonary disease (COPD), coronary artery diseases, cancer history, cerebrovascular accident, gastrointestinal bleeding, heart failure, renal failure, respiratory failure), lab data (white blood cell count, blood glucose, serum lactate, liver aminotransferases, serum potassium, serum sodium, hemoglobin, percentage of band neutrophils, serum ammonia, and Troponin-I), and facility-level factors such as geographic region and number of beds. Comorbidities were evaluated based on past medical history. Lab data were extracted within 1-day prior to index. If the same labs were measured multiple times within this 1-day window, we chose the value representing the worst clinical condition. Individuals aged >89 were masked due to HIPPA regulation [22], so we classified all patients aged >89 as age 90. All laboratory values were dichotomized into clinically normal vs abnormal; untested laboratory data (S11 Table) were assumed to be normal.

### Statistical analysis

Our primary objective was to examine the association between corticosteroid treatment and in-hospital survival up to 50 days in patients with sepsis defined by different algorithms. To control for confounding, we developed logistic models to generate stabilized inverse probability treatment weights (IPTW) and further apply the weights to the final outcome model. First, for the numerator of the weight, we used a saturated logistic model including only an intercept to estimate the probability of getting observed treatment. Second, for the denominator of the weights, we developed a logistic model to estimate the probability of getting observed treatment given all the confounding variables. After obtaining the stabilized weights, we further created a person-day IPTW weighted hazards outcome model in which we included time-varying intercept and product terms (time*treatment and time^2^*treatment) to allow the hazard ratio to vary over time [36–38]. We used absolute standardized differences to examine the balance of baseline characteristics between the treated and non-treated before and after IPTW weighting. Balance was achieved if absolute standardized differences were ≤0.1 for all the covariates [39, 40].

We evaluated the crude and IPTW weighted in-hospital survival up to 50 days and survival differences between treatment groups using 95% confidence intervals (95%CI). In specific, we calculated 95%CI for survival differences using bootstrapping with 500 iterations using random sampling with replacement [36, 41].

Subgroup analyses were conducted by sepsis severity (sepsis versus septic shock). In the first cohort, patients were stratified by having septic shock ICD codes or not (ICD-9: 785.52; ICD-10: R65.21), whereas in the second cohort, septic shock was defined as patients who had lactate ≥2 mmol/L and also received vasopressors [16]. We conducted the following sensitivity analyses: (1) assessing the total SOFA score over shorter time windows ranging from 3 hours to 1 day prior to sepsis onset; (2) using different windows to define corticosteroid treatment (i.e., 1-day and 5-day, adjusting accordingly for immortal time bias); (3) comparing the treated to those receiving any dose of corticosteroids; (4) including daily hydrocortisone-equivalent corticosteroid doses within 200mg and 400mg as inclusion criterion to better mimic RCTs [4, 11]; (5) excluding control patients who received any intravenous corticosteroids after sepsis onset to reduce the effects of short-term or very long-term corticosteroids; (6) excluding patients with asthma or COPD; (7) excluding hospitals that never prescribed corticosteroids to patients with sepsis to reduce potential facility-level confounding; (8) applying the g-formula [36] instead of IPTW with same controlled covariates to overcome any model misspecification; (9) excluding patients had >1 hospitalizations due to sepsis in the same calendar year; (10) assessing the in-hospital survival up to 90 days; (11) excluding patients without ventilation support; (12) excluding patients admitted for surgery; (13) selecting patients who received hydrocortisone as treated; (14) assuming all the untested values as abnormal; and (15) excluding those laboratory values with high percentage of untested patients including serum lactate, ALT, AST, % of band neutrophils, serum ammonia, and troponin-I from the analysis.

To avoid model over-fitting for some subgroup or sensitivity analyses in which the sample size of treated patients was smaller than 10*number of adjusted covariates, we used a stepwise model selection with significance levels of 0.1 and 0.2 for model entry, and inclusion, respectively; however, we forced total SOFA score to be included because of its hypothesized importance for confounding control. The final model that yielded the minimum AIC was chosen.

We used SAS (Version 9.4, Cary, NC) for all analyses.

## Results

**Fig 1:**
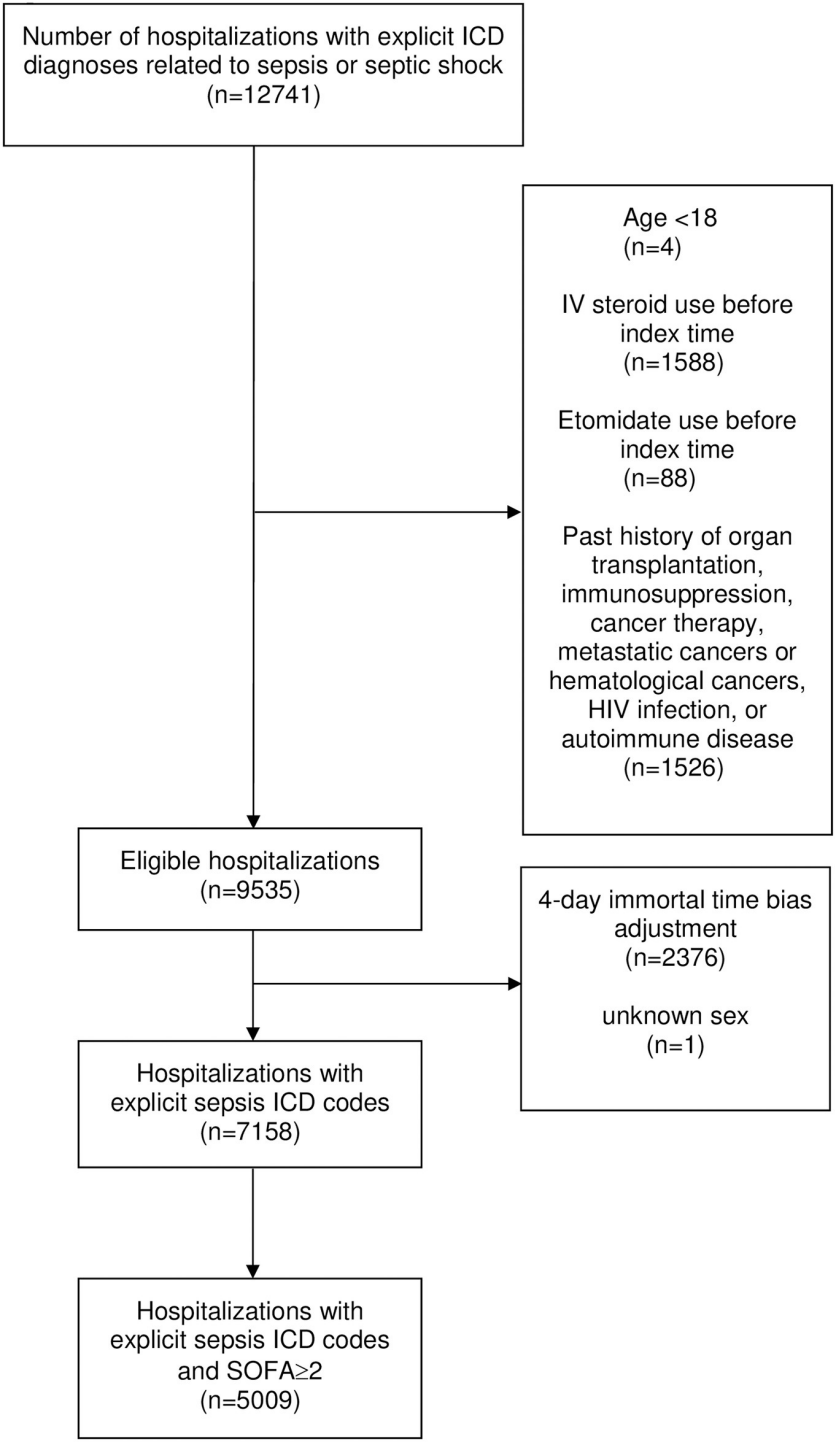
Cohort selection.

Using explicit sepsis diagnosis codes, we identified 12,741 hospitalizations from the eICU data. After applying criteria, 7,118 hospitalizations were identified, of which 562 (7.8%) were classified as treated. After further applying total SOFA≥2 as a proxy of Sepsis-3, the second study cohort included 5,009 hospitalizations of which 465 (9.2%) were classified as treated (Fig 1).

Compared to controls, treated patients in the first cohort had a higher total SOFA score[mean (SD), 4.6 (3.0) vs. 3.4 (2.9)], more ventilation support (47.2% vs 26.4%), and tended to be admitted to larger facilities at baseline than controls (Table 1). In general, patients who received corticosteroids had more comorbidities and abnormal lab tests. In the second cohort defined by the sepsis-3 definition, comorbidities and abnormal lab data had similar distributions as in the first cohort (Table 2).

**Table 1:** Baseline characteristics of the explicit cohort.

Characteristic	Before IPTW			After IPTW		
	Control (n = 6596)	Treated (n = 562)	Absolute Standard Difference	Control (n = 6596)	Treated (n = 562)	Absolute Standard Difference
**Age, year, mean (SD)**	66.39 (16.24)	65.77 (15.58)	0.00	66.34 (16.24)	66.46 (15.49)	0.00
**Total SOFA score, mean (SD)**^a^	3.40 (2.86)	4.57 (3.03)	0.13	3.49 (2.91)	3.49 (2.78)	0.00
**Female, No. (%)**	3139 (47.59)	289 (51.42)	0.08	3157.53 (47.87)	264.60 (46.70)	0.02
**Ventilation Support, No. (%)**	1741 (26.39)	265 (47.15)	0.44	1848.43 (28.02)	149.71 (26.42)	0.04
**Admitted for Surgery, No. (%)**	184 (2.79)	9 (1.60)	0.08	177.77 (2.69)	13.75 (2.43)	0.02
**Race, No. (%)**						
**White**	5023 (76.15)	432 (76.87)	0.02	5025.97 (76.19)	413.46 (72.97)	0.07
**Non-White**	1573 (23.85)	130 (23.13)	0.02	1570.65 (23.81)	153.12 (27.03)	0.07
**Comorbidity, No. (%)**						
**Diabetes**	2366 (35.87)	190 (33.81)	0.04	2354.23 (35.69)	193.86 (34.22)	0.03
**Arrhythmia**	934 (14.16)	83 (14.77)	0.02	937.77 (14.22)	75.35 (13.30)	0.03
**Asthma or COPD**	1356 (20.56)	134 (23.84)	0.08	1376.24 (20.86)	130.77 (23.08)	0.05
**Coronary Artery Diseases**	1009 (15.30)	91 (16.19)	0.02	1014.69 (15.38)	90.47 (15.97)	0.02
**Cancer history**	688 (10.43)	49 (8.72)	0.06	681.11 (10.33)	70.18 (12.39)	0.06
**Cerebrovascular Accident**	8709(13.33)	68 (12.10)	0.04	872.03 (13.22)	79.99 (14.12)	0.03
**Gastrointestinal Bleeding**	177 (2.68)	16 (2.85)	0.01	178.43 (2.70)	13.89 (2.45)	0.02
**Heart Failure**	1147 (17.39)	104 (18.51)	0.03	1154.53 (17.50)	114.71 (20.25)	0.07
**Renal Failure**	922 (13.98)	97 (17.26)	0.09	938.29 (14.22)	81.29 (14.35)	0.00
**Respiratory Failure**	203 (3.08)	18 (3.20)	0.01	203.47 (3.08)	18.23 (3.22)	0.01
**Lab Data, No. (%)**						
**Elevated WBC Count**^b^	4636 (70.29)	380 (67.62)	0.06	4622.76 (70.08)	400.05 (70.61)	0.01
**Hyperglycemia**^c^	3697 (56.05)	285 (50.71)	0.11	3669.70 (55.63)	310.41 (54.79)	0.02
**Elevated Serum Lactate**^d^	3621 (54.90)	324 (57.65)	0.06	3634.25 (55.09)	302.77 (53.44)	0.03
**Abnormal ALT**^e^	1075 (16.30)	113 (20.11)	0.10	1093.28 (16.57)	86.32 (15.23)	0.04
**Abnormal AST**^f^	1654 (25.08)	169 (30.07)	0.11	1677.73 (25.43)	128.54 (22.69)	0.06
**Abnormal Serum Potassium**^g^	1798 (27.26)	154 (27.40)	0.00	1796.39 (27.23)	145.49 (25.68)	0.04
**Abnormal Serum Sodium**^h^	2233 (33.85)	174 (30.96)	0.06	2216.52 (33.60)	183.16 (32.33)	0.03
**Abnormal Hemoglobin**^i^	4539 (68.81)	369 (65.66)	0.07	4521.47 (68.54)	385.41 (68.02)	0.01
**% of Band Neutrophils**^j^	656 (9.95)	79 (14.06)	0.13	677.29 (10.27)	55.13 (9.73)	0.02
**Abnormal Serum Ammonia**^k^	170 (2.58)	22 (3.91)	0.08	176.39 (2.67)	12.92 (2.28)	0.03
**Abnormal Troponin-I**^l^	271 (4.11)	29 (5.16)	0.05	276.28 (4.19)	26.23 (4.63)	0.02
**Facility-level Factors, No. (%)**						
**Number of Beds ≥ 500**	1825 (27.67)	224 (39.86)	0.26	1891.00 (28.67)	162.53 (28.51)	0.00
**Region, No. (%)**						
**South**	1457 (22.09)	111 (19.75)	0.06	1444.28 (21.89)	117.78 (20.79)	0.03
**Northeast**	600 (9.10)	69 (12.28)	0.10	617.42 (9.36)	56.75 (10.02)	0.02
**Other**	4539 (68.81)	382 (67.97)	0.02	4534.93 (68.75)	392.05 (69.20)	0.01

Abbreviation: IPTW, inverse probability treatment weight; WBC, White Blood Cell; ALT, Alanine Aminotransferase; AST, Aspartate Aminotransferase.

^a^Baseline characteristics of the six subcategories of SOFA score were shown in the S12 Table;

^b^WBC count >9,600 cells/mcL;

^c^serum glucose >126 mg/dL;

^d^Serum Lactate ≥2 mmol/L;

^e^ALT>55 U/L;

^f^AST>48 U/L;

^g^Serum Potassium <3.6 mmol/L or >5.2 mmol/L;

^h^Serum Sodium <135 mEq/L or >145 mEq/L;

^i^Hemoglobin <12 for female or <13.5 for male;

^j^% of Band Neutrophils >10;

^k^Serum ammonia >45μ/dL;

^l^Troponin-I >0.4 ng/ml.

**Table 2:** Baseline characteristics of the Sepsis-3 cohort.

Characteristic	Before IPTW			After IPTW		
	Control (n = 4544)	Treated (n = 465)	Absolute Standard Difference	Control (n = 4544)	Treated (n = 465)	Absolute Standard Difference
**Age, year, mean (SD)**	66.48 (16.00)	65.35 (15.64)	0.00	66.37 (16.04)	66.42 (15.64)	0.00
**Total SOFA score, mean (SD)**^a^	4.74 (2.45)	5.44 (2.57)	0.11	4.81 (2.47)	4.75 (2.41)	0.01
**Female, No. (%)**	2069 (45.53)	236 (50.75)	0.10	2090.19 (46.00)	208.96 (44.36)	0.03
**Ventilation Support, No. (%)**	1305 (28.72)	239 (51.40)	0.48	1400.48 (30.82)	138.56 (29.42)	0.03
**Admitted for Surgery, No. (%)**	138 (3.04)	8 (1.72)	0.09	132.38 (2.91)	11.54 (2.45)	0.03
**Race, No. (%)**						
**White**	3445 (75.81)	357 (76.77)	0.02	3448.46 (75.89)	340.14 (72.21)	0.08
**Non-White**	1099 (24.19)	108 (23.23)	0.02	1095.65 (24.11)	130.89 (27.79)	0.08
**Comorbidity, No. (%)**						
**Diabetes**	1616 (35.56)	159 (34.19)	0.03	1609.14 (35.14)	155.50 (33.01)	0.05
**Arrhythmia**	619 (13.62)	70 (15.05)	0.04	625.87 (13.77)	62.76 (13.32)	0.01
**Asthma or COPD**	856 (18.84)	95 (20.43)	0.04	864.57 (19.03)	95.45 (20.26)	0.03
**Coronary Artery Diseases**	702 (15.45)	74 (15.91)	0.01	704.77 (15.51)	75.44 (16.02)	0.01
**Cancer history**	443 (9.75)	38 (8.17)	0.06	438.14 (9.64)	58.95 (12.51)	0.09
**Cerebrovascular Accident**	587 (12.92)	56 (12.04)	0.04	582.70 (12.82)	65.14 (13.83)	0.03
**Gastrointestinal Bleeding**	119 (2.62)	13 (2.80)	0.01	120.25 (2.65)	12.19 (2.59)	0.00
**Heart Failure**	762 (16.77)	84 (18.06)	0.03	769.05 (16.92)	90.76 (19.27)	0.06
**Renal Failure**	529 (11.64)	83 (17.85)	0.18	555.76 (12.23)	62.35 (13.24)	0.03
**Respiratory Failure**	127 (2.79)	13 (2.80)	0.00	126.65 (2.79)	12.78 (2.71)	0.00
**Lab Data, No. (%)**						
**Elevated WBC Count**^b^	3220 (70.86)	323 (69.46)	0.03	3214.79 (70.75)	341.37 (72.47)	0.04
**Hyperglycemia**^c^	2581 (56.80)	241 (51.83)	0.10	2560.38 (56.35)	259.27 (55.04)	0.03
**Elevated Serum Lactate**^d^	2602 (57.26)	279 (60.00)	0.06	2612.29 (57.49)	259.83 (55.16)	0.05
**Abnormal ALT**^e^	876 (19.28)	107 (23.01)	0.09	890.74 (19.60)	84.13 (17.86)	0.04
**Abnormal AST**^f^	1376 (30.28)	158 (33.98)	0.08	1389.86 (30.59)	126.72 (26.90)	0.08
**Abnormal Serum Potassium**^g^	1307 (28.76)	133 (28.60)	0.00	1304.00 (28.70)	121.40 (25.77)	0.07
**Abnormal Serum Sodium**^h^	1612 (35.48)	152 (32.69)	0.06	1598.71 (35.18)	158.49 (33.65)	0.03
**Abnormal Hemoglobin**^i^	3289 (72.38)	320 (68.82)	0.08	3273.09 (72.03)	342.69 (72.75)	0.02
**% of Band Neutrophils**^j^	515 (11.33)	72 (15.48)	0.12	531.97 (11.71)	52.03 (11.05)	0.02
**Abnormal Serum Ammonia**^k^	151 (3.32)	20 (4.30)	0.05	154.39 (3.40)	12.38 (2.63)	0.05
**Abnormal Troponin-I**^l^	225 (4.95)	26 (5.59)	0.03	227.26 (5.00)	23.78 (5.05)	0.00
**Facility-level Factors, No. (%)**						
**Number of Beds ≥ 500**	1442 (31.73)	198 (42.58)	0.23	1489.31 (32.77)	149.71 (31.78)	0.02
**Region, No. (%)**						
**South**	1125 (24.76)	97 (20.86)	0.09	1107.57 (24.37)	110.04 (23.36)	0.02
**Northeast**	473 (10.41)	63 (13.55)	0.10	486.64 (10.71)	53.63 (11.39)	0.02
**Other**	2946 (64.83)	305 (65.59)	0.02	2949.9 (64.92)	307.36 (65.25)	0.01

Abbreviation: IPTW, inverse probability treatment weight; WBC, White Blood Cell; ALT, Alanine Aminotransferase; AST, Aspartate Aminotransferase.

^a^Baseline characteristics of the six subcategories of SOFA score were shown in the S13 Table;

^b^WBC count >9,600 cells/mcL;

^c^serum glucose >126 mg/dL;

^d^Serum Lactate ≥2 mmol/L;

^e^ALT>55 U/L;

^f^AST>48 U/L;

^g^Serum Potassium <3.6 mmol/L or >5.2 mmol/L;

^h^Serum Sodium <135 mEq/L or >145 mEq/L;

^i^Hemoglobin <12 for female or <13.5 for male;

^j^% of Band Neutrophils >10;

^k^Serum ammonia >45μ/dL;

^l^Troponin-I >0.4 ng/ml.

The mean, min, and max values of the stabilized weights were 1.00, 0.13, and 4.66, respectively. After IPTW adjustment, balance was achieved for all baseline characteristics in the two study cohorts (i.e. absolute standardized differences ≤0.1).

### 50-day in-hospital survival and survival difference between treatment groups

Median follow-up for both cohorts were 9 days; the 50-day follow-up covered >95% of both cohorts (S2 Fig). Before IPTW adjustment, the treatment group had a higher in-hospital mortality rate: (97 vs 794 deaths; 13.2 vs 10.6 deaths/1000 person-days). In the second study cohort, the treatment group also had a higher in-hospital mortality rate: (89 vs 611 deaths; 14.3 vs 11.3 deaths/1000 person-days).

**Fig 2:**
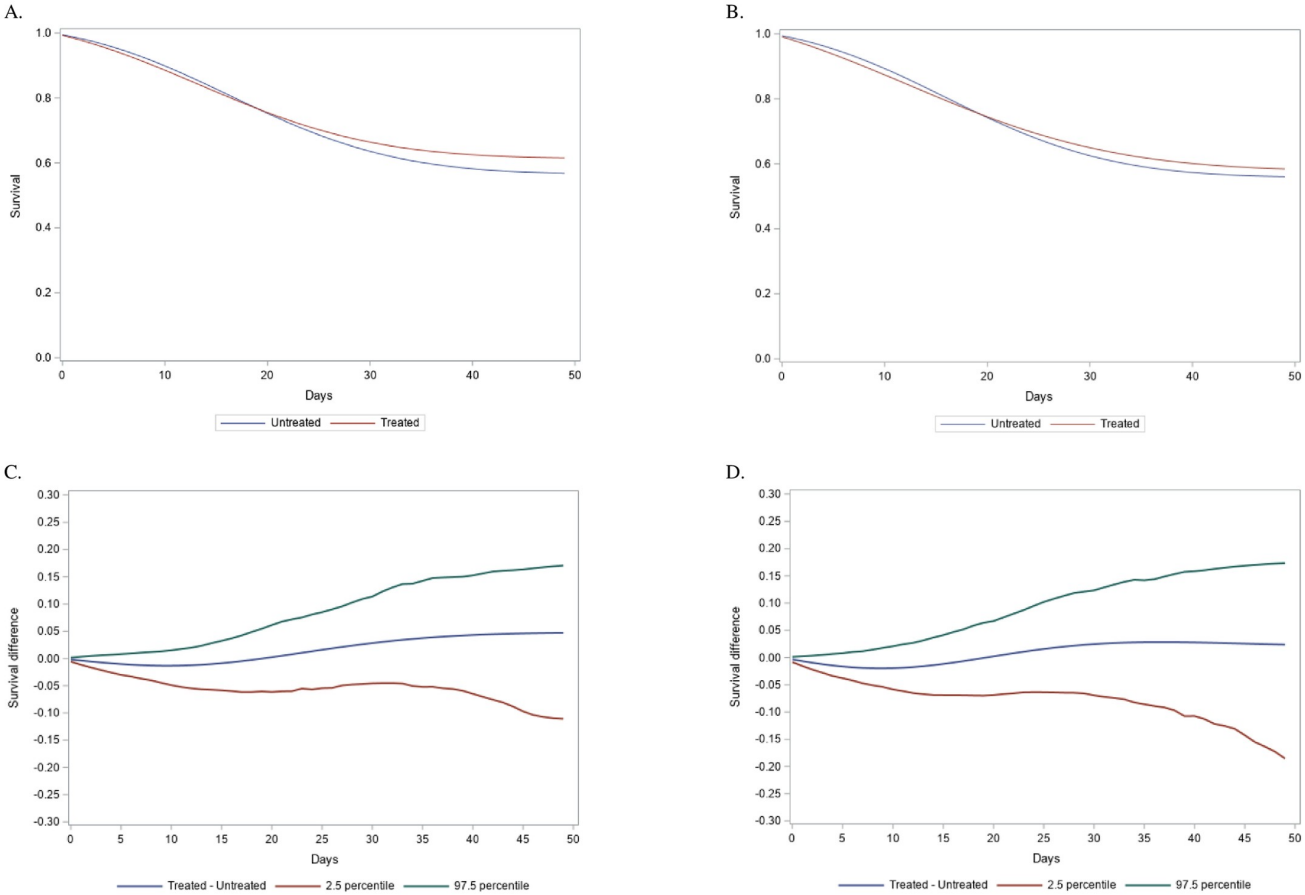
In-hospital survival and survival difference between treated and non-treated after inverse probability treatment weights. A. In-hospital Survival up to 50 days for the Explicit Cohort; B. In-hospital Survival up to 50 days for the Sepsis-3 Cohort; C. In-hospital Survival Difference with 95% CI for the Explicit Cohort; D. In-hospital Survival Difference with 95% CI for the Sepsis-3 Cohort.

Table 3 shows the crude and IPTW adjusted 50-day in-hospital survival and survival difference between treated and non-treated patients. Fig 2 provides the survival curves and the survival difference between two treatment groups. In the first cohort, adjusted in-hospital 50-day survival in the treated was 0.62 vs 0.57 in the non-treated, and the in-hospital survival difference at day 50 was 0.05 (95%CI: -0.11, 0.17). In-hospital survival did not significantly differ between the groups throughout the entire follow-up period. Treated patients had slightly lower survival compared to controls in the first 19 days, with a maximum in-hospital survival difference -0.01 (95%CI: -0.05, 0.01) at day 10. The in-hospital survival difference became positive beginning on day 20 and diverged afterward. Subgroup analyses in which individuals were stratified by their having septic shock diagnosis codes showed similar results (Table 3), but the survival curves did not cross (S3 Fig). All results from the sensitivity analyses agreed with the primary analysis (S14 Table and S4 Fig).

**Table 3:** In-hospital survival and survival difference in the two study cohorts.

**Explicit**			
	**All Patients**	**Only Septic Shock**^a^	**Only Sepsis**
**Before IPTW adjustment**			
**In-hospital 50-Day Survival among Controls**	0.57 (n = 6596)	0.56 (n = 2570)	0.57 (n = 4026)
**In-hospital 50-Day Survival among Treated**	0.56 (n = 562)	0.53 (n = 276)	0.57 (n = 286)
**In-hospital 50-Day Survival Difference with 95%CI**	-0.01 (-0.16, 0.10)	-0.03 (-0.19, 0.13)	0.00 (-0.28, 0.21)
**After IPTW adjustment**			
**In-hospital 50-Day Survival among Controls**	0.57 (n = 6596)	0.56 (n = 2570)	0.57 (n = 4026)
**In-hospital 50-Day Survival among Treated**	0.62 (n = 562)	0.51 (n = 276)	0.71 (n = 286)
**In-hospital 50-Day Survival Difference with 95%CI**	0.05 (-0.11, 0.17)	-0.05* (-0.25, 0.14)	0.14* (-0.13, 0.31)
**Sepsis-3**			
	**All Patients**	**Only Septic Shock**^b^	**Only Sepsis**
**Before IPTW adjustment**			
**In-hospital 50-Day Survival among Controls**	0.56 (n = 4544)	0.54 (n = 1082)	0.57 (n = 3462)
**In-hospital 50-Day Survival among Treated**	0.53 (n = 465)	0.55 (n = 188)	0.52 (n = 277)
**In-hospital 50-Day Survival Difference with 95%CI**	-0.03 (-0.17, 0.10)	0.01 (-0.21, 0.20)	-0.05 (-0.34, 0.14)
**After IPTW adjustment**			
**In-hospital 50-Day Survival among Controls**	0.56 (n = 4544)	0.53 (n = 1082)	0.57 (n = 3462)
**In-hospital 50-Day Survival among Treated**	0.58 (n = 465)	0.53 (n = 188)	0.59 (n = 277)
**In-hospital 50-Day Survival Difference with 95%CI**	0.02 (-0.19, 0.17)	0.00* (-0.29, 0.23)	0.02* (-0.26, 0.22)

Abbreviations: IPTW, inverse probability treatment weight; CI, confidence interval.

^a^ICD-9 code: 785.52 or ICD-10 code: R65.21;

^b^Serum lactate ≥2 and received vasopressors;

*Stepwise model selection was used.

In the second cohort where we further applied total SOFA≥2, the adjusted in-hospital 50-day survival results were similar to the first cohort (i.e. in-hospital 50-day survival difference, 0.02; 95%CI: -0.19, 0.17) (Table 3). Fig 2 shows that survival for treated patients was lower than non-treated patients from day 0 to day 19 and the maximum negative in-hospital survival difference was -0.02 (95%CI: -0.05, 0.02) at day 9. Survival curves for the two groups intersected at day 20 and then gradually diverged but with no significant survival differences. Subgroup analyses exploring whether a new septic shock definition produced different survival benefits were still non-significant (S5 Fig). All sensitivity analyses revealed consistent results with the primary approach (S15 Table and S6 Fig).

## Discussion

In this retrospective study of real-world patients across 208 ICUs in the US, receiving ≥24 hours and ≤11 days of intravenous corticosteroid treatment was not associated with significant difference in in-hospital survival compared with patients who did not receive the defined treatment. These findings were observed regardless of whether the older explicit definition of sepsis was used or whether the newer Sepsis-3 definitions were applied.

The efficacy of intravenous corticosteroid treatment in patients with severe sepsis or septic shock has shown no difference in in-hospital mortality in some RCTs [26, 29, 31, 42–48]. A Cochrane systematic review [49] also revealed that the unpublished in-hospital mortality of the ADRENAL trial was not significantly lower in the corticosteroid group (Risk Ratio, 0.95; 95%CI: 0.86, 1.06). Though non-significant, 7 other RCTs even suggested that treated patients had higher in-hospital mortality [26, 31, 44–48]. These studies agreed with our findings that intravenous corticosteroids were not associated with higher in-hospital survival for patients with sepsis or septic shock.

Another large RCT, APROCCHSS [12], however, reported that mortality was significantly lower at hospital discharge in the corticosteroid group (39.0% vs 45.3% in untreated, p = 0.02). Compared with our study, trial participants had higher baseline total SOFA scores, controls had higher mortality rates (45.3% vs 12.0%), and trial participants received more vasopressors. There were also notable differences in how corticosteroid treatments were administered. A systematic review and meta-analysis [4] published in 2019 found that pooled in-hospital mortality from 19 RCTs was lower in the corticosteroid treatment group (Risk Ratio, 0.88; 95%CI:0.79–0.99). Nevertheless, even among those pooled RCTs, only 2 out of 19 articles [12, 50] reported significantly lower in-hospital mortality in corticosteroid groups: APROCCHSS and another study published in 1976, which may have been subject to dramatic changes in standards of care.

Our findings could be further explained in several potential ways. First, there were relatively few treated patients; only 562 and 465 received the treatment in the explicit and Sepsis-3 cohort. Nonetheless, this sample size was still larger than most existing RCTs [26, 29, 31, 42–44], and the overall prevalence of severe sepsis and septic shock are in line with national estimates in ICUs [51]. Second, potential effect modifiers such as adrenal insufficiency, C-Reactive Protein (CRP), or disease severity seemed to modify the findings [43, 44, 52]. Therefore, additional studies are necessary to further confirm the treatment effect modifiers of corticosteroids in patients with sepsis.

Our finding that survival seemed to be worse in the treatment group in the first few days remains unclear. One possible reason may be reduced cortisol breakdown in patients with sepsis [53], which could be leading to higher serum cortisol levels, which have been thought to be associated with mortality [54]. Thus, the interaction between cortisol metabolism and corticosteroid use may require further research.

### Clinical implications

To our knowledge, this is the first observational study focused on the effects of corticosteroids and in-hospital survival using Sepsis-3 criteria or using these methods to evaluate in-hospital survival [36]. As a result, absolute risks were reported as main estimates which may be more clinically relevant than hazard ratios [55]. According to the updated Surviving Sepsis Campaign guidelines [56], there has been no established evidence regarding the use of corticosteroids in septic patients defined by the Sepsis-3 criteria. Therefore, our findings can be used as updated evidence to help clinicians determine whether corticosteroid administration was necessary for patients with sepsis or septic shock defined by the Sepsis-3 criteria.

### Limitations

This study has some potential limitations. First, using explicit diagnosis codes may have potentially included patients who actually did not have sepsis [15]. However, prior evidence has shown that the ICD-9 codes had very high PPV, so we believe the magnitude of the misclassification is limited and moreover unlikely to be differential between groups. Second, sicker patients tend to receive corticosteroids, and our study may suffer from confounding by indication even though we adjusted for numerous baseline confounders. However, the sensitivity analysis to explore this issue comparing the treated to those who received any dose of corticosteroids still agreed with the primary result. Third, although excluding patients who died within the first 96 hours successfully eliminated immortal time bias [28], this adjustment also introduced selection bias to our findings, limiting generalizability [57]. To minimize this, we conducted two additional sensitivity analyses shifting the exposure windows and changing immortal time adjustments, and the results were consistent with the primary approach. Fourth, we utilized sepsis onset as an anchor to calculate the SOFA score; while this may not perfectly represent Sepsis-3 definitions, sensitivity analyses changing SOFA score assessment windows were generally consistent. Fifth in Table 3, the adjusted survival of “all patients” was similar to the subgroup of patients with septic shock only, suggesting that the algorithm may identify patients with more critical conditions, leading to less generalizability. Sixth, the eICU database only contained patients admitted to ICUs from 2014 to 2015, which may limit generalizability to other years; some lab values were untested, and we assumed untested data to be normal. Finally, while we adjusted for numerous patient-level and facility-level factors, unmeasured confounding may affect the findings.

## Conclusions

In patients with sepsis or septic shock, intravenous corticosteroids were not associated with a higher in-hospital survival, and the effectiveness of corticosteroid treatment was not modified by different sepsis definitions.

## Supporting information

S1 Table SOFA score search strategy. (DOCX)

S2 Table SOFA score calculation. (DOCX)

S3 Table Search strategy for medical history and comorbidities. (DOCX)

S4 Table Search strategy for lab data. (DOCX)

S5 Table Search strategy for the demographic classification. (DOCX)

S6 Table Search strategy for corticosteroids. (DOCX)

S7 Table Sequence number of corticosteroids. (DOCX)

S8 Table Frequency of corticosteroids. (DOCX)

S9 Table Dosage of corticosteroids. (DOCX)

S10 Table Calculations of the corticosteroid treatments. (DOCX)

S11 Table Percentage of untested patients for each laboratory values. (DOCX)

S12 Table Baseline characteristics of the six subcategories of SOFA score in the explicit cohort. (DOCX)

S13 Table Baseline characteristics of the six subcategories of SOFA score in the Sepsis-3 cohort. (DOCX)

S14 Table Sensitivity analyses in the explicit cohort. Abbreviations: IPTW, inverse probability treatment weight; ^a^ with 2-day immortal time bias adjustment; ^b^ with 6-day immortal time bias adjustment; * Stepwise model selection was used.
(DOCX)

S15 Table Sensitivity analyses in the Sepsis-3 cohort. Abbreviations: IPTW, inverse probability treatment weight; ^a^ with 2-day immortal time bias adjustment; ^b^ with 6-day immortal time bias adjustment; * Stepwise model selection was used.
(DOCX)

S1 Fig Diagram. (DOCX)

S2 Fig Lengths of follow-up (days). a. Lengths of follow-up for the Explicit Cohort; b. Lengths of follow-up for the Sepsis-3 Cohort.
(DOCX)

S3 Fig In-hospital survival and survival difference between groups stratified by disease severity after IPTW in the explicit sepsis cohort. A. In-hospital Survival up to 50 days among those with septic shock diagnosis codes; B. In-hospital Survival up to 50 days among those without septic shock diagnosis codes; C. In-hospital Survival Difference between treatment groups among those with septic shock diagnosis codes; D. In-hospital Survival Difference between treatment groups among those without septic shock diagnosis codes.
(DOCX)

S4 Fig In-hospital survival and survival difference between treated and non-treated from the sensitivity analyses in the explicit cohort. A. In-hospital Survival up to 50 days using 3-hour SOFA score assessment window; B. In-hospital Survival up to 50 days using 12-hour SOFA score assessment window; C. In-hospital Survival Difference using 3-hour SOFA score assessment window; D. In-hospital Survival Difference using 12-hour SOFA score assessment window; E. In-hospital Survival up to 50 days using 24-hour SOFA score assessment window; F. In-hospital Survival up to 50 days using 1-day exposure window; G. In-hospital Survival difference using 24-hour SOFA score assessment window; H. In-hospital Survival difference using 1-day exposure window; I. In-hospital Survival up to 50 days using 5-day SOFA score assessment window; J. In-hospital Survival up to 50 days using Corticosteroid daily dose 200 to 400mg; K. In-hospital Survival difference using 5-day SOFA score assessment window; L. In-hospital Survival difference using Corticosteroid daily dose 200 to 400mg; M. In-hospital Survival up to 50 days when individuals received no any corticosteroids as controls; N. In-hospital Survival up to 50 days when excluding patients with asthma or COPD; O. In-hospital Survival difference when individuals received no any corticosteroids as controls; P. In-hospital Survival difference when excluding patients with asthma or COPD; Q. In-hospital Survival up to 50 days when excluding hospitals that never prescribed corticosteroids; R. In-hospital Survival up to 50 days when using g-formula; S. In-hospital Survival difference when excluding hospitals that never prescribed corticosteroids; T. In-hospital Survival difference when using g-formula; U. In-hospital Survival up to 50 days using any dose of corticosteroids as controls; V. In-hospital Survival up to 50 days when excluding >1 hospitalization due to sepsis or septic shock within 2014–2015; W. In-hospital Survival difference using any dose of corticosteroids as controls; X. In-hospital Survival difference when excluding >1 hospitalization due to sepsis or septic shock within 2014–2015; Y. In-hospital Survival up to 90 days; Z. In-hospital Survival up to 50 days when excluding patients without ventilation support; AA. In-hospital Survival difference up to 90 days; AB. In-hospital Survival difference when excluding patients without ventilation support; AC. In-hospital Survival up to 50 days when excluding patients admitted for surgery; AD. In-hospital Survival difference when excluding patients admitted for surgery; AE. In-hospital Survival up to 50 days when using patients receiving hydrocortisone as treated; AF. In-hospital Survival difference when using patients receiving hydrocortisone as treated; AG. In-hospital Survival up to 50 days when all untested values assumed abnormal; AH. In-hospital Survival difference when all untested values assumed abnormal; AI. In-hospital Survival up to 50 days when excluding laboratory values with high percentage of untested patients from the analysis; AJ. In-hospital Survival difference when excluding laboratory values with high percentage of untested patients from the analysis.
(DOCX)

S5 Fig In-hospital survival and survival difference between groups stratified by disease severity after IPTW in the Sepsis-3 cohort. A. In-hospital Survival up to 50 days among those met the septic shock definition; B. In-hospital Survival up to 50 days among those did not meet the septic shock definition; C. In-hospital Survival Difference between treatment groups among those met the septic shock definition; D. In-hospital Survival Difference between treatment groups among those did not meet the septic shock definition.
(DOCX)

S6 Fig In-hospital survival and survival difference between treated and non-treated from the sensitivity analyses in the Sepsis-3 cohort. A. In-hospital Survival up to 50 days using 3-hour SOFA score assessment window; B. In-hospital Survival up to 50 days using 12-hour SOFA score assessment window; C. In-hospital Survival Difference using 3-hour SOFA score assessment window; D. In-hospital Survival Difference using 12-hour SOFA score assessment window; E. In-hospital Survival up to 50 days using 24-hour SOFA score assessment window; F. In-hospital Survival up to 50 days using 1-day exposure window; G. In-hospital Survival difference using 24-hour SOFA score assessment window; H. In-hospital Survival difference using 1-day exposure window; I. In-hospital Survival up to 50 days using 5-day SOFA score assessment window; J. In-hospital Survival up to 50 days using Corticosteroid daily dose 200 to 400mg; K. In-hospital Survival difference using 5-day SOFA score assessment window; L. In-hospital Survival difference using Corticosteroid daily dose 200 to 400mg; M. In-hospital Survival up to 50 days when individuals received no any corticosteroids as controls; N. In-hospital Survival up to 50 days when excluding patients with asthma or COPD; O. In-hospital Survival difference when individuals received no any corticosteroids as controls; P. In-hospital Survival difference when excluding patients with asthma or COPD; Q. In-hospital Survival up to 50 days when excluding hospitals that never prescribed corticosteroids; R. In-hospital Survival up to 50 days when using g-formula; S. In-hospital Survival difference when excluding hospitals that never prescribed corticosteroids; T. In-hospital Survival difference when using g-formula; U. In-hospital Survival up to 50 days using any dose of corticosteroids as controls; V. In-hospital Survival up to 50 days when excluding >1 hospitalization due to sepsis or septic shock within 2014–2015; W. In-hospital Survival difference using any dose of corticosteroids as controls; X. In-hospital Survival difference when excluding >1 hospitalization due to sepsis or septic shock within 2014–2015; Y. In-hospital Survival up to 90 days; Z. In-hospital Survival up to 50 days when excluding patients without ventilation support; AA. In-hospital Survival difference up to 90 days; AB. In-hospital Survival difference when excluding patients without ventilation support; AC. In-hospital Survival up to 50 days when excluding patients admitted for surgery; AD. In-hospital Survival difference when excluding patients admitted for surgery; AE. In-hospital Survival up to 50 days when using patients receiving hydrocortisone as treated; AF. In-hospital Survival difference when using patients receiving hydrocortisone as treated; AG. In-hospital Survival up to 50 days when all untested values assumed abnormal; AH. In-hospital Survival difference when all untested values assumed abnormal; AI. In-hospital Survival up to 50 days when excluding laboratory values with high percentage of untested patients from the analysis; AJ. In-hospital Survival difference when excluding laboratory values with high percentage of untested patients from the analysis.
(DOCX)
