# Supplementary material for: Effectiveness of corticosteroids in patients with sepsis or septic shock using the new third international consensus definitions (Sepsis-3): A retrospective observational study
Source: PLoS One. 2020 Dec 3;15(12):e0243149. doi: 10.1371/journal.pone.0243149 (PMC7714118; doi:10.1371/journal.pone.0243149)
Supplement: S1 Table — (DOCX) [file pone.0243149.s001.docx]

S1 Table. SOFA Score Search Strategy

| Respiration  (the table ‘lab’ in the eICU was used) | | |
| --- | --- | --- |
| labname | paO2 | FiO2 |
| Respiration  (the table ‘respiratorycare’ in the eICU was used)  We assumed patients with records in this table means they received ventilation support. | | |
| Coagulation  (the table ‘lab’ in the eICU was used) | | |
| labname | platelets x 1000 | |
| Liver  (the table ‘lab’ in the eICU was used) | | |
| labname | total bilirubin | |
| Cardiovascular  (the table ‘nursecharting’ in the eICU was used for MAP) | | |
| nursingchartcelltypevallabel | Arterial Line MAP (mmHg) | MAP (mmHg) |
| Cardiovascular  (the table ‘medication’ in the eICU was used for catecholamine) | | |
| drugname | EPINEPHrine | NOREPINEPHRINE |
|  | EPINEPHrine 8 MG in 250 mL NS | NOREPINEPHRINE 4 MG/250 ML NS |
|  | NOREPINEPHRINE 8 MG in 250mL NS | PRMX NOREPInephrine 4 mg/250 mL NS Drip |
|  | NOREPINEPHRINE BITARTRATE 1 MG/ML AMP | NOREPINEPHRINE BITARTRATE 1 MG/ML IV : 4 ML |
|  | NOREPINEPHRINE BITARTRATE 1 MG/ML IJ SOLN | NOREPINEPHRINE BITARTRATE 1 MG/ML IV SOLN |
|  | <<norepinephrine 4 mg/4 mL Inj | 250 ML PLAS CONT : NOREPINEPHRINE IN D5W 8 MG/250 ML |
|  | DOPamine |  |
| drughiclseqno | 36346 | 2051 |
|  | 2050 | 34361 |
|  | 36437 | 2060 |
|  | 2059 |  |
| Cardiovascular  (the table ‘infusiondrug’ in the eICU was used for catecholamine) | | |
| drugname | DOBUTamine MAX 1000 mg Dextrose 5% 250 ml Premix (mcg/kg/min) | Dobutamine (mcg/kg/min) |
|  | DOBUTamine STD 500 mg Dextrose 5% 250 ml Premix (mcg/kg/min) | Dobutamine (mcg/min) |
|  | Dobutamine | Dobutamine (ml/hr) |
|  | Dobutamine () | Dobutamine (units/min) |
|  | Dobutamine (mcg/kg/hr) | dobutrex |
|  | dobutrex (mcg/kg/min) | dobutrex (mg/kg/min) |
|  | DOPamine MAX 800 mg Dextrose 5% 250 ml Premix (mcg/kg/min) | Dopamine (mcg/kg/hr) |
|  | DOPamine STD 15 mg Dextrose 5% 250 ml Premix (mcg/kg/min) | Dopamine (nanograms/kg/min) |
|  | DOPamine STD 400 mg Dextrose 5% 250 ml Premix (mcg/kg/min) | dopamine (mcg/kg/min) |
|  | DOPamine STD 400 mg Dextrose 5% 250 ml Premix (mcg/kg/min) | Dopamine |
|  | DOPamine STD 400 mg Dextrose 5% 500 ml Premix (mcg/kg/min) | Dopamine () |
|  | Dopamine (mcg/kg/min) | Dopamine (mg/hr) |
|  | Dopamine (Unknown) | Dopamine (ml/hr) |
|  | Dopamine (mcg/hr) | Norepinephrine (mcg/kg/hr) |
|  | Dopamine (mcg/min) | Norepinephrine (Unknown) |
|  | Norepinephrine (mcg/kg/min) | Norepinephrine (mcg/hr) |
|  | Norepinephrine (mg/kg/min) | Norepinephrine (mcg/min) |
|  | Levophed (mcg/kg/min) | Norepinephrine (mg/hr) |
|  | Norepinephrine | Norepinephrine (mg/min) |
|  | Norepinephrine () | Norepinephrine STD 32 mg Dextrose 5% 500 ml (mcg/min) |
|  | Norepinephrine (ml/hr) | Norepinephrine STD 4 mg Dextrose 5% 250 ml (mcg/min) |
|  | Norepinephrine (units/min) | Norepinephrine STD 4 mg Dextrose 5% 500 ml (mcg/min) |
|  | Norepinephrine MAX 32 mg Dextrose 5% 250 ml (mcg/min) | Norepinephrine STD 8 mg Dextrose 5% 250 ml (mcg/min) |
|  | Norepinephrine MAX 32 mg Dextrose 5% 500 ml (mcg/min) | Norepinephrine STD 8 mg Dextrose 5% 500 ml (mcg/min) |
|  | Norepinephrine STD 32 mg Dextrose 5% 282 ml (mcg/min) | NSS w/ levo/vaso (ml/hr) |
|  | norepinephrine Volume (ml) | NSS with LEVO (ml/hr) |
|  | norepinephrine Volume (ml) (ml/hr) | levophed (mcg/min) |
|  | Levophed (mcg/min) | levophed (mcg/min) |
|  | Levophed (mg/hr) | levophed (ml/hr) |
|  | Levophed (ml/hr) | EPINEPHrine(Adrenalin)STD 7 mg Sodium Chloride 0.9% 250 ml (mcg/min) |
|  | Epinephrine (mg/kg/min) | Epinepherine (mcg/min) |
|  | Epinephrine (mcg/kg/min) | Epinephrine |
|  | EPINEPHrine(Adrenalin)MAX 30 mg Sodium Chloride 0.9% 250 ml (mcg/min) | Epinephrine () |
|  | EPINEPHrine(Adrenalin)STD 4 mg Sodium Chloride 0.9% 250 ml (mcg/min) | Epinephrine (mcg/hr) |
|  | EPINEPHrine(Adrenalin)STD 4 mg Sodium Chloride 0.9% 500 ml (mcg/min) | Epinephrine (ml/hr) |
|  | Epinephrine (mcg/min) | Epinephrine (mg/hr) |
| Central nervous system  (the table ‘nursecharting’ in the eICU was used) | | |
| nursingchartcelltypevallabel | Glasgow coma score | |
| nursingchartcelltypevalname | GCS Total | |
| Renal  (the table ‘lab’ in the eICU was used for creatinine) | | |
| labname | creatinine | |
