# Supplementary material for: Effectiveness of corticosteroids in patients with sepsis or septic shock using the new third international consensus definitions (Sepsis-3): A retrospective observational study
Source: PLoS One. 2020 Dec 3;15(12):e0243149. doi: 10.1371/journal.pone.0243149 (PMC7714118; doi:10.1371/journal.pone.0243149)
Supplement: S2 Table — (DOCX) [file pone.0243149.s002.docx]

S2 Table. SOFA Score Calculation

| SOFA score calculation was generally based on the Sepsis-3 definition.^1^ | |
| --- | --- |
| SOFA score assessment window | 48 hours before the onset of sepsis. |
| Respiration | If PaO2 (mmHg) =0 then deleted;  The lowest value of PaO2 in the SOFA score assessment window was used for each patient;  If FiO2 (mmHg) <0.2 then deleted;  30mins before and after the examined time of the PaO2 was defined as FiO2 assessment window;  Any value of FiO2 examined in the FiO2 assessment window were used to calculate the ratio of PaO2/FiO2;  The lowest value of the ratio of PaO2/FiO2 was used to calculate the respiration SOFA score for each patient;  Respiratory support was defined as any ventilation supports started in the SOFA score assessment window;  If the ratio of PaO2/FiO2 >=400 in the SOFA score assessment window, the SOFA scores of respiration = 0;  If 300 <= the ratio of PaO2/FiO2 <400 in the SOFA score assessment window, the SOFA scores of respiration = 1;  If 200 <= the ratio of PaO2/FiO2 <300 in the SOFA score assessment window, the SOFA scores of respiration = 2;  If 200 <= the ratio of PaO2/FiO2 <300 in the SOFA score assessment window, the SOFA scores of respiration = 2;  If 0 <= the ratio of PaO2/FiO2 <200 without ventilation support in the SOFA score assessment window, the SOFA scores of respiration = 2;  If 100 <= the ratio of PaO2/FiO2 <200 with ventilation support in the SOFA score assessment window, the SOFA scores of respiration = 3;  If 0 <= the ratio of PaO2/FiO2 <100 with ventilation support in the SOFA score assessment window, the SOFA scores of respiration = 4;  SOFA scores of respiration were assumed 0 for those patients who did not have PaO2 or FiO2 data available in the SOFA assessment window.  SOFA scores of respiration were assumed 0 for those patients who had medical history of respiratory failure. |
| Coagulation | If lab value of platelet/μL =0 then deleted;  The lowest value of platelet in the SOFA score assessment window was used for each patient;  If the value of platelet >=150*10^3^/μL in the SOFA score assessment window, the SOFA scores of coagulation = 0;  If the value of platelet >=100*10^3^/μL and < 150*10^3^/μL in the SOFA score assessment window, the SOFA scores of coagulation = 1;  If the value of platelet >=50*10^3^/μL and < 100*10^3^/μL in the SOFA score assessment window, the SOFA scores of coagulation = 2;  If the value of platelet >=20*10^3^/μL and < 50*10/μL in the SOFA score assessment window, the SOFA scores of coagulation = 3;  If the value of platelet >0/μL and < 20*10^3^/μL in the SOFA score assessment window, the SOFA scores of coagulation = 4;  SOFA scores of coagulation were assumed 0 for those patients who did not have platelet data available in the SOFA assessment window. |
| Liver | If lab value of total bilirubin (μmol/L) =0 then deleted;  The highest value of total bilirubin in the SOFA score assessment window was used for each patient;  If the value of total bilirubin >0 μmol/L and < 1.2 μmol/L in the SOFA score assessment window, the Liver SOFA scores = 0;  If the value of total bilirubin >= 1.2 μmol/L and < 2 μmol/L in the SOFA score assessment window, the Liver SOFA scores = 1;  If the value of total bilirubin >= 2μmol/L and < 6 μmol/L in the SOFA score assessment window, the Liver SOFA scores = 2;  If the value of total bilirubin >= 6 μmol/L and < 12 μmol/L in the SOFA score assessment window, the Liver SOFA scores = 3;  If the value of total bilirubin >= 12 μmol/L in the SOFA score assessment window, the Liver SOFA scores = 4;  Liver SOFA scores were assumed 0 for those patients who did not have total bilirubin data available in the SOFA assessment window.  Liver SOFA scores were assumed 0 for those patients who had medical history of liver cirrhosis. |
| Cardiovascular | If lab value of Mean Arterial Pressure (mmHg) <30 then deleted;  The lowest value of platelet in the SOFA score assessment window was used for each patient;  If MAP >=70 mmHg without any catecholamine use in the SOFA score assessment window, Cardiovascular SOFA score=0;  If MAP <70 mmHg without any catecholamine use in the SOFA score assessment window, Cardiovascular SOFA score=1;  If drug rate of dobutamine=0 then deleted;  Any dobutamine used in the SOFA score assessment window was defined as Cardiovascular SOFA score=2;  If drug rate of dopamine=0 then deleted;  Any dopamine used in the SOFA score assessment window without clearly stated the rate (mcg/kg/min) was defined as Cardiovascular SOFA score=2;  Any dopamine used in the SOFA score assessment window with the rate <5.1 (mcg/kg/min) was defined as Cardiovascular SOFA score=2;  Any dopamine used in the SOFA score assessment window with the rate >=5.1 (mcg/kg/min) and <=15 (mcg/kg/min) was defined as Cardiovascular SOFA score=3;  Any dopamine used in the SOFA score assessment window with the rate > 15 (mcg/kg/min) was defined as Cardiovascular SOFA score=4;  If drug rate of epinephrine=0 then deleted;  Any epinephrine used in the SOFA score assessment window without clearly stated the rate (mcg/kg/min) was defined as Cardiovascular SOFA score=3;  Any epinephrine used in the SOFA score assessment window with the rate <=0.1 (mcg/kg/min) was defined as Cardiovascular SOFA score=3;  Any epinephrine used in the SOFA score assessment window with the rate > 0.1 (mcg/kg/min) was defined as Cardiovascular SOFA score=4;  If drug rate of norepinephrine=0 then deleted;  Any norepinephrine used in the SOFA score assessment window without clearly stated the rate (mcg/kg/min) was defined as Cardiovascular SOFA score=3;  Any norepinephrine used in the SOFA score assessment window with the rate <=0.1 (mcg/kg/min) was defined as Cardiovascular SOFA score=3;  Any norepinephrine used in the SOFA score assessment window with the rate > 0.1 (mcg/kg/min) was defined as Cardiovascular SOFA score=4;  Cardiovascular SOFA scores were assumed 0 for those patients who did not have MAP and any catecholamine data available in the SOFA assessment window. |
| Central nervous system | If value of total GCS score <3 then deleted;  The lowest value of total GCS score in the SOFA score assessment window was used for each patient;  If total GCS score =15 in the SOFA score assessment window, SOFA scores of Central nervous system =0;  If 13 <= total GCS score <= 14 in the SOFA score assessment window, SOFA scores of Central nervous system =1;  If 10 <= total GCS score <= 12 in the SOFA score assessment window, SOFA scores of Central nervous system =2;  If 6 <= total GCS score <=9 in the SOFA score assessment window, SOFA scores of Central nervous system =3;  If 2 < total GCS score <6 in the SOFA score assessment window, SOFA scores of Central nervous system =4;  SOFA scores of Central nervous system were assumed 0 for those patients who did not have total GCS score data available in the SOFA assessment window as well as those who were sedated. |
| Renal | If lab value of creatinine mg/dL =0 then deleted;  The highest value of creatinine in the SOFA score assessment window was used for each patient;  If 0 mg/dL < value of creatinine <1.2 mg/dL in the SOFA score assessment window, SOFA scores of Renal SOFA scores =0;  If 1.2 mg/dL <= value of creatinine <2.0 mg/dL in the SOFA score assessment window, SOFA scores of Renal SOFA scores =1;  If 2.0 mg/dL <= value of creatinine <3.5 mg/dL in the SOFA score assessment window, SOFA scores of Renal SOFA scores =2;  If 3.5 mg/dL <= value of creatinine <5 mg/dL in the SOFA score assessment window, SOFA scores of Renal SOFA scores =3;  If value of creatinine >=5 mg/dL in the SOFA score assessment window, SOFA scores of Renal SOFA scores =4;  Urine output was not used in the Renal SOFA score calculation;  Renal SOFA scores were assumed 0 for those patients who did not have creatinine data available in the SOFA assessment window.  Renal SOFA scores were assumed 0 for those patients who had medical history of renal failure. |

Reference:

1. Singer, Mervyn, et al. "The third international consensus definitions for sepsis and septic shock (Sepsis-3)." Jama 315.8 (2016): 801-810.
