# Supplementary material for: Effectiveness of corticosteroids in patients with sepsis or septic shock using the new third international consensus definitions (Sepsis-3): A retrospective observational study
Source: PLoS One. 2020 Dec 3;15(12):e0243149. doi: 10.1371/journal.pone.0243149 (PMC7714118; doi:10.1371/journal.pone.0243149)
Supplement: S3 Table — (DOCX) [file pone.0243149.s003.docx]

S3 Table. Search Strategy for Medical History and Comorbidities

| **Organ transplantation:**  Pasthistorypath in  ('notes/Progress Notes/Past History/Organ Systems/Cardiovascular (R)/s/p Heart Transplant/s/p heart transplant'  ,'notes/Progress Notes/Past History/Organ Systems/Gastrointestinal (R)/s/p Liver Transplant/s/p liver transplant'  ,'notes/Progress Notes/Past History/Organ Systems/Pulmonary/s/p Lung Transplant/s/p lung transplant'  ,'notes/Progress Notes/Past History/Organ Systems/Renal (R)/s/p Renal Transplant/s/p renal transplant') |
| --- |
| **Immunosuppression or recent steroid use:**  Pasthistorypath in  ('notes/Progress Notes/Past History/Organ Systems/Endocrine (R)/Recent Steroid Use (for > 10 days)/recent steroid use for > 10 days'  ,'notes/Progress Notes/Past History/Organ Systems/Infectious Disease (R)/Immunosuppression within past 6 months/>= 20 mg prednisone per day or equivalent'  ,'notes/Progress Notes/Past History/Organ Systems/Infectious Disease (R)/Immunosuppression within past 6 months/both prednisone and other immunosuppressive medications'  ,'notes/Progress Notes/Past History/Organ Systems/Infectious Disease (R)/Immunosuppression within past 6 months/other immunosuppressive medications'  ) |
| **Cancer therapy:**  pasthistorypath in  ('notes/Progress Notes/Past History/Organ Systems/Hematology/Oncology (R)/Cancer Therapy/Chemotherapy/Alkylating agents (bleomycin, cytoxan, cyclophos.)'  ,'notes/Progress Notes/Past History/Organ Systems/Hematology/Oncology (R)/Cancer Therapy/Chemotherapy/Anthracyclines (adriamycin, daunorubicin)'  ,'notes/Progress Notes/Past History/Organ Systems/Hematology/Oncology (R)/Cancer Therapy/Chemotherapy/BMT within past 12 mos.'  ,'notes/Progress Notes/Past History/Organ Systems/Hematology/Oncology (R)/Cancer Therapy/Chemotherapy/chemotherapy within past 6 mos.'  ,'notes/Progress Notes/Past History/Organ Systems/Hematology/Oncology (R)/Cancer Therapy/Chemotherapy/chemotherapy within past mo.'  ,'notes/Progress Notes/Past History/Organ Systems/Hematology/Oncology (R)/Cancer Therapy/Chemotherapy/Cis-platinum'  ,'notes/Progress Notes/Past History/Organ Systems/Hematology/Oncology (R)/Cancer Therapy/Chemotherapy/Vincristine'  ,'notes/Progress Notes/Past History/Organ Systems/Hematology/Oncology (R)/Cancer Therapy/Radiation Therapy within past 6 months/bone'  ,'notes/Progress Notes/Past History/Organ Systems/Hematology/Oncology (R)/Cancer Therapy/Radiation Therapy within past 6 months/brain'  ,'notes/Progress Notes/Past History/Organ Systems/Hematology/Oncology (R)/Cancer Therapy/Radiation Therapy within past 6 months/liver'  ,'notes/Progress Notes/Past History/Organ Systems/Hematology/Oncology (R)/Cancer Therapy/Radiation Therapy within past 6 months/lung'  ,'notes/Progress Notes/Past History/Organ Systems/Hematology/Oncology (R)/Cancer Therapy/Radiation Therapy within past 6 months/nodes'  ,'notes/Progress Notes/Past History/Organ Systems/Hematology/Oncology (R)/Cancer Therapy/Radiation Therapy within past 6 months/other'  ,'notes/Progress Notes/Past History/Organ Systems/Hematology/Oncology (R)/Cancer Therapy/Radiation Therapy within past 6 months/primary site'  ) |
| **Metastatic cancers or Hematological cancers:**  pasthistorypath in  ('notes/Progress Notes/Past History/Organ Systems/Hematology/Oncology (R)/Cancer/Hematologic Malignancy/ALL'  ,'notes/Progress Notes/Past History/Organ Systems/Hematology/Oncology (R)/Cancer/Hematologic Malignancy/AML'  ,'notes/Progress Notes/Past History/Organ Systems/Hematology/Oncology (R)/Cancer/Hematologic Malignancy/CLL'  ,'notes/Progress Notes/Past History/Organ Systems/Hematology/Oncology (R)/Cancer/Hematologic Malignancy/CML'  ,'notes/Progress Notes/Past History/Organ Systems/Hematology/Oncology (R)/Cancer/Hematologic Malignancy/Hodgkins disease'  ,'notes/Progress Notes/Past History/Organ Systems/Hematology/Oncology (R)/Cancer/Hematologic Malignancy/leukemia - other'  ,'notes/Progress Notes/Past History/Organ Systems/Hematology/Oncology (R)/Cancer/Hematologic Malignancy/multiple myeloma'  ,'notes/Progress Notes/Past History/Organ Systems/Hematology/Oncology (R)/Cancer/Hematologic Malignancy/non-Hodgkins lymphoma'  ,'notes/Progress Notes/Past History/Organ Systems/Hematology/Oncology (R)/Cancer/Hematologic Malignancy/other hematologic malignancy'  ,'notes/Progress Notes/Past History/Organ Systems/Hematology/Oncology (R)/Cancer/Metastases/bone'  ,'notes/Progress Notes/Past History/Organ Systems/Hematology/Oncology (R)/Cancer/Metastases/brain'  ,'notes/Progress Notes/Past History/Organ Systems/Hematology/Oncology (R)/Cancer/Metastases/carcinomatosis'  ,'notes/Progress Notes/Past History/Organ Systems/Hematology/Oncology (R)/Cancer/Metastases/intra-abdominal'  ,'notes/Progress Notes/Past History/Organ Systems/Hematology/Oncology (R)/Cancer/Metastases/liver'  ,'notes/Progress Notes/Past History/Organ Systems/Hematology/Oncology (R)/Cancer/Metastases/lung'  ,'notes/Progress Notes/Past History/Organ Systems/Hematology/Oncology (R)/Cancer/Metastases/nodes'  ,'notes/Progress Notes/Past History/Organ Systems/Hematology/Oncology (R)/Cancer/Metastases/other'  ) |
| **HIV infection:**  pasthistorypath in  ('notes/Progress Notes/Past History/Organ Systems/Infectious Disease (R)/AIDS/AIDS'  ,'notes/Progress Notes/Past History/Organ Systems/Infectious Disease (R)/HIV (only)/HIV positive') |
| **Autoimmune disease:**  pasthistorypath in  ('notes/Progress Notes/Past History/Organ Systems/Rheumatic/Dermato/Polymyositis/dermatomyositis'  ,'notes/Progress Notes/Past History/Organ Systems/Rheumatic/Rheumatoid Arthritis/rheumatoid arthritis'  ,'notes/Progress Notes/Past History/Organ Systems/Rheumatic/Scleroderma/scleroderma'  ,'notes/Progress Notes/Past History/Organ Systems/Rheumatic/SLE/SLE'  ,'notes/Progress Notes/Past History/Organ Systems/Rheumatic/Vasculitis/vasculitis') |
| **Arrhythmia:**  pasthistorypath in  ('notes/Progress Notes/Past History/Organ Systems/Cardiovascular (R)/Arrhythmias/atrial fibrillation - chronic'  ,'notes/Progress Notes/Past History/Organ Systems/Cardiovascular (R)/Arrhythmias/atrial fibrillation - intermittent'  ,'notes/Progress Notes/Past History/Organ Systems/Cardiovascular (R)/Arrhythmias/MAT'  ,'notes/Progress Notes/Past History/Organ Systems/Cardiovascular (R)/Arrhythmias/sick sinus syndrome'  ,'notes/Progress Notes/Past History/Organ Systems/Cardiovascular (R)/Arrhythmias/SVT- other'  ,'notes/Progress Notes/Past History/Organ Systems/Cardiovascular (R)/Arrhythmias/ventricular ectopy'  ,'notes/Progress Notes/Past History/Organ Systems/Cardiovascular (R)/Arrhythmias/ventricular fibrillation'  ,'notes/Progress Notes/Past History/Organ Systems/Cardiovascular (R)/Arrhythmias/ventricular tachycardia') |
| **Coronary artery diseases:**  pasthistorypath in  ('notes/Progress Notes/Past History/Organ Systems/Cardiovascular (R)/Angina/angina'  ,'notes/Progress Notes/Past History/Organ Systems/Cardiovascular (R)/Angina/angina - class I'  ,'notes/Progress Notes/Past History/Organ Systems/Cardiovascular (R)/Angina/angina - class II'  ,'notes/Progress Notes/Past History/Organ Systems/Cardiovascular (R)/Angina/angina - class III'  ,'notes/Progress Notes/Past History/Organ Systems/Cardiovascular (R)/Angina/angina - class IV'  ,'notes/Progress Notes/Past History/Organ Systems/Cardiovascular (R)/Angina/angina - severity unknown'  ,'notes/Progress Notes/Past History/Organ Systems/Cardiovascular (R)/Coronary Artery Bypass/CABG - date unknown'  ,'notes/Progress Notes/Past History/Organ Systems/Cardiovascular (R)/Coronary Artery Bypass/CABG - remote'  ,'notes/Progress Notes/Past History/Organ Systems/Cardiovascular (R)/Coronary Artery Bypass/CABG - within 2 years'  ,'notes/Progress Notes/Past History/Organ Systems/Cardiovascular (R)/Coronary Artery Bypass/CABG - within 5 years'  ,'notes/Progress Notes/Past History/Organ Systems/Cardiovascular (R)/Coronary Artery Bypass/CABG - within 6 months'  ,'notes/Progress Notes/Past History/Organ Systems/Cardiovascular (R)/Coronary Artery Bypass/multiple/multiple'  ,'notes/Progress Notes/Past History/Organ Systems/Cardiovascular (R)/Myocardial Infarction/MI - date unknown'  ,'notes/Progress Notes/Past History/Organ Systems/Cardiovascular (R)/Myocardial Infarction/MI - remote'  ,'notes/Progress Notes/Past History/Organ Systems/Cardiovascular (R)/Myocardial Infarction/MI - within 2 years'  ,'notes/Progress Notes/Past History/Organ Systems/Cardiovascular (R)/Myocardial Infarction/MI - within 5 years'  ,'notes/Progress Notes/Past History/Organ Systems/Cardiovascular (R)/Myocardial Infarction/MI - within 6 months'  ,'notes/Progress Notes/Past History/Organ Systems/Cardiovascular (R)/Myocardial Infarction/multiple/multiple'  ,'notes/Progress Notes/Past History/Organ Systems/Cardiovascular (R)/Procedural Coronary Intervention/multiple/multiple'  ,'notes/Progress Notes/Past History/Organ Systems/Cardiovascular (R)/Procedural Coronary Intervention/procedural coronary intervention - date unknown'  ,'notes/Progress Notes/Past History/Organ Systems/Cardiovascular (R)/Procedural Coronary Intervention/procedural coronary intervention - remote'  ,'notes/Progress Notes/Past History/Organ Systems/Cardiovascular (R)/Procedural Coronary Intervention/procedural coronary intervention - within 2 years'  ,'notes/Progress Notes/Past History/Organ Systems/Cardiovascular (R)/Procedural Coronary Intervention/procedural coronary intervention - within 5 years'  ,'notes/Progress Notes/Past History/Organ Systems/Cardiovascular (R)/Procedural Coronary Intervention/procedural coronary intervention - within 6 months') |
| **Heart failure:**  pasthistorypath in  ('notes/Progress Notes/Past History/Organ Systems/Cardiovascular (R)/Congestive Heart Failure/CHF'  ,'notes/Progress Notes/Past History/Organ Systems/Cardiovascular (R)/Congestive Heart Failure/CHF - class I'  ,'notes/Progress Notes/Past History/Organ Systems/Cardiovascular (R)/Congestive Heart Failure/CHF - class II'  ,'notes/Progress Notes/Past History/Organ Systems/Cardiovascular (R)/Congestive Heart Failure/CHF - class III'  ,'notes/Progress Notes/Past History/Organ Systems/Cardiovascular (R)/Congestive Heart Failure/CHF - class IV'  ,'notes/Progress Notes/Past History/Organ Systems/Cardiovascular (R)/Congestive Heart Failure/CHF - severity unknown') |
| **Diabetes:**  pasthistorypath in  ('notes/Progress Notes/Past History/Organ Systems/Endocrine (R)/Insulin Dependent Diabetes/insulin dependent diabetes'  ,'notes/Progress Notes/Past History/Organ Systems/Endocrine (R)/Non-Insulin Dependent Diabetes/medication dependent'  ,'notes/Progress Notes/Past History/Organ Systems/Endocrine (R)/Non-Insulin Dependent Diabetes/non-medication dependent') |
| **Liver cirrhosis:**  pasthistorypath in  ('notes/Progress Notes/Past History/Organ Systems/Gastrointestinal (R)/Cirrhosis/ascites'  ,'notes/Progress Notes/Past History/Organ Systems/Gastrointestinal (R)/Cirrhosis/biopsy proven'  ,'notes/Progress Notes/Past History/Organ Systems/Gastrointestinal (R)/Cirrhosis/clinical diagnosis'  ,'notes/Progress Notes/Past History/Organ Systems/Gastrointestinal (R)/Cirrhosis/coma'  ,'notes/Progress Notes/Past History/Organ Systems/Gastrointestinal (R)/Cirrhosis/encephalopathy'  ,'notes/Progress Notes/Past History/Organ Systems/Gastrointestinal (R)/Cirrhosis/jaundice'  ,'notes/Progress Notes/Past History/Organ Systems/Gastrointestinal (R)/Cirrhosis/UGI bleeding'  ,'notes/Progress Notes/Past History/Organ Systems/Gastrointestinal (R)/Cirrhosis/varices') |
| **Peptic ulcer:**  pasthistorypath in  ('notes/Progress Notes/Past History/Organ Systems/Gastrointestinal (R)/Peptic Ulcer Disease/peptic ulcer disease'  ,'notes/Progress Notes/Past History/Organ Systems/Gastrointestinal (R)/Peptic Ulcer Disease/peptic ulcer disease with h/o GI bleeding') |
| **Cancer history:**  pasthistorypath in  ('notes/Progress Notes/Past History/Organ Systems/Hematology/Oncology (R)/Cancer/Cancer-Primary Site/bile duct'  ,'notes/Progress Notes/Past History/Organ Systems/Hematology/Oncology (R)/Cancer/Cancer-Primary Site/bladder'  ,'notes/Progress Notes/Past History/Organ Systems/Hematology/Oncology (R)/Cancer/Cancer-Primary Site/bone'  ,'notes/Progress Notes/Past History/Organ Systems/Hematology/Oncology (R)/Cancer/Cancer-Primary Site/brain'  ,'notes/Progress Notes/Past History/Organ Systems/Hematology/Oncology (R)/Cancer/Cancer-Primary Site/breast'  ,'notes/Progress Notes/Past History/Organ Systems/Hematology/Oncology (R)/Cancer/Cancer-Primary Site/colon'  ,'notes/Progress Notes/Past History/Organ Systems/Hematology/Oncology (R)/Cancer/Cancer-Primary Site/esophagus'  ,'notes/Progress Notes/Past History/Organ Systems/Hematology/Oncology (R)/Cancer/Cancer-Primary Site/head and neck'  ,'notes/Progress Notes/Past History/Organ Systems/Hematology/Oncology (R)/Cancer/Cancer-Primary Site/kidney'  ,'notes/Progress Notes/Past History/Organ Systems/Hematology/Oncology (R)/Cancer/Cancer-Primary Site/liver'  ,'notes/Progress Notes/Past History/Organ Systems/Hematology/Oncology (R)/Cancer/Cancer-Primary Site/lung'  ,'notes/Progress Notes/Past History/Organ Systems/Hematology/Oncology (R)/Cancer/Cancer-Primary Site/melanoma'  ,'notes/Progress Notes/Past History/Organ Systems/Hematology/Oncology (R)/Cancer/Cancer-Primary Site/none'  ,'notes/Progress Notes/Past History/Organ Systems/Hematology/Oncology (R)/Cancer/Cancer-Primary Site/other'  ,'notes/Progress Notes/Past History/Organ Systems/Hematology/Oncology (R)/Cancer/Cancer-Primary Site/ovary'  ,'notes/Progress Notes/Past History/Organ Systems/Hematology/Oncology (R)/Cancer/Cancer-Primary Site/pancreas - adenocarcinoma'  ,'notes/Progress Notes/Past History/Organ Systems/Hematology/Oncology (R)/Cancer/Cancer-Primary Site/pancreas - islet cell'  ,'notes/Progress Notes/Past History/Organ Systems/Hematology/Oncology (R)/Cancer/Cancer-Primary Site/prostate'  ,'notes/Progress Notes/Past History/Organ Systems/Hematology/Oncology (R)/Cancer/Cancer-Primary Site/sarcoma'  ,'notes/Progress Notes/Past History/Organ Systems/Hematology/Oncology (R)/Cancer/Cancer-Primary Site/stomach'  ,'notes/Progress Notes/Past History/Organ Systems/Hematology/Oncology (R)/Cancer/Cancer-Primary Site/testes'  ,'notes/Progress Notes/Past History/Organ Systems/Hematology/Oncology (R)/Cancer/Cancer-Primary Site/unknown'  ,'notes/Progress Notes/Past History/Organ Systems/Hematology/Oncology (R)/Cancer/Cancer-Primary Site/uterus') |
| **Stroke:**  pasthistorypath in  ('notes/Progress Notes/Past History/Organ Systems/Neurologic/Strokes/multiple/multiple'  ,'notes/Progress Notes/Past History/Organ Systems/Neurologic/Strokes/stroke - date unknown'  ,'notes/Progress Notes/Past History/Organ Systems/Neurologic/Strokes/stroke - remote'  ,'notes/Progress Notes/Past History/Organ Systems/Neurologic/Strokes/stroke - within 2 years'  ,'notes/Progress Notes/Past History/Organ Systems/Neurologic/Strokes/stroke - within 5 years'  ,'notes/Progress Notes/Past History/Organ Systems/Neurologic/Strokes/stroke - within 6 months'  ,'notes/Progress Notes/Past History/Organ Systems/Neurologic/TIA(s)/multiple/multiple'  ,'notes/Progress Notes/Past History/Organ Systems/Neurologic/TIA(s)/TIA(s) - date unknown'  ,'notes/Progress Notes/Past History/Organ Systems/Neurologic/TIA(s)/TIA(s) - remote'  ,'notes/Progress Notes/Past History/Organ Systems/Neurologic/TIA(s)/TIA(s) - within 2 years'  ,'notes/Progress Notes/Past History/Organ Systems/Neurologic/TIA(s)/TIA(s) - within 5 years'  ,'notes/Progress Notes/Past History/Organ Systems/Neurologic/TIA(s)/TIA(s) - within 6 months') |
| **Asthma or COPD:**  pasthistorypath in  ('notes/Progress Notes/Past History/Organ Systems/Pulmonary/Asthma/asthma'  ,'notes/Progress Notes/Past History/Organ Systems/Pulmonary/COPD/COPD - moderate'  ,'notes/Progress Notes/Past History/Organ Systems/Pulmonary/COPD/COPD - no limitations'  ,'notes/Progress Notes/Past History/Organ Systems/Pulmonary/COPD/COPD - severe') |
| **Respiratory failure:**  pasthistorypath in  ('notes/Progress Notes/Past History/Organ Systems/Pulmonary/Respiratory Failure/multiple/multiple'  ,'notes/Progress Notes/Past History/Organ Systems/Pulmonary/Respiratory Failure/respiratory failure - date unknown'  ,'notes/Progress Notes/Past History/Organ Systems/Pulmonary/Respiratory Failure/respiratory failure - remote'  ,'notes/Progress Notes/Past History/Organ Systems/Pulmonary/Respiratory Failure/respiratory failure - within 2 years'  ,'notes/Progress Notes/Past History/Organ Systems/Pulmonary/Respiratory Failure/respiratory failure - within 5 years'  ,'notes/Progress Notes/Past History/Organ Systems/Pulmonary/Respiratory Failure/respiratory failure - within 6 months') |
| **Renal failure:**  pasthistorypath in  ('notes/Progress Notes/Past History/Organ Systems/Renal (R)/Renal Failure/renal failure - hemodialysis'  ,'notes/Progress Notes/Past History/Organ Systems/Renal (R)/Renal Failure/renal failure - peritoneal dialysis'  ,'notes/Progress Notes/Past History/Organ Systems/Renal (R)/Renal Failure/renal failure- not currently dialyzed'  ,'notes/Progress Notes/Past History/Organ Systems/Renal (R)/Renal Insufficiency/renal insufficiency - baseline creatinine unknown'  ,'notes/Progress Notes/Past History/Organ Systems/Renal (R)/Renal Insufficiency/renal insufficiency - creatinine > 5'  ,'notes/Progress Notes/Past History/Organ Systems/Renal (R)/Renal Insufficiency/renal insufficiency - creatinine 2-3'  ,'notes/Progress Notes/Past History/Organ Systems/Renal (R)/Renal Insufficiency/renal insufficiency - creatinine 3-4'  ,'notes/Progress Notes/Past History/Organ Systems/Renal (R)/Renal Insufficiency/renal insufficiency - creatinine 4-5') |
| From the table ‘pasthistory’ in the eICU |
