# Supplementary material for: Effectiveness of corticosteroids in patients with sepsis or septic shock using the new third international consensus definitions (Sepsis-3): A retrospective observational study
Source: PLoS One. 2020 Dec 3;15(12):e0243149. doi: 10.1371/journal.pone.0243149 (PMC7714118; doi:10.1371/journal.pone.0243149)
Supplement: S4 Table — (DOCX) [file pone.0243149.s004.docx]

S4 Table. Search Strategy for Lab data

| Covariate name | Table in the eICU | labname |  |
| --- | --- | --- | --- |
| WBC | lab | WBC x 1000 |  |
| Blood sugar | lab | glucose |  |
| lactate | lab | lactate |  |
| ALT | lab | ALT (SGPT) |  |
| AST | lab | AST (SGOT) |  |
| ammonia | lab | ammonia |  |
| Band | lab | -bands |  |
| K | lab | potassium |  |
| Na | lab | sodium |  |
| Hb | lab | Hgb |  |
| troponin - I | lab | troponin - I |  |
| For all the covariates above, any lab values =0 were deleted | | | |
