# Supplementary material for: Effectiveness of corticosteroids in patients with sepsis or septic shock using the new third international consensus definitions (Sepsis-3): A retrospective observational study
Source: PLoS One. 2020 Dec 3;15(12):e0243149. doi: 10.1371/journal.pone.0243149 (PMC7714118; doi:10.1371/journal.pone.0243149)
Supplement: S5 Table — (DOCX) [file pone.0243149.s005.docx]

S5 Table. Search Strategy for the Demographic Classification

| Column name | Table in the eICU |
| --- | --- |
| numbedscategory | hospital |
| region | hospital |
