# Supplementary material for: Effectiveness of corticosteroids in patients with sepsis or septic shock using the new third international consensus definitions (Sepsis-3): A retrospective observational study
Source: PLoS One. 2020 Dec 3;15(12):e0243149. doi: 10.1371/journal.pone.0243149 (PMC7714118; doi:10.1371/journal.pone.0243149)
Supplement: S6 Table — (DOCX) [file pone.0243149.s006.docx]

S6 Table. Search Strategy for Corticosteroids

| From the Table ‘infusiondrug’ in the eICU | | | |
| --- | --- | --- | --- |
| drugname | Methylpredisolone (ml/hr) | drugname | hydrocortisone (ml/hr) |
|  | methylprednisolone (ml/hr) |  | Solumedrol (mg/kg/hr) |
|  | meTHYLpredNISolone (ml/hr) |  | SOLUMEDROL (ml/hr) |
|  | Methylprednisolone IVF infused (Unknown) |  | solumedrol (mg/hr) |
|  | Methylprednisone (ml/hr) |  | solumedrol (mg/kg/hr) |
| Drugname and drugrate not equal to ‘ERROR’ or 0 | | | |
| From the Table ‘Medication’ in the eICU | | | |
| drugname | 1 ML VIAL : DEXAMETHASONE SODIUM PHOSPHATE 4 MG/ML IJ SOLN | drugname | METHYLPREDNISOLONE |
|  | hydroCORTisone (PF) 100 MG INJ |  | methylPREDNISolone |
|  | HYDROCORTISONE NA SUCCINATE PF 100 MG IJ SOLR |  | methylPREDNISolone 125 MG INJ |
|  | HYDROCORTISONE SOD SUCCINATE |  | methylPREDNISolone 40 MG INJ |
|  | HYDROCORTISONE SOD SUCCINATE 100 MG IJ SOLR |  | methylPREDNISolone sod suc(PF) |
|  | METHYLPREDNISOLONE SOD SUCC |  | dexamethasone |
|  | METHYLPREDNISOLONE SODIUM SUCC 125 MG IJ SOLR |  | DEXAMETHASONE |
|  | METHYLPREDNISOLONE SODIUM SUCC 40 MG IJ SOLR |  | DEXAMETHASONE 4 MG/1ML INJECTION |
|  | PREDNISONE |  | SOLU-MEDROL |
|  | predniSONE |  | Solu-MEDROL (PF) |
|  | SoluMedrol |  | DELTASONE |
| drughiclseqno | 36875 | drughiclseqno | 38645 |
|  | 36808 |  | 34381 |
|  | 2876 |  | 2879 |
|  | 2866 |  | 2889 |
|  | 2888 |  | 2875 |
| routeadmin | IV Push | routeadmin | IVPB |
|  | IV |  | IV - brief infusion (injection) |
|  | INJ |  | IntraVENOUS |
|  | Injection |  | IV (injection) |
|  | IV PUSH |  | IVB |
|  | INTRAVENOU |  | INTRAVEN |
|  | INTRAVENOUS |  | IV (intravenous) |
|  | Intravenous |  | PERIPH IV |
|  | INF |  | IVP |
|  | Inj |  |  |
| (Drugname or drughiclseqno) and routeadmin and dosage is not missing | | | |
