# Supplementary material for: Effectiveness of corticosteroids in patients with sepsis or septic shock using the new third international consensus definitions (Sepsis-3): A retrospective observational study
Source: PLoS One. 2020 Dec 3;15(12):e0243149. doi: 10.1371/journal.pone.0243149 (PMC7714118; doi:10.1371/journal.pone.0243149)
Supplement: S7 Table — (DOCX) [file pone.0243149.s007.docx]

S7 Table. Sequence Number of Corticosteroids

| If drughiclseqno = 2866, the use of ‘hydrocortisone’ was assumed |
| --- |
| If drughiclseqno = 2875, the use of ‘methylprednisolone’ was assumed |
| If drughiclseqno = 2876, the use of ‘methylprednisolone’ was assumed |
| If drughiclseqno = 2888, the use of ‘dexamethasone’ was assumed |
| If drughiclseqno = 2889, the use of ‘dexamethasone’ was assumed |
| If drughiclseqno = 34381, the use of ‘dexamethasone’ was assumed |
| If drughiclseqno = 36808, the use of ‘methylprednisolone’ was assumed |
| If drughiclseqno = 36875, the use of ‘hydrocortisone’ was assumed |
