# Supplementary material for: Effectiveness of corticosteroids in patients with sepsis or septic shock using the new third international consensus definitions (Sepsis-3): A retrospective observational study
Source: PLoS One. 2020 Dec 3;15(12):e0243149. doi: 10.1371/journal.pone.0243149 (PMC7714118; doi:10.1371/journal.pone.0243149)
Supplement: S8 Table — (DOCX) [file pone.0243149.s008.docx]

S8 Table. Frequency of Corticosteroids

| Frequency of corticosteroids was recognized as *STAT*  if ‘frequency’ in the table ‘Medication’ were: | | | |
| --- | --- | --- | --- |
| .STK-MED | once 1 | ONCE PRN INDEF | ONCE: for 1 dose(s) |
| 1X | Once as needed | Once PRN X1 | onceprn: for 1 dose(s) |
| 1XONLY | Once as needed X1 | Once X1 | ONCERX |
| NOW: for 1 dose(s) | ONCE M479 | once,1000 INDEF | ONE |
| ONCE | Once PRN | once,1200 INDEF | ONE 1 |
| ONE TIME | ONETIME | Q5MIN | X1 M1139 |
| ONE TIME One Time | ONETIMEPRN | STAT | X1 M359 |
| ONE TIME One Time Stat | OT | X1 | X1 M419 |
| One Time Stat | PYX1TIME | X1 M1019 | X1 M539 |
| ONE TIME X1 | Q10MIN X2 | X1 M1079 | X1 M599 |
| X1 M659 | X1 M779 | X1 M899 | X1ED M14 |
| X1 M719 | X1 M839 | X1 M959 |  |
| Frequency of corticosteroids was recognized as *0.5 per day*  if ‘frequency’ in the table ‘Medication’ were: | | | |
| Q48H | q 48 hour | Every Other Day |  |
| Frequency of corticosteroids was recognized as *1 per day*  if ‘frequency’ in the table ‘Medication’ were: | | | |
| Daily | 3xDaily | DAILY (0900) | Daily Breakfast |
| .Q24H | 4x Daily | DAILY (1700) | DAILY D30 |
| 1xDaily | 4x Daily PRN | DAILY AC | Daily EARLY |
| 1xDaily ac | AC Daily Breakfast | Daily at 0600 | Daily PRN |
| 2xDaily | DAILY (0600) | Daily BEDTIME | Daily with breakfast |
| Daily with dinner | daily9: 0900 for dose(s) | H24 | q 24 hour |
| Daily X1 | DAILYB | HS | q 24 hour (daily) |
| Daily X2 | DAILYPRN | Nightly | Q breakfast |
| DAILY X3 | Every 24 hours | prnqd INDEF | Q Evening |
| DAILY,DAILY | Every evening | q 24 h | Q0600 |
| Q24H | Q24H X5 | q24hr (interval) | QAC breakfast |
| Q24H SCH | q24hr | Q24HRS | QAM |
| QAM AC | qd8,0800 INDEF | QDP | QHS (2100) |
| qd22,2200 INDEF | QDAY | QHS | QPM |
| Frequency of corticosteroids was recognized as *2 per day*  if ‘frequency’ in the table ‘Medication’ were: | | | |
| 12H | BID (09 17) | BID WC | bid: 0900,2100 for dose(s) |
| 2 times daily | BID AC | BID X10 | BID0600 |
| 2 times per day | BID M/E | BID X2 | bid8,0800,1800 INDEF |
| BID | BID M/N | BID X4 | bida,0800,2200 INDEF |
| BID (0800, 1600) | BID PRN | BID,TWICE A DAY | bidc,0800,2000 INDEF |
| BIDPRN | Every 12 hours scheduled | Q12 | Q12H PRN |
| BIDTX | q 12 hour | Q12H | Q12H SCH |
| Every 12 Hours | q 12 hour (BID) | Q12H (int) | Q12H X10 |
| Q12H X2 | q12hr | q12hr (interval) |  |
| Frequency of corticosteroids was recognized as *3 per day*  if ‘frequency’ in the table ‘Medication’ were: | | | |
| 3 times daily | prntid INDEF | q8h ,0600,1400,2200 INDEF | Q8H X2 |
| 8H | q 8 hour | Q8H (int) | Q8H X3 |
| Every 8 hours | q 8 hour PRN | Q8H PRN | Q8HPRN |
| Every 8 hours scheduled | Q8 | Q8H SCH | q8hr |
| every8hr | Q8H | Q8H X15 | q8hr (interval) |
| Q8HRS | TID (09 14 21) | TID PRN | tid,0800,1600,2200 INDEF |
| TID | TID PC | TID X3 | tid8,0800,1200,1800 INDEF |
| Frequency of corticosteroids was recognized as *4 per day*  if ‘frequency’ in the table ‘Medication’ were: | | | |
| 4 times per day | Every 6 hours scheduled | Q6H | Q6H X2 |
| 6H | q 6 hour | Q6H (int) | Q6H X4 |
| Every 6 hours | q 6 hour PRN | Q6H PRN | q6h,0000,0600,1200,1800 INDEF |
| Every 6 hours PRN | Q6 | Q6H SCH | Q6HP |
| Q6HPRN | QID | Resp q 6 hour | RQ6 |
| Q6HR | qid8,0800,1200,1800,2200 INDEF |  |  |
| Frequency of corticosteroids was recognized as *6 per day*  if ‘frequency’ in the table ‘Medication’ were: | | | |
| Q4H SCH | Q4H | Q4H PRN | Q4HPRN |
| q 4 hour | Q4HR | Q4H (int) | Q4HRS |
| Frequency of corticosteroids was recognized as *8 per day*  if ‘frequency’ in the table ‘Medication’ were: | | | |
| Q3H |  |  |  |
| Frequency of corticosteroids was recognized as *Undefined*  if ‘frequency’ in the table ‘Medication’ were: | | | |
| 0 | 1700 | as directed | CONTINUOUS |
| 10 | 2100 | As needed | NOW |
| 600 | 9,002,100 | As needed X1 | OC,0600 INDEF |
| 730 | 60,014,002,200 | AS-DIR | ONDEM |
| 900 | 90,015,002,100 | CONT INF | OTCOND |
| OTO | prn INDEF | PRN X999 | PRNP |
| PER PROTOCOL | PRN X1 | prn6 INDEF | See Admin Instructions |
| PRN | PRN X2 | prn8 INDEF | See Admin Instructions PRN |
| See Admin Notes | X2 | X3 | Missing |
| TITRATE |  |  |  |
