# Supplementary material for: Effectiveness of corticosteroids in patients with sepsis or septic shock using the new third international consensus definitions (Sepsis-3): A retrospective observational study
Source: PLoS One. 2020 Dec 3;15(12):e0243149. doi: 10.1371/journal.pone.0243149 (PMC7714118; doi:10.1371/journal.pone.0243149)
Supplement: S9 Table — (DOCX) [file pone.0243149.s009.docx]

S9 Table. Dosage of Corticosteroids

| Dosage =0 were deleted; |
| --- |
| If unit of dosage was ml without clearly stated the mg, we assumed 100mg/ml for hydrocortisone;  If unit of dosage was ml without clearly stated the mg, we assumed 4mg/ml for dexamethasone;  If unit of dosage was ml without clearly stated the mg, we assumed 40mg/ml for methylprednisolone;  If unit of dosage was EA without clearly stated the mg, we assumed 100mg per EA for hydrocortisone;  If unit of dosage was EA without clearly stated the mg, we assumed 4mg per EA for dexamethasone;  If unit of dosage was EA without clearly stated the mg, we assumed 40mg per EA for methylprednisolone;  If unit of dosage was VL without clearly stated the mg, we assumed 4mg per VL for dexamethasone;  If dose is missing, we assumed 100mg/ml for hydrocortisone;  If dose is missing, we assumed 4mg/ml for dexamethasone;  If dose is missing, we assumed 40mg/ml for methylprednisolone; |
| 1 mg methylprednisolone = 5 mg hydrocortisone;  1 mg dexamethasone = 26.7 mg hydrocortisone;  1 mg prednisolone = 4 mg hydrocortisone; |
