# Supplementary material for: Effectiveness of corticosteroids in patients with sepsis or septic shock using the new third international consensus definitions (Sepsis-3): A retrospective observational study
Source: PLoS One. 2020 Dec 3;15(12):e0243149. doi: 10.1371/journal.pone.0243149 (PMC7714118; doi:10.1371/journal.pone.0243149)
Supplement: S10 Table — (DOCX) [file pone.0243149.s010.docx]

S10 Table. Calculations of the Corticosteroid Treatments

| Duration of the Corticosteroid Treatments^a^ | Corticosteroid Stop Time - Corticosteroid Start Time |
| --- | --- |
| Daily Dose of the Corticosteroids (hydrocortisone equivalent) | Frequency*Dosage*Hydrocortisone Equivalent dose |

^a^ Negative Duration of the Corticosteroid Treatments were deleted.
