# Supplementary material for: Effectiveness of corticosteroids in patients with sepsis or septic shock using the new third international consensus definitions (Sepsis-3): A retrospective observational study
Source: PLoS One. 2020 Dec 3;15(12):e0243149. doi: 10.1371/journal.pone.0243149 (PMC7714118; doi:10.1371/journal.pone.0243149)
Supplement: S11 Table — (DOCX) [file pone.0243149.s011.docx]

S11 Table. Percentage of untested patients for each laboratory values

| Laboratory values | Number of untested patients | Percentage |
| --- | --- | --- |
| WBC | 4 | 0.05% |
| Blood sugar | 43 | 0.6% |
| Serum Lactate | 991 | 13.8% |
| ALT | 447 | 6.2% |
| AST | 450 | 6.3% |
| Serum Potassium | 26 | 0.36% |
| Serum Sodium | 19 | 0.26% |
| Hemoglobin | 28 | 0.39% |
| % of Band Neutrophils | 4154 | 68.6% |
| Serum Ammonia | 5903 | 82.5% |
| Troponin-I | 3129 | 43.7% |
