# Supplementary material for: Effectiveness of corticosteroids in patients with sepsis or septic shock using the new third international consensus definitions (Sepsis-3): A retrospective observational study
Source: PLoS One. 2020 Dec 3;15(12):e0243149. doi: 10.1371/journal.pone.0243149 (PMC7714118; doi:10.1371/journal.pone.0243149)
Supplement: S12 Table — (DOCX) [file pone.0243149.s012.docx]

S12 Table. Baseline Characteristics of the Six Subcategories of SOFA Score in the Explicit Cohort.

| Characteristic | Before IPTW | | | After IPTW | | |
| --- | --- | --- | --- | --- | --- | --- |
|  | Control  (n=6596) | Treated  (n=562) | Absolute Standard Difference | Control  (n=6596) | Treated  (n=562) | Absolute Standard Difference |
| Subcategories of SOFA score | | | | | | |
| Respiration, mean (SD) | 0.21 (0.68) | 0.42 (0.97) | 0.28 | 0.23 (0.71) | 0.23 (0.70) | 0.00 |
| Coagulation, mean (SD) | 0.42 (0.79) | 0.38 (0.75) | 0.07 | 0.42 (0.79) | 0.45 (0.83) | 0.05 |
| Liver, mean (SD) | 0.35 (0.75) | 0.38 (0.78) | 0.05 | 0.35 (0.76) | 0.35 (0.77) | 0.00 |
| Cardiovascular, mean (SD) | 0.90 (1.38) | 1.64 (1.28) | 0.35 | 0.96 (0.98) | 0.96 (1.39) | 0.01 |
| Central nervous system, mean (SD) | 0.65 (1.16) | 0.88 (1.32) | 0.15 | 0.67 (1.18) | 0.66 (1.16) | 0.01 |
| Renal, mean (SD) | 0.86 (1.09) | 0.87 (1.15) | 0.01 | 0.86 (1.09) | 0.85 (1.13) | 0.01 |
