# Supplementary material for: Effectiveness of corticosteroids in patients with sepsis or septic shock using the new third international consensus definitions (Sepsis-3): A retrospective observational study
Source: PLoS One. 2020 Dec 3;15(12):e0243149. doi: 10.1371/journal.pone.0243149 (PMC7714118; doi:10.1371/journal.pone.0243149)
Supplement: S13 Table — (DOCX) [file pone.0243149.s013.docx]

S13 Table. Baseline Characteristics of the Six Subcategories of SOFA Score in the Sepsis-3 Cohort.

| Characteristic | Before IPTW | | | After IPTW | | |
| --- | --- | --- | --- | --- | --- | --- |
|  | Control  (n=6596) | Treated  (n=562) | Absolute Standard Difference | Control  (n=6596) | Treated  (n=562) | Absolute Standard Difference |
| Subcategories of SOFA score | | | | | | |
| Respiration, mean (SD) | 0.30 (0.80) | 0.50 (1.04) | 0.22 | 0.32 (0.83) | 0.33 (0.81) | 0.01 |
| Coagulation, mean (SD) | 0.58 (0.90) | 0.46 (0.81) | 0.16 | 0.57 (0.89) | 0.60 (0.93) | 0.04 |
| Liver, mean (SD) | 0.49 (0.87) | 0.45 (0.84) | 0.06 | 0.48 (0.87) | 0.47 (0.87) | 0.01 |
| Cardiovascular, mean (SD) | 1.31 (1.50) | 1.98 (1.43) | 0.31 | 1.37 (1.51) | 1.35 (1.49) | 0.01 |
| Central nervous system, mean (SD) | 0.92 (1.31) | 1.05 (1.39) | 0.07 | 0.93 (1.32) | 0.91 (1.30) | 0.01 |
| Renal, mean (SD) | 1.15 (1.17) | 1.01 (1.20) | 0.10 | 1.13 (1.17) | 1.10 (1.23) | 0.02 |
