# Supplementary material for: Effectiveness of corticosteroids in patients with sepsis or septic shock using the new third international consensus definitions (Sepsis-3): A retrospective observational study
Source: PLoS One. 2020 Dec 3;15(12):e0243149. doi: 10.1371/journal.pone.0243149 (PMC7714118; doi:10.1371/journal.pone.0243149)
Supplement: S15 Table — Abbreviations: IPTW, inverse probability treatment weight; a with 2-day immortal time bias adjustment; b with 6-day immortal time bias adjustment; * Stepwise model selection was used. (DOCX) [file pone.0243149.s015.docx]

S15 Table. Sensitivity Analyses in the Sepsis-3 Cohort

|  | **3-hour SOFA assessment window** | **12-hour SOFA assessment window** | **24-hour SOFA assessment window** | **1-day exposure window^a^** | **5-day exposure window^b^** | **Compare to any dose of corticosteroids** |
| --- | --- | --- | --- | --- | --- | --- |
| **Before IPTW adjustment** | | | | | |  |
| In-hospital 50-Day Survival among Controls | 0.56  (n=3432) | 0.57  (n=4365) | 0.57  (n=4496) | 0.53  (n=5512) | 0.59  (n=3519) | 0.51  (n=343) |
| In-hospital 50-Day Survival among Treated | 0.50  (n=416) | 0.53  (n=462) | 0.53  (n=465) | 0.44  (n=350) | 0.55  (n=446) | 0.54  (n=465) |
| In-hospital 50-Day Survival Difference with 95%CI | -0.06  (-0.22, 0.10) | -0.04  (-0.17, 0.10) | -0.03  (-0.18, 0.11) | -0.08  (-0.29, 0.08) | -0.04  (-0.18, 0.10) | 0.03  (-0.15, 0.20) |
| **After IPTW adjustment** | | | | | |  |
| In-hospital 50-Day Survival among Controls | 0.56  (n=3432) | 0.57  (n=4365) | 0.56  (n=4496) | 0.52  (n=5512) | 0.59  (n=3519) | 0.50  (n=343) |
| In-hospital 50-Day Survival among Treated | 0.47  (n=416) | 0.55  (n=462) | 0.57  (n=465) | 0.51  (n=350) | 0.62  (n=446) | 0.54  (n=465) |
| In-hospital 50-Day Survival Difference with 95%CI | -0.09  (-0.34, 0.15) | -0.02  (-0.20, 0.16) | 0.01  (-0.19, 0.17) | -0.01^*^  (-0.22, 0.15) | 0.03  (-0.11, 0.16) | 0.04  (-0.13, 0.22) |
|  | **Corticosteroid daily dose 200 to 400mg** | **Without any corticosteroids as controls** | **Excluded patients with asthma or COPD** | **Only hospitals ever prescribed corticosteroids** | **g-formula** | **Excluded patients >1 hospitalization** |
| **Before IPTW adjustment** | | | | | |  |
| In-hospital 50-Day Survival among Controls | 0.56  (n=4725) | 0.57  (n=4201) | 0.57  (n=3688) | 0.60  (n=2960) | 0.56  (n=4544) | 0.55  (n=3936) |
| In-hospital 50-Day Survival among Treated | 0.51  (n=284) | 0.54  (n=465) | 0.52  (n=370) | 0.54  (n=465) | 0.53  (n=465) | 0.51  (n=392) |
| In-hospital 50-Day Survival Difference with 95%CI | -0.05  (-0.22, 0.11) | -0.03  (-0.20, 0.08) | -0.05  (-0.24, 0.10) | -0.06  (-0.20, 0.08) | -0.03  (-0.17, 0.10) | -0.04  (-0.19, 0.11) |
| **After IPTW adjustment** | | | | | |  |
| In-hospital 50-Day Survival among Controls | 0.56  (n=4725) | 0.57  (n=4201) | 0.57  (n=3688) | 0.59  (n=2960) | 0.57  (n=4544) | 0.54  (n=3936) |
| In-hospital 50-Day Survival among Treated | 0.56  (n=284) | 0.59  (n=465) | 0.59  (n=370) | 0.56  (n=465) | 0.57  (n=465) | 0.55  (n=392) |
| In-hospital 50-Day Survival Difference with 95%CI | 0.00^*^  (-0.17, 0.15) | 0.02  (-0.20, 0.16) | 0.02^*^  (-0.20, 0.19) | -0.03  (-0.22, 0.12) | 0.00  (-0.16, 0.12) | 0.01  (-0.21, 0.20) |

Abbreviations: IPTW, inverse probability treatment weight;

^a^ with 2-day immortal time bias adjustment; ^b^ with 6-day immortal time bias adjustment;

^*^ Stepwise model selection was used;

|  | **In-hospital survival up to 90 days** | **Excluding patients without ventilation support** | **Excluding patients admitted for surgery** | **Patients received hydrocortisone as treated** | **All untested laboratory data assumed abnormal** | **Excluding laboratory values with high percentage of untested patients from the analysis** |
| --- | --- | --- | --- | --- | --- | --- |
| **Before IPTW adjustment** | | | | | |  |
| In-hospital 50-Day Survival among Controls | 0.56  (n=4544) | 0.52  (n=1305) | 0.56  (n=4406) | 0.57  (n=4354) | 0.56  (n=4544) | 0.56  (n=4544) |
| In-hospital 50-Day Survival among Treated | 0.53  (n=465) | 0.53  (n=239) | 0.53  (n=457) | 0.52  (n=308) | 0.53  (n=465) | 0.53  (n=465) |
| In-hospital 50-Day Survival Difference with 95%CI | -0.03  (-0.19, 0.09) | 0.01  (-0.14, 0.17) | -0.03  (-0.19, 0.10) | -0.04  (-0.23, 0.11) | -0.03  (-0.17, 0.10) | -0.03  (-0.17, 0.10) |
| **After IPTW adjustment** | | | | | |  |
| In-hospital 50-Day Survival among Controls | 0.56  (n=4544) | 0.52  (n=1305) | 0.56  (n=4406) | 0.57  (n=4354) | 0.56  (n=4544) | 0.56  (n=4544) |
| In-hospital 50-Day Survival among Treated | 0.58  (n=465) | 0.58  (n=239) | 0.57  (n=457) | 0.61  (n=308) | 0.58  (n=465) | 0.57  (n=465) |
| In-hospital 50-Day Survival Difference with 95%CI | 0.02  (-0.55, 0.18) | 0.06^*^  (-0.12, 0.22) | 0.01  (-0.21, 0.17) | 0.04^*^  (-0.11, 0.20) | 0.02  (-0.19, 0.18) | 0.01  (-0.19, 0.17) |

Abbreviations: IPTW, inverse probability treatment weight;

^a^ with 2-day immortal time bias adjustment;

^b^ with 6-day immortal time bias adjustment;

^*^ Stepwise model selection was used;
