# Supplementary figures and images for: Effectiveness of corticosteroids in patients with sepsis or septic shock using the new third international consensus definitions (Sepsis-3): A retrospective observational study
Source: PLoS One. 2020 Dec 3;15(12):e0243149. doi: 10.1371/journal.pone.0243149 (PMC7714118; doi:10.1371/journal.pone.0243149)

S1 Fig. Diagram
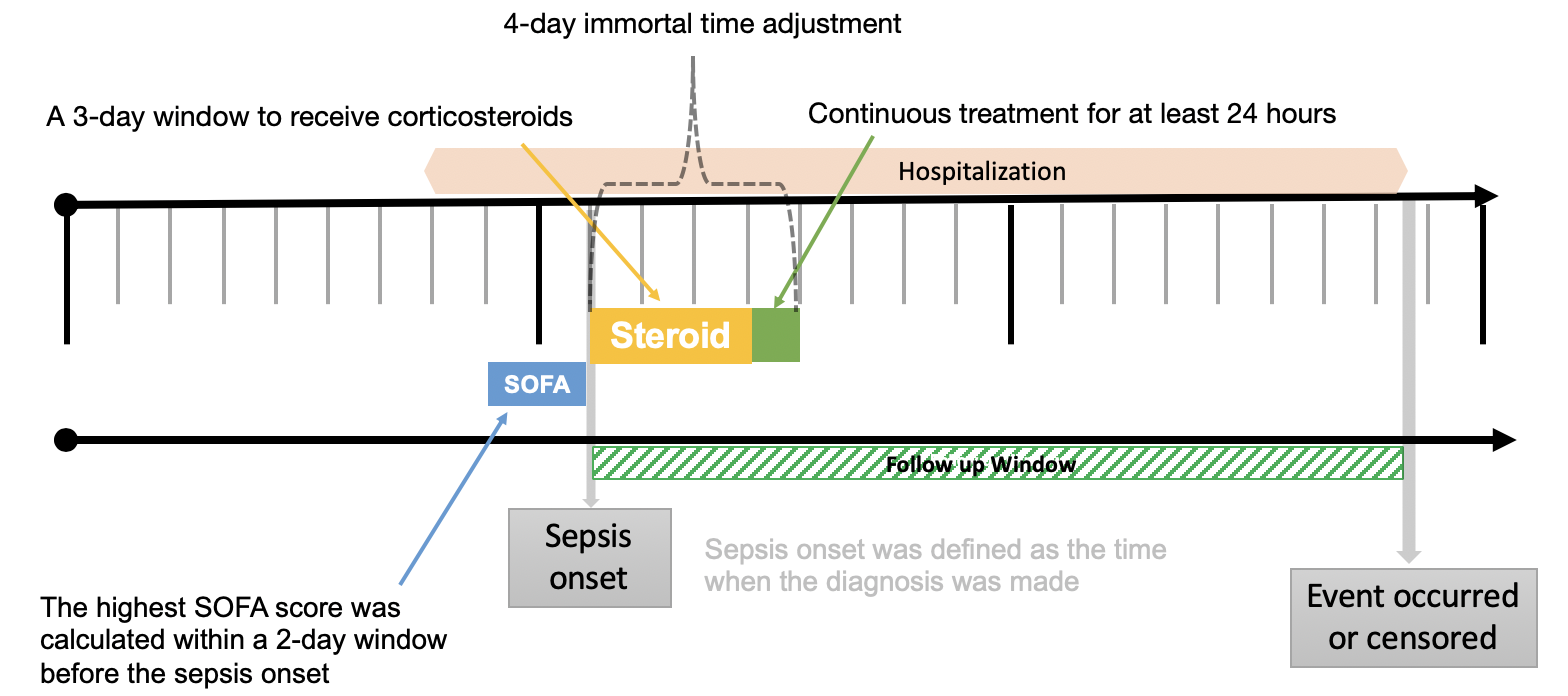

Supplement: S1 Fig — (DOCX) [file pone.0243149.s016.docx]
