# Supplementary material for: Effectiveness of corticosteroids in patients with sepsis or septic shock using the new third international consensus definitions (Sepsis-3): A retrospective observational study
Source: PLoS One. 2020 Dec 3;15(12):e0243149. doi: 10.1371/journal.pone.0243149 (PMC7714118; doi:10.1371/journal.pone.0243149)
Supplement: S2 Fig — a. Lengths of follow-up for the Explicit Cohort; b. Lengths of follow-up for the Sepsis-3 Cohort. (DOCX) [file pone.0243149.s017.docx]

S2 Fig. Lengths of Follow-up (Days)

| a.  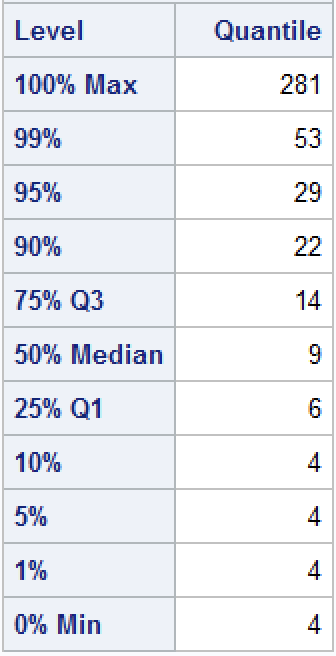 | b.  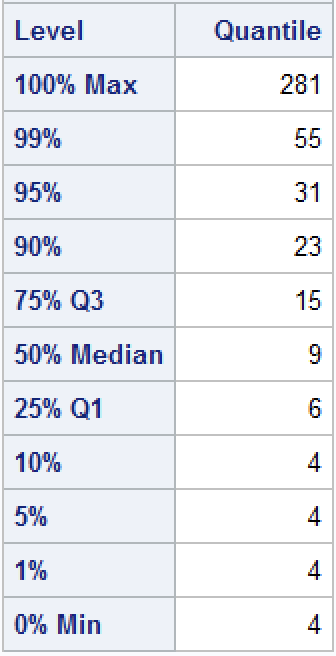 |
| --- | --- |
| a. Lengths of follow-up for the Explicit Cohort;  b. Lengths of follow-up for the Sepsis-3 Cohort. | |
