# Supplementary material for: Effectiveness of corticosteroids in patients with sepsis or septic shock using the new third international consensus definitions (Sepsis-3): A retrospective observational study
Source: PLoS One. 2020 Dec 3;15(12):e0243149. doi: 10.1371/journal.pone.0243149 (PMC7714118; doi:10.1371/journal.pone.0243149)
Supplement: S5 Fig — A. In-hospital Survival up to 50 days among those met the septic shock definition; B. In-hospital Survival up to 50 days among those did not meet the septic shock definition; C. In-hospital Survival Difference between treatment groups among those met the septic shock definition; D. In-hospital Survival Difference between treatment groups among those did not meet the septic shock definition. (DOCX) [file pone.0243149.s020.docx]

S5 Fig. In-hospital Survival and Survival Difference between Groups Stratified by Disease Severity after IPTW in the Sepsis-3 Cohort

| A.  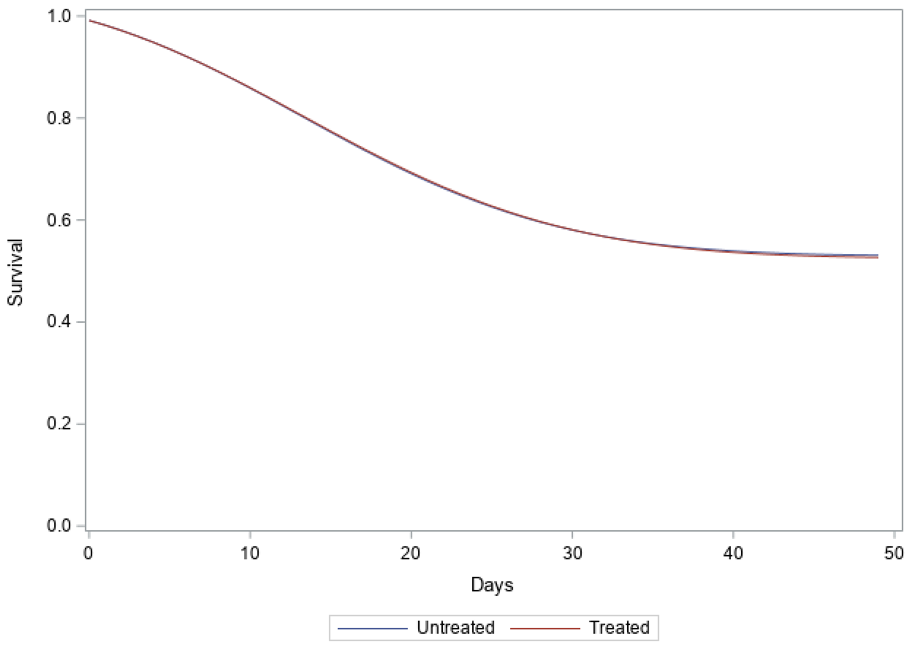 | B.  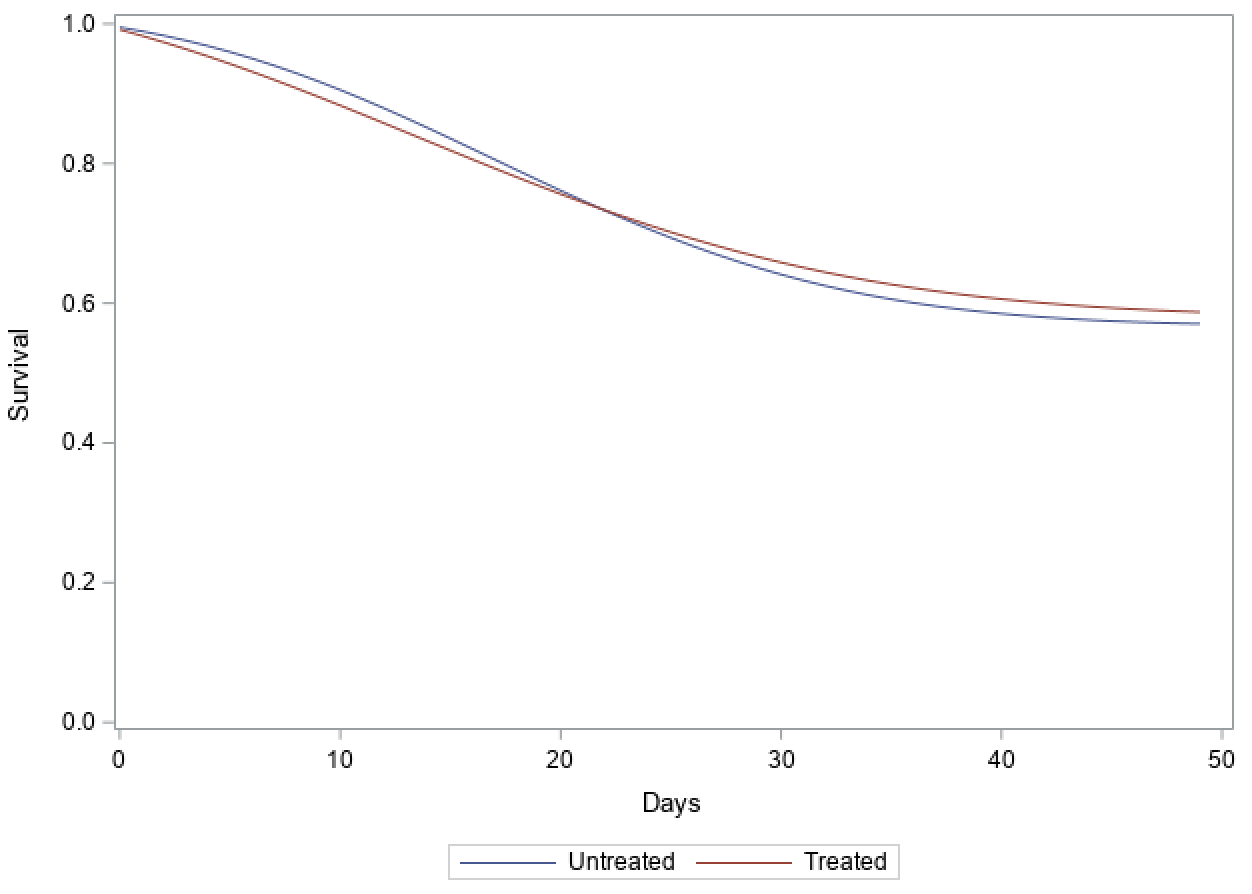 |
| --- | --- |
| C.  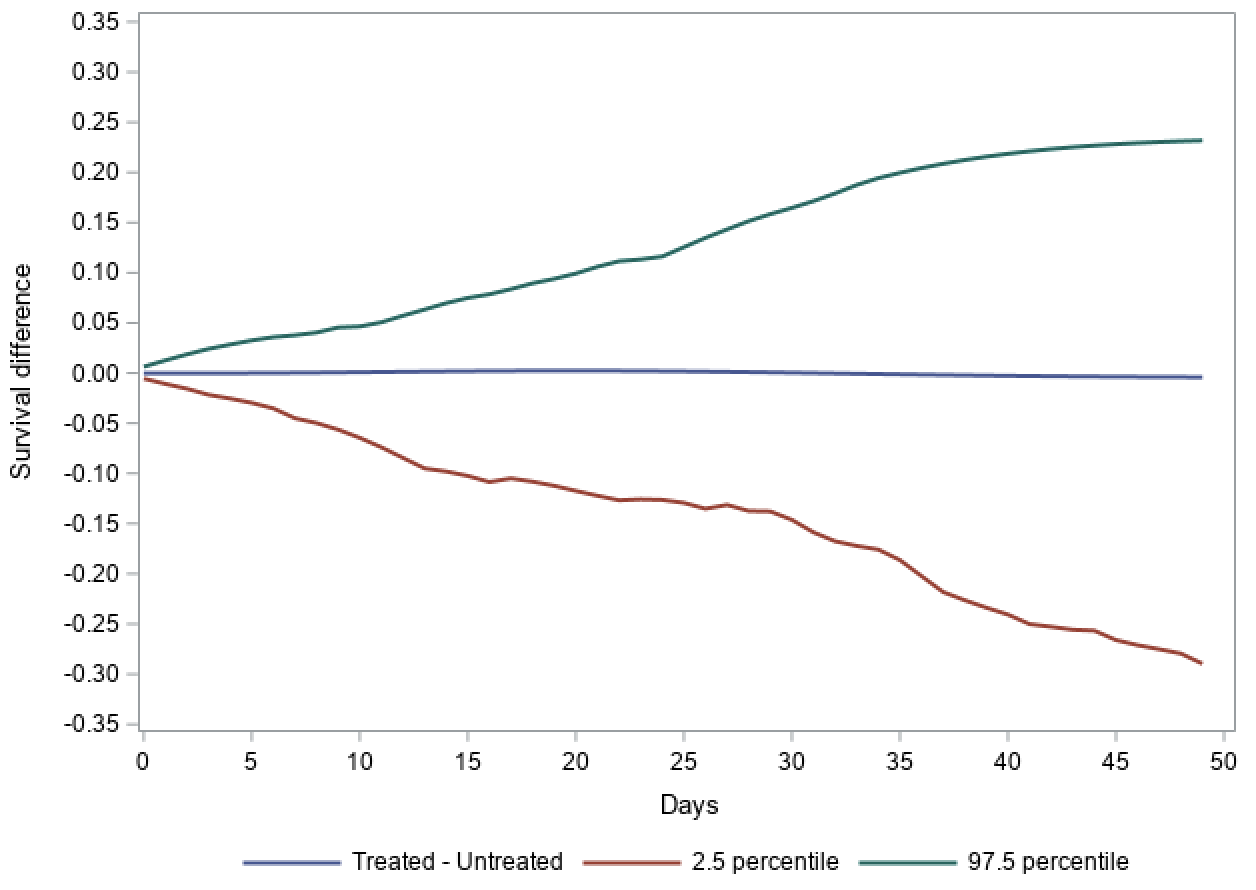 | D.  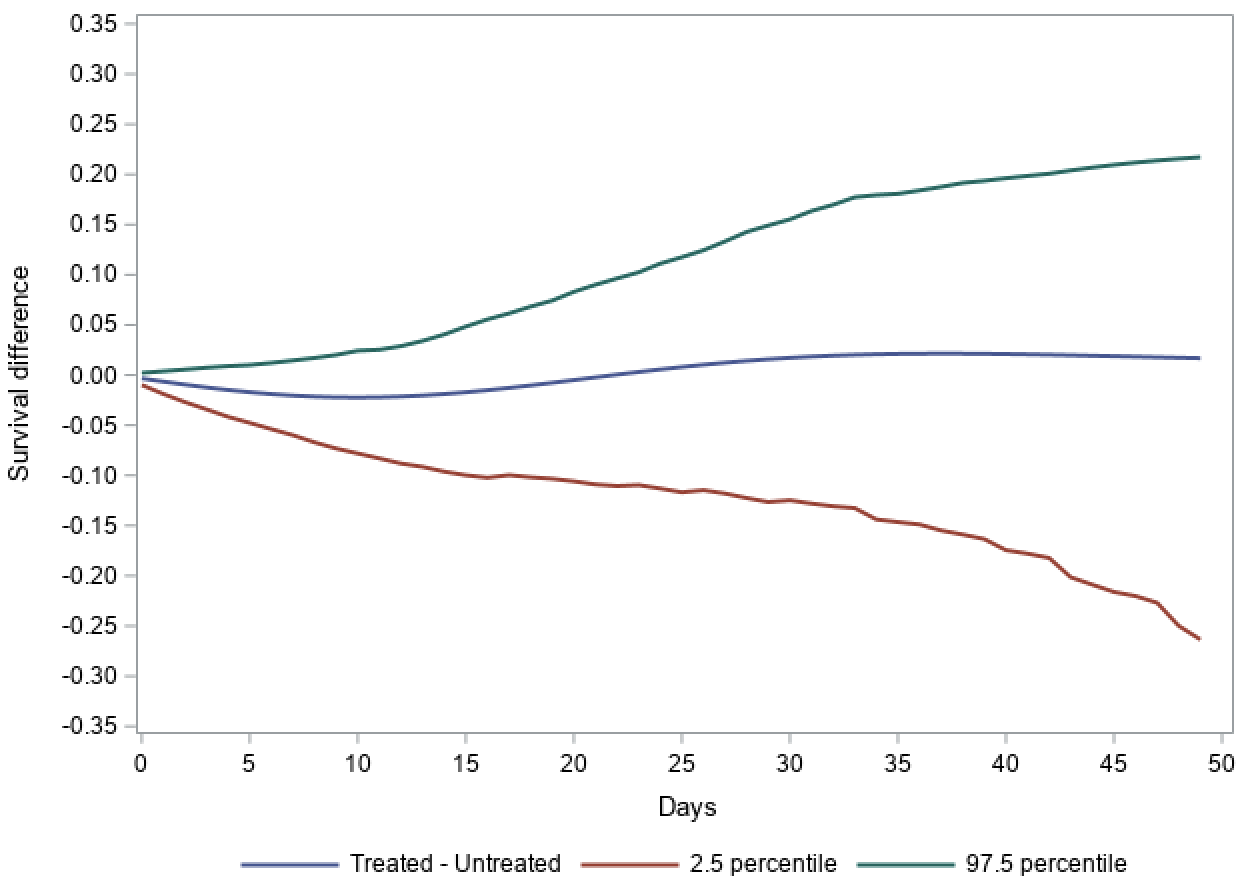 |
| A. In-hospital Survival up to 50 days among those met the septic shock definition;  B. In-hospital Survival up to 50 days among those did not meet the septic shock definition;  C. In-hospital Survival Difference between treatment groups among those met the septic shock definition;  D. In-hospital Survival Difference between treatment groups among those did not meet the septic shock definition; | |
