# Supplementary material for: Effectiveness of corticosteroids in patients with sepsis or septic shock using the new third international consensus definitions (Sepsis-3): A retrospective observational study
Source: PLoS One. 2020 Dec 3;15(12):e0243149. doi: 10.1371/journal.pone.0243149 (PMC7714118; doi:10.1371/journal.pone.0243149)
Supplement: S6 Fig — A. In-hospital Survival up to 50 days using 3-hour SOFA score assessment window; B. In-hospital Survival up to 50 days using 12-hour SOFA score assessment window; C. In-hospital Survival Difference using 3-hour SOFA score assessment window; D. In-hospital Survival Difference using 12-hour SOFA score assessment window; E. In-hospital Survival up to 50 days using 24-hour SOFA score assessment window; F. In-hospital Survival up to 50 days using 1-day exposure window; G. In-hospital Survival difference using 24-hour SOFA score assessment window; H. In-hospital Survival difference using 1-day exposure window; I. In-hospital Survival up to 50 days using 5-day SOFA score assessment window; J. In-hospital Survival up to 50 days using Corticosteroid daily dose 200 to 400mg; K. In-hospital Survival difference using 5-day SOFA score assessment window; L. In-hospital Survival difference using Corticosteroid daily dose 200 to 400mg; M. In-hospital Survival up to 50 days when individuals received no any corticosteroids as controls; N. In-hospital Survival up to 50 days when excluding patients with asthma or COPD; O. In-hospital Survival difference when individuals received no any corticosteroids as controls; P. In-hospital Survival difference when excluding patients with asthma or COPD; Q. In-hospital Survival up to 50 days when excluding hospitals that never prescribed corticosteroids; R. In-hospital Survival up to 50 days when using g-formula; S. In-hospital Survival difference when excluding hospitals that never prescribed corticosteroids; T. In-hospital Survival difference when using g-formula; U. In-hospital Survival up to 50 days using any dose of corticosteroids as controls; V. In-hospital Survival up to 50 days when excluding >1 hospitalization due to sepsis or septic shock within 2014–2015; W. In-hospital Survival difference using any dose of corticosteroids as controls; X. In-hospital Survival difference when excluding >1 hospitalization due to sepsis or septic shock wi [file pone.0243149.s021.docx]

S6 Fig. In-hospital Survival and Survival Difference between Treated and Non-Treated from the Sensitivity Analyses in the Sepsis-3 Cohort

| A.  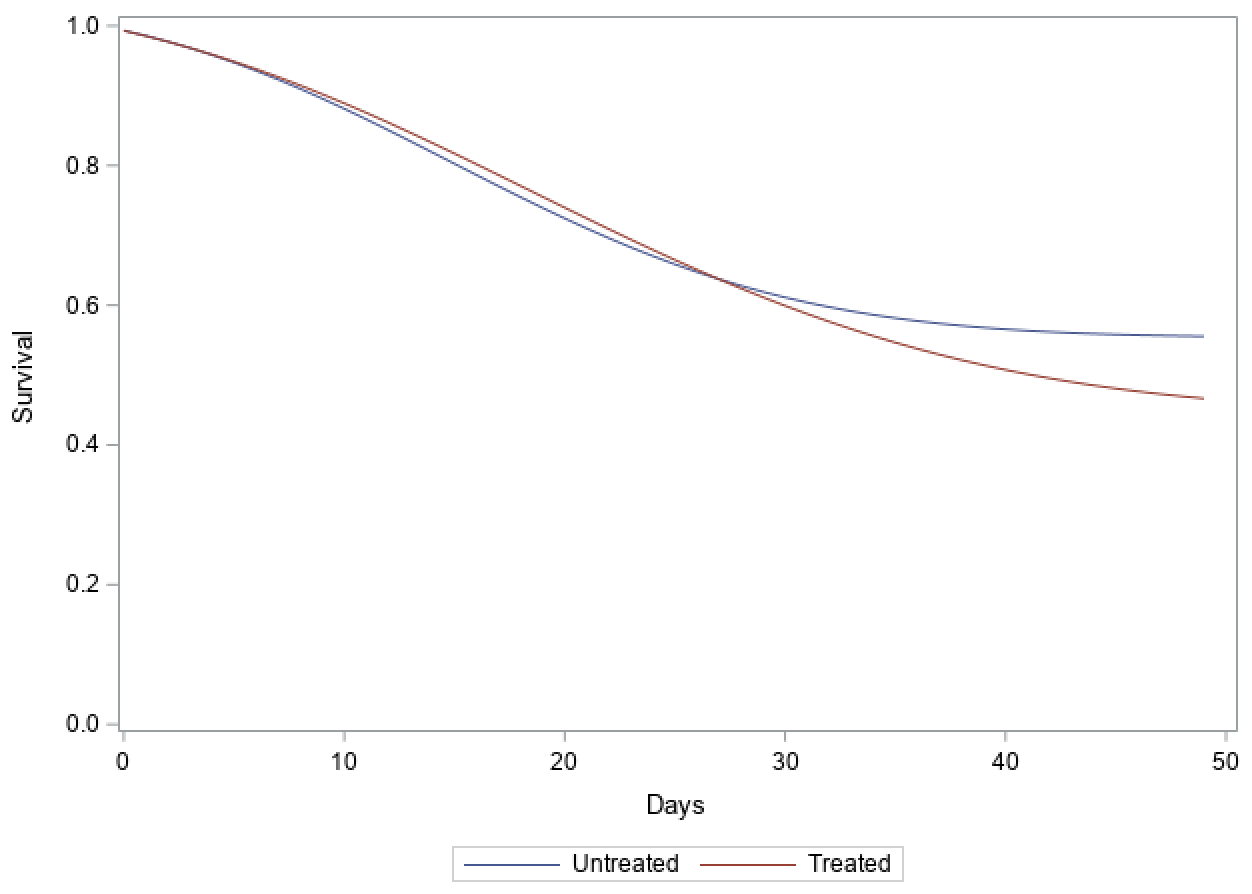 | B.  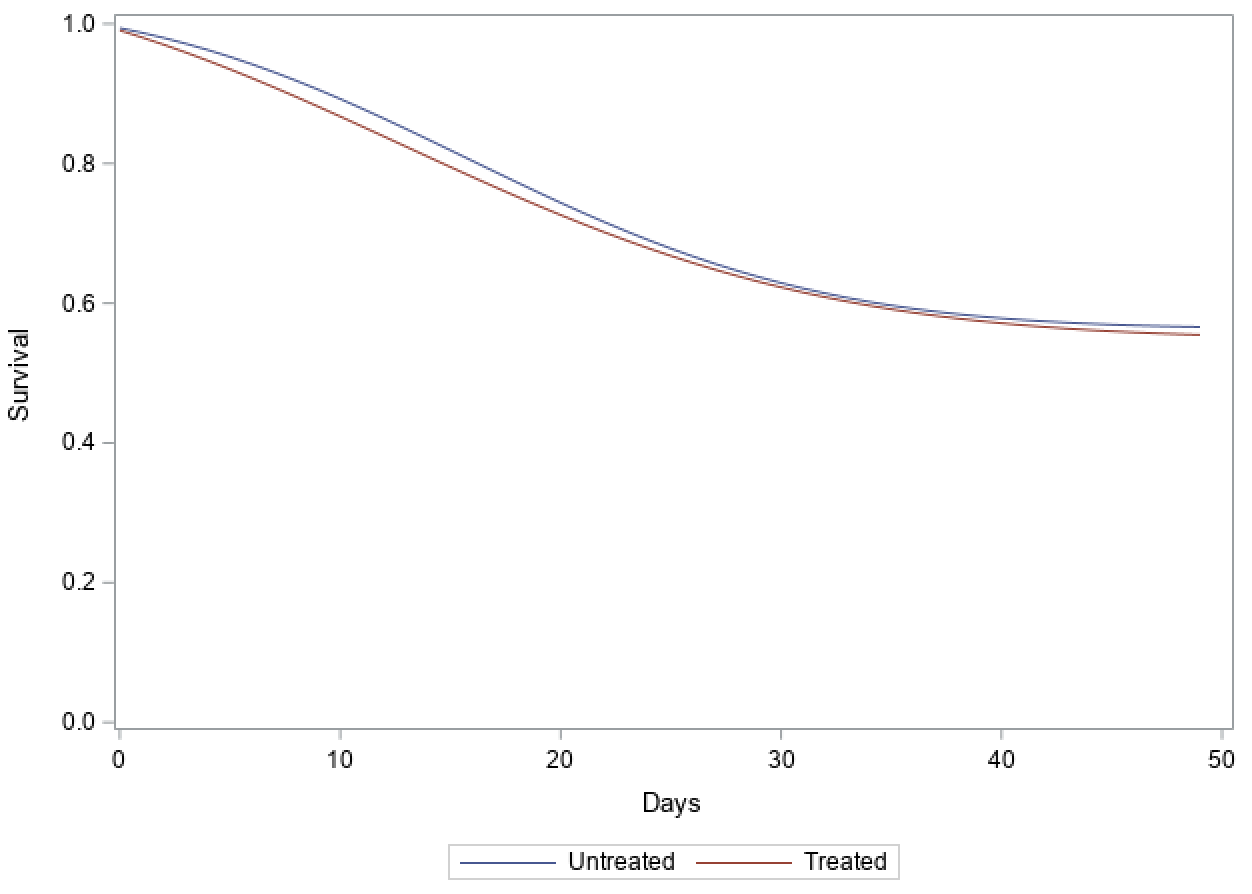 |
| --- | --- |
| C.  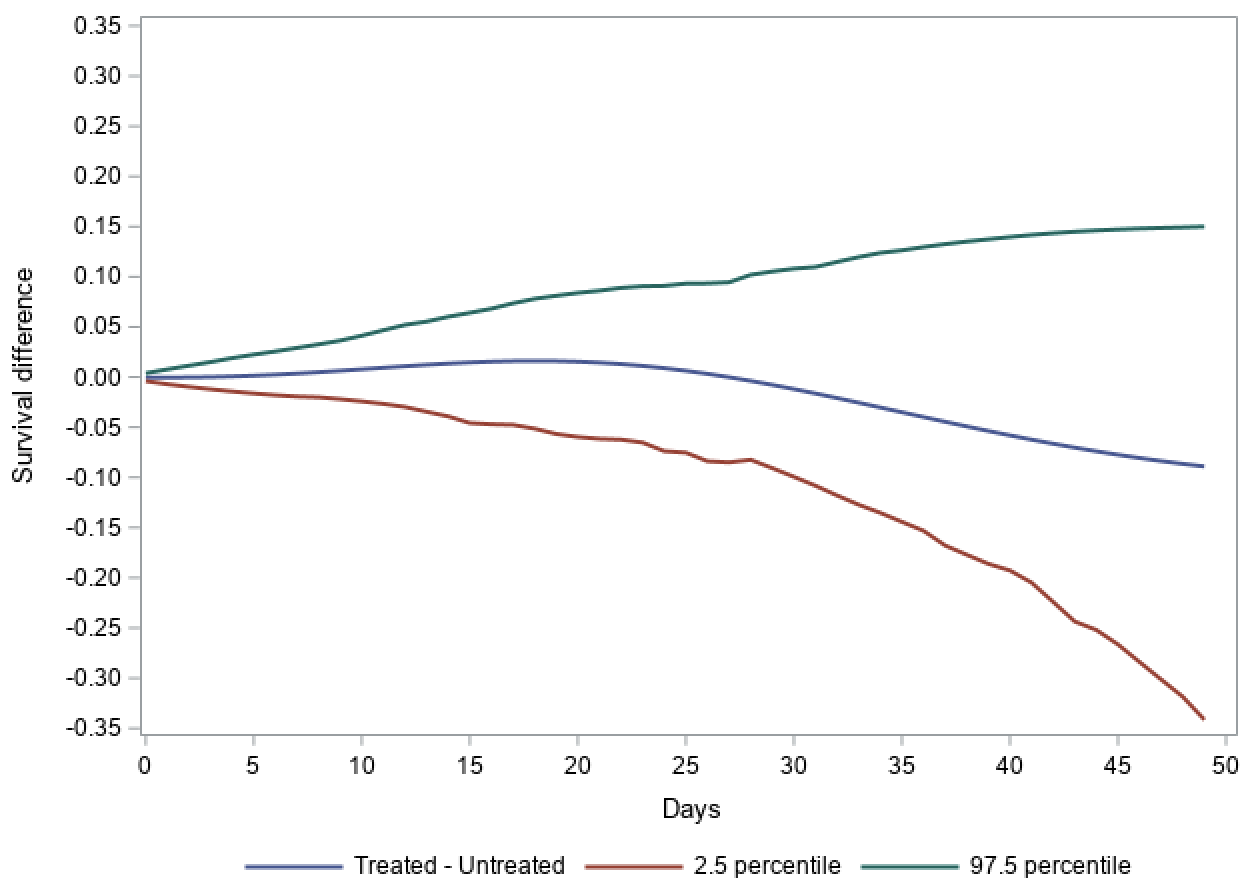 | D.  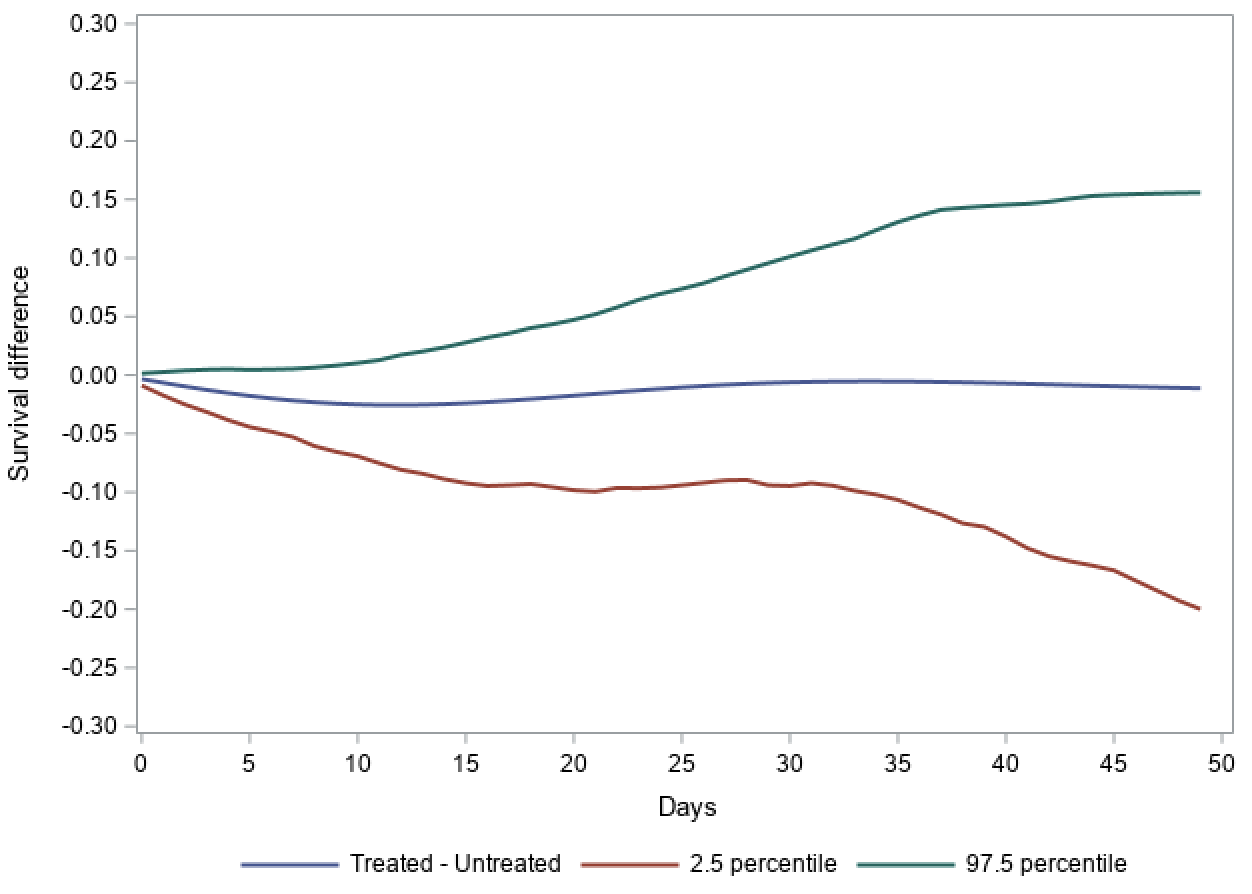 |
|  |  |
| E.  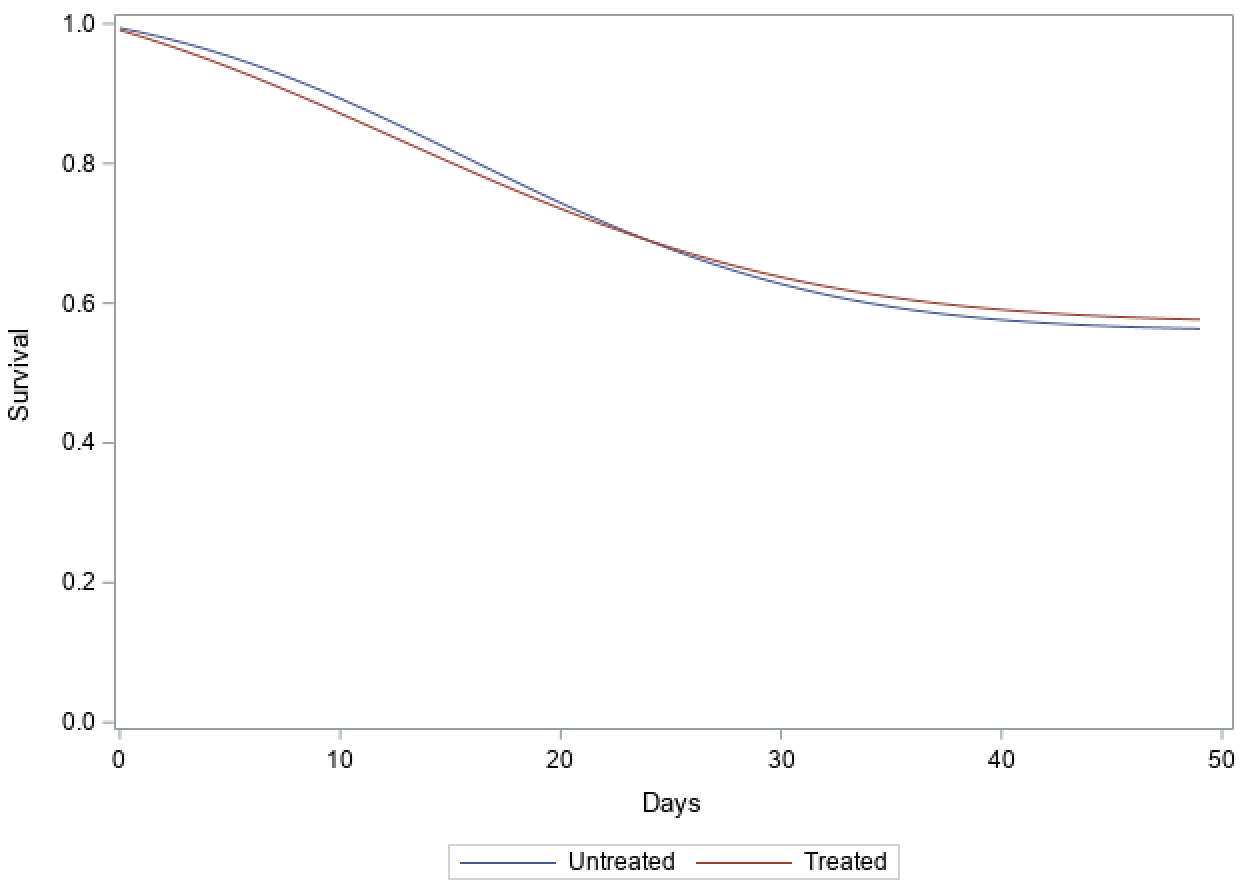 | F.  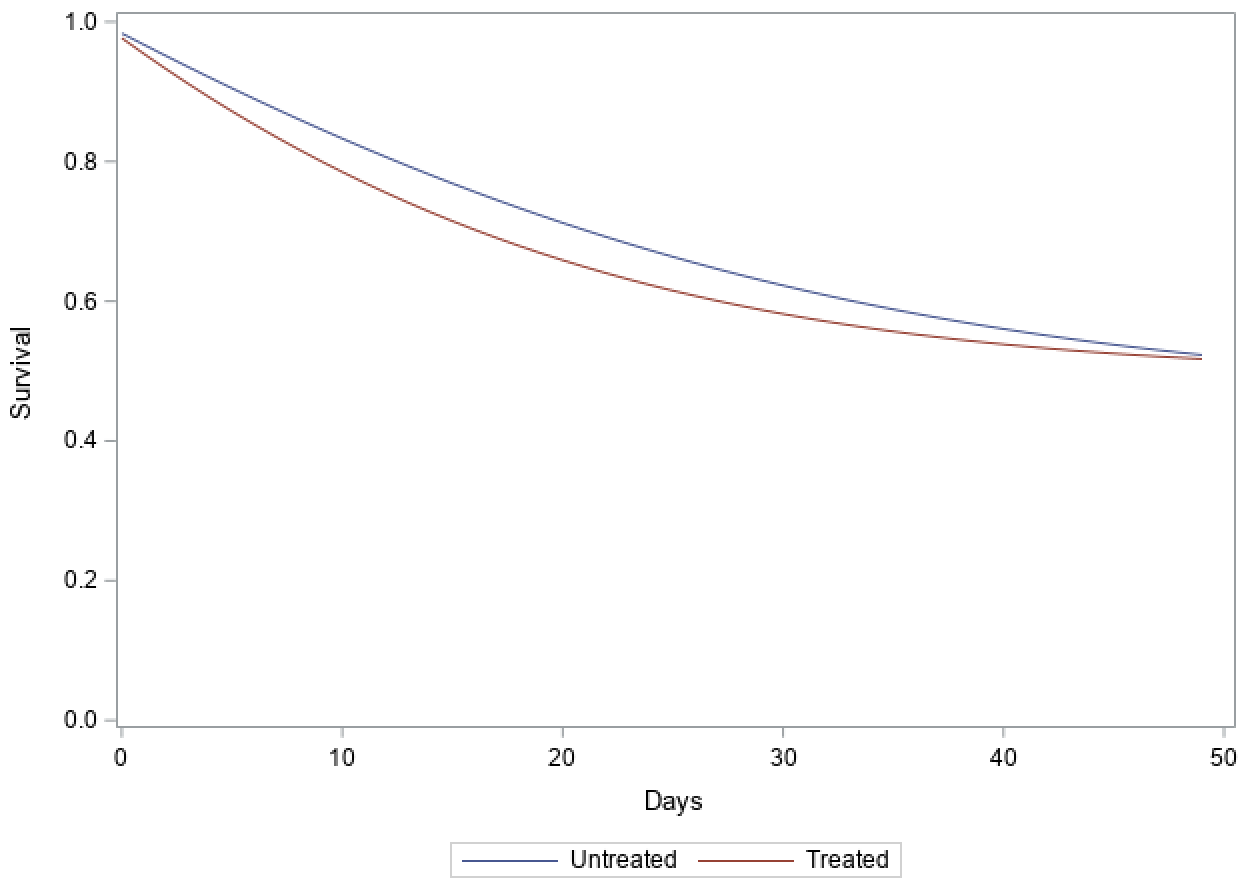 |
| G.  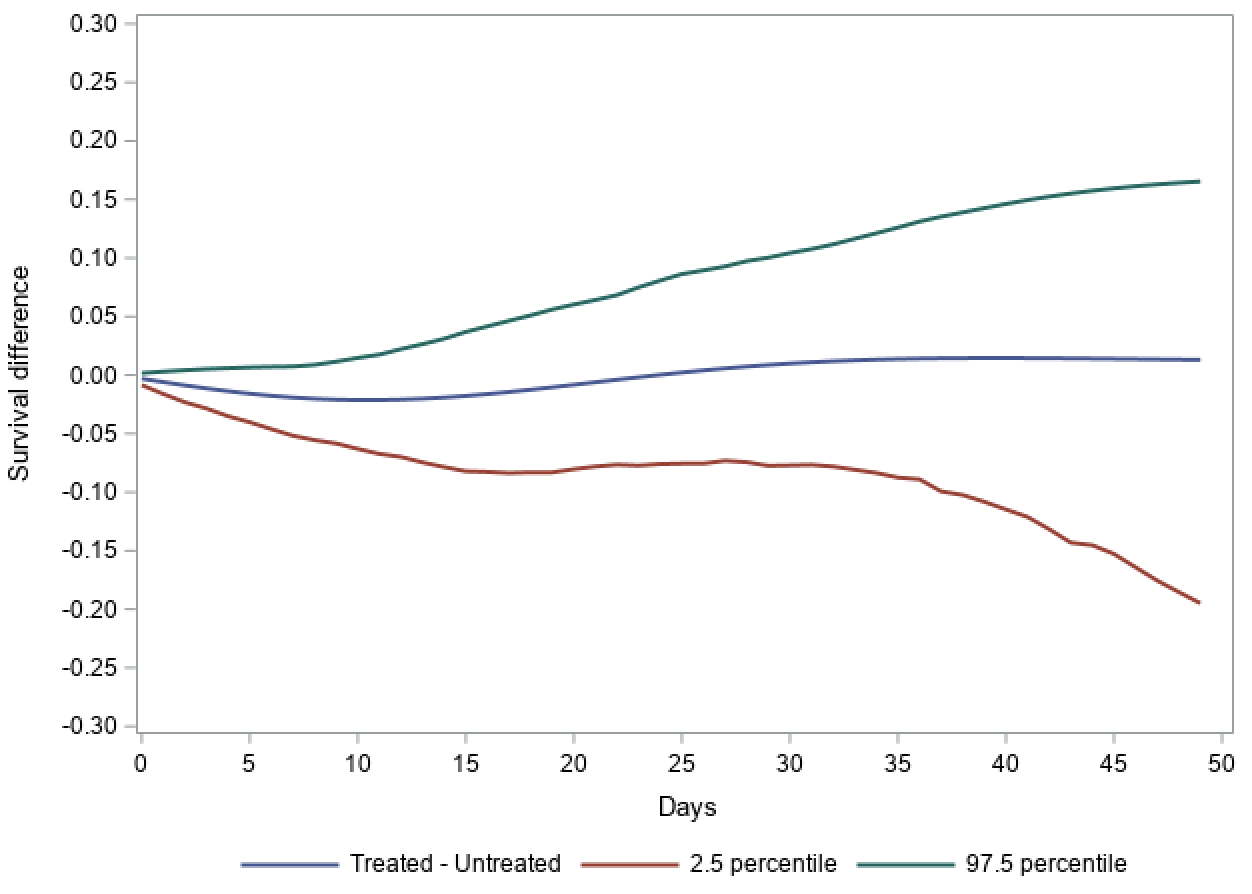 | H.  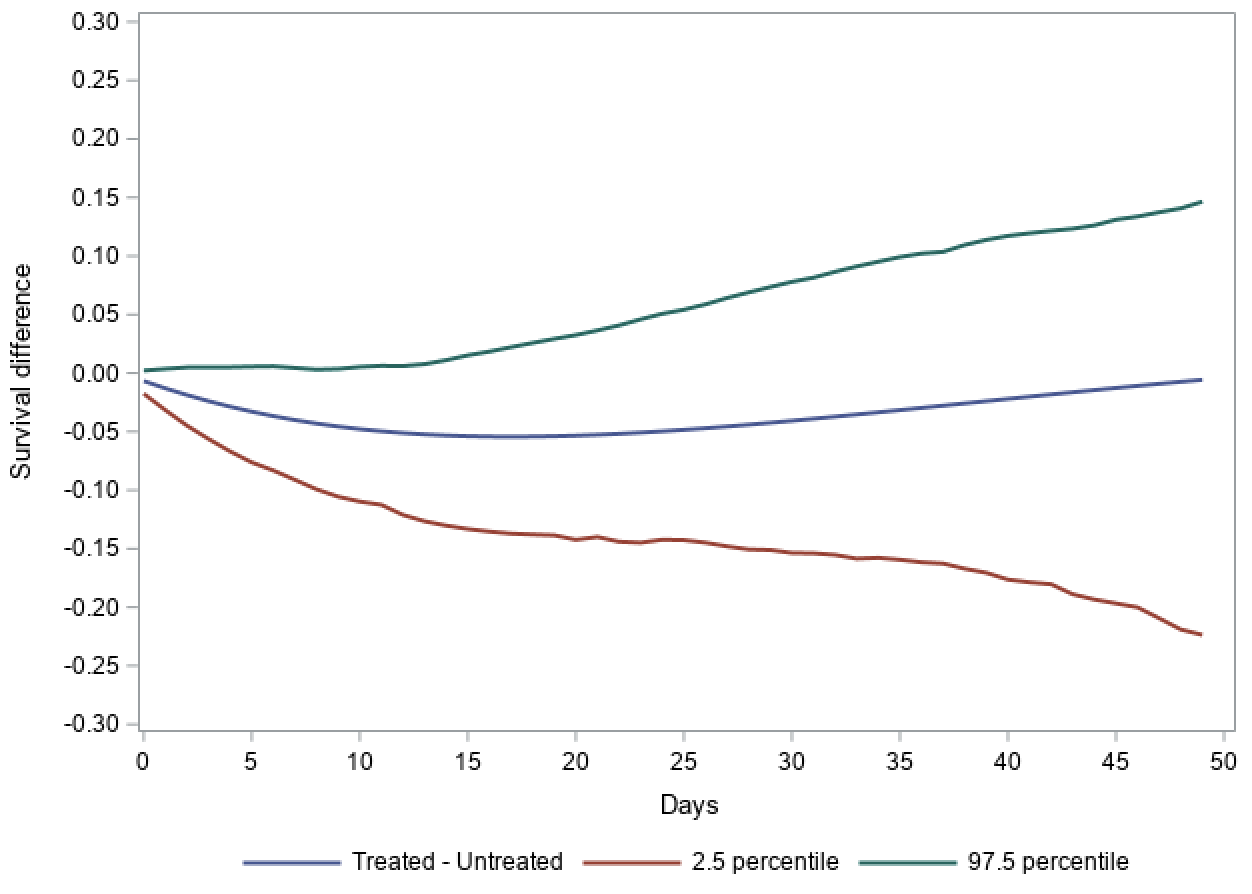 |
|  |  |
| I.  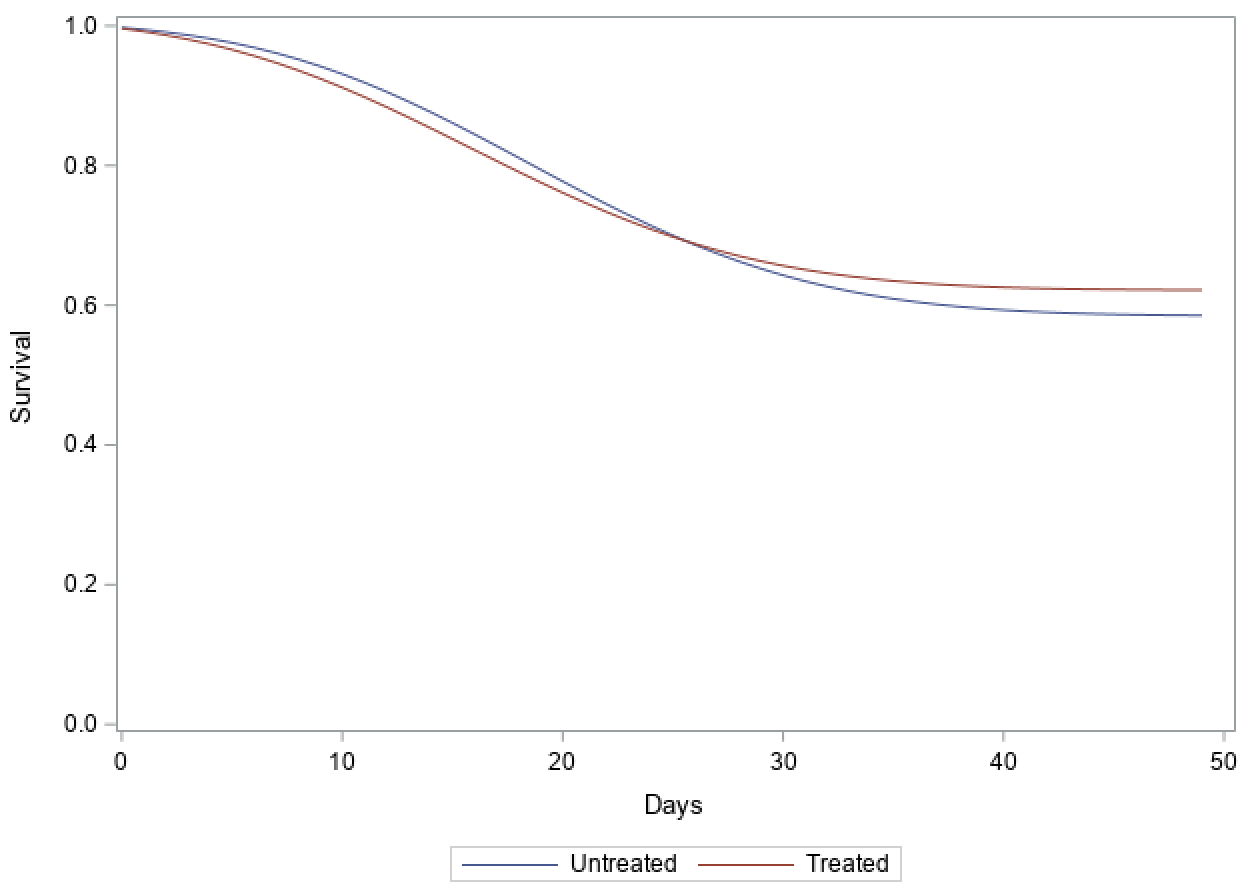 | J.  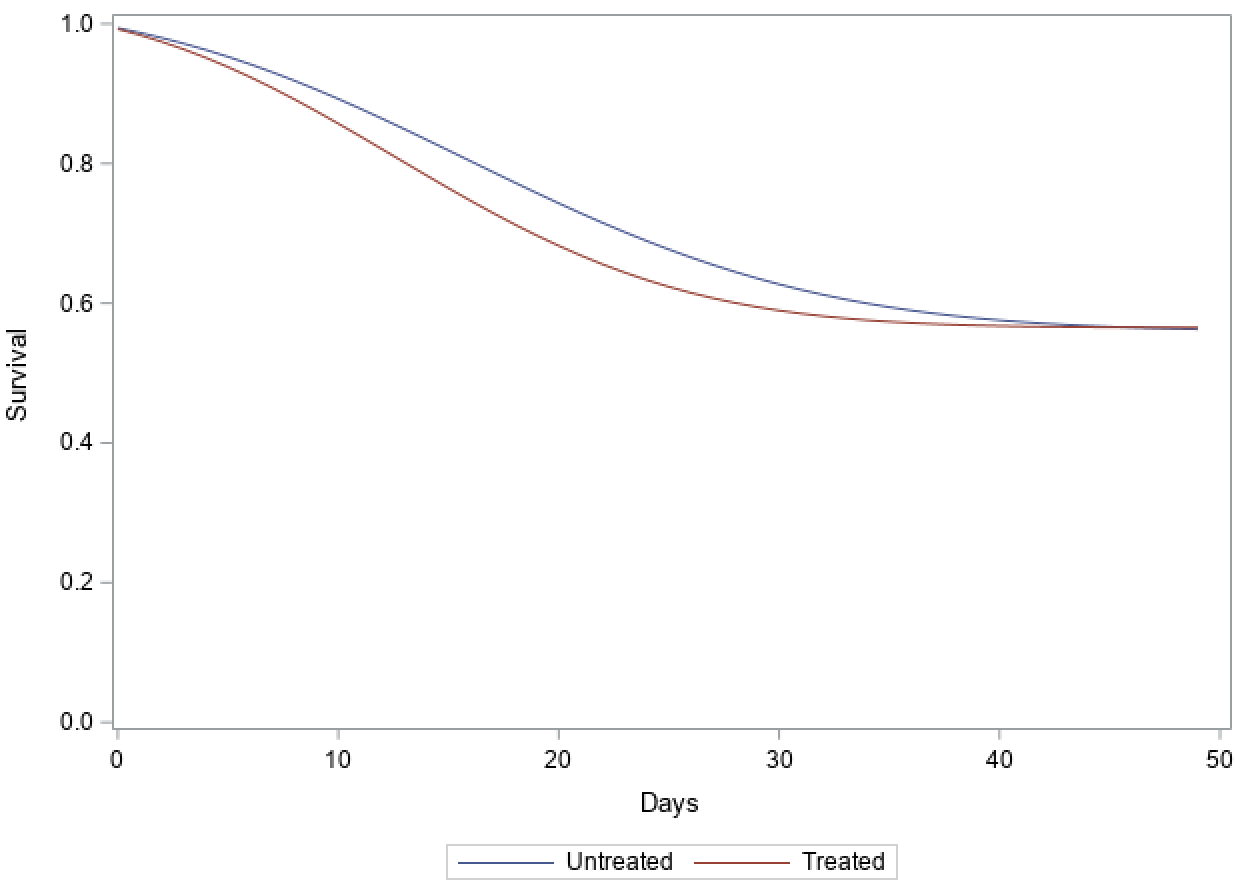 |
| K.  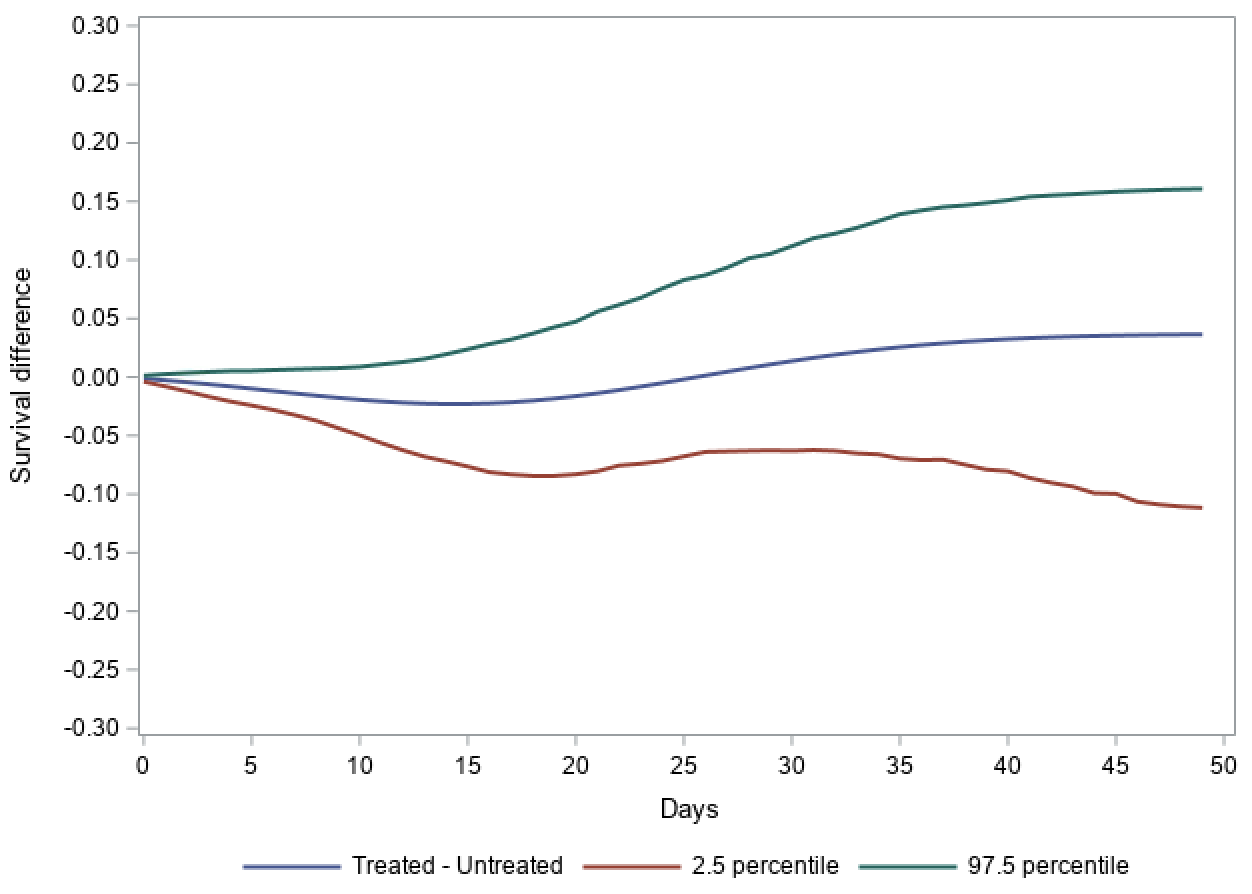 | L.  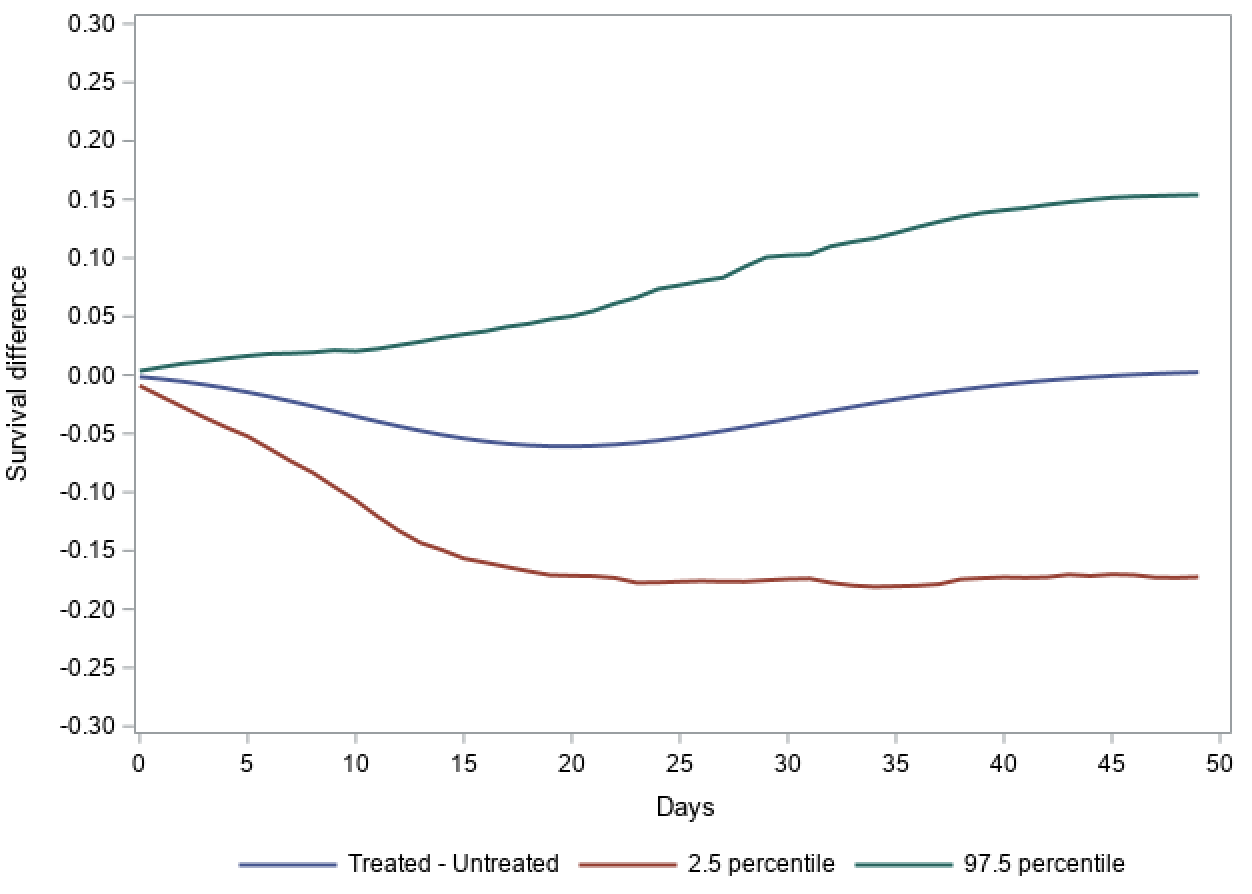 |
|  |  |
| M.  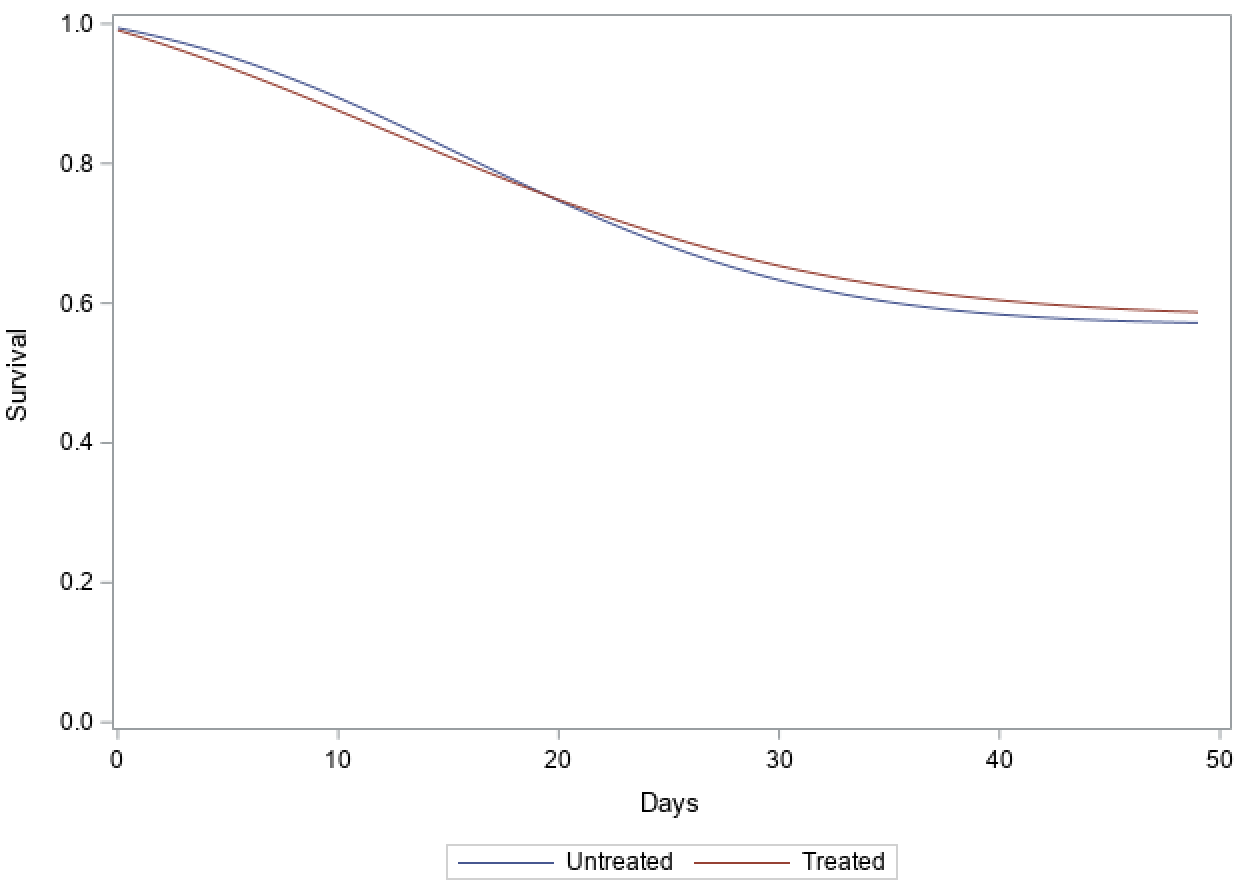 | N.  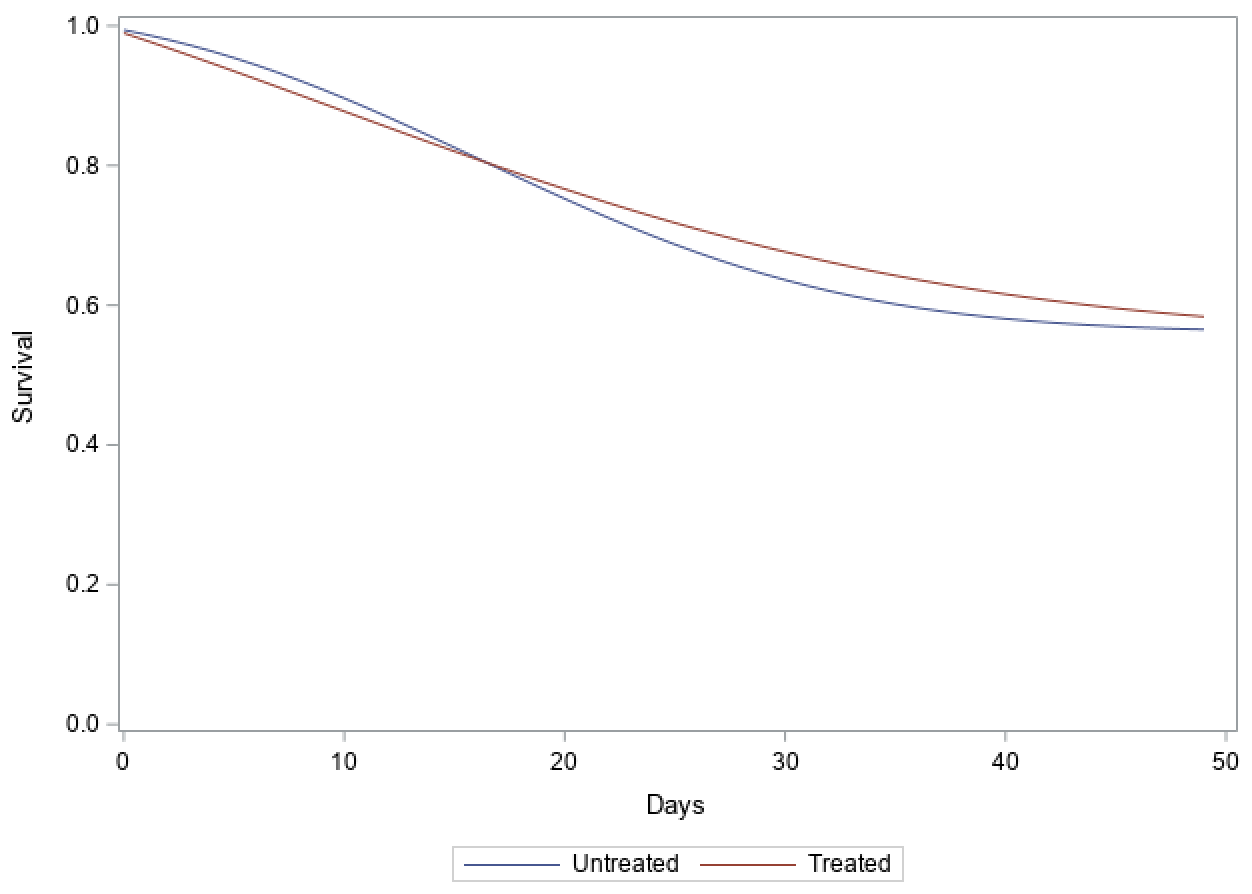 |
| O.  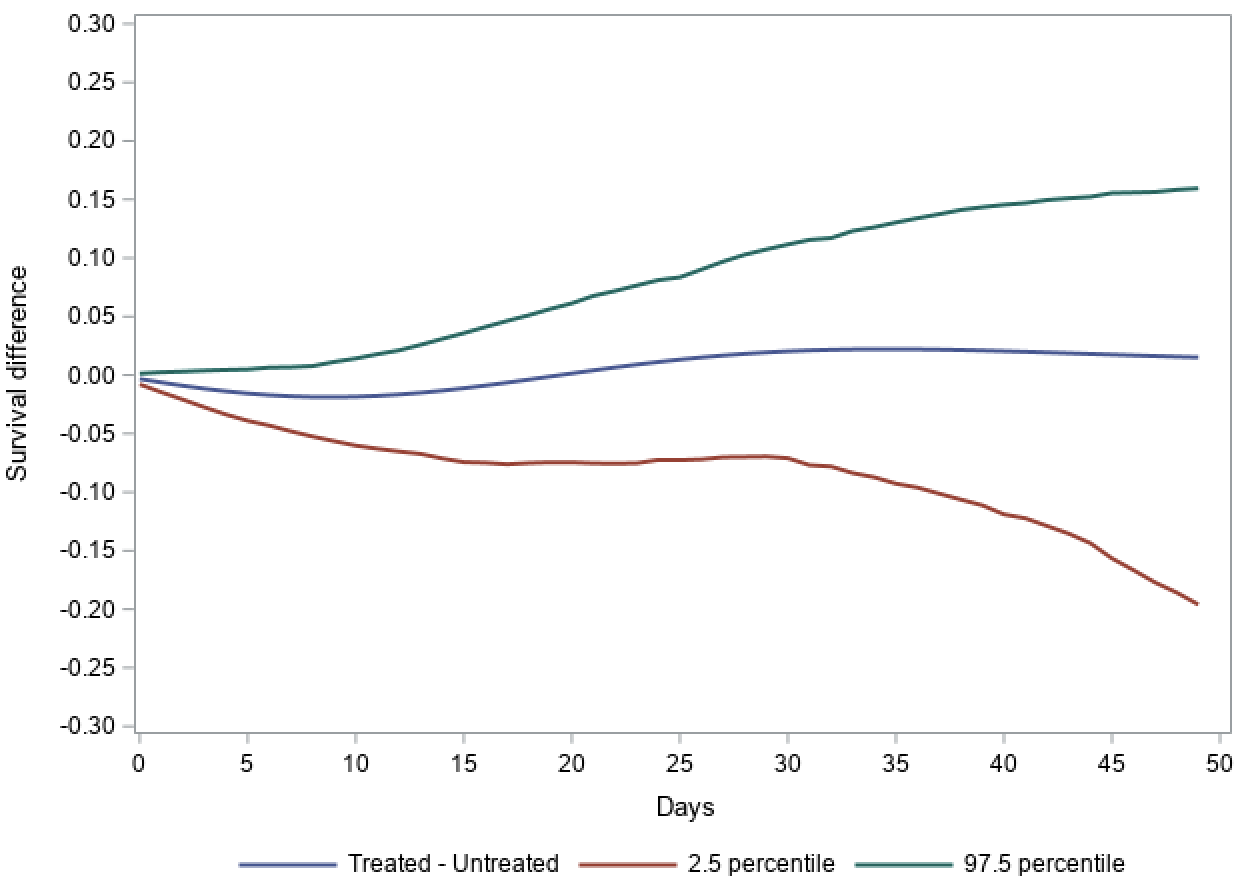 | P.  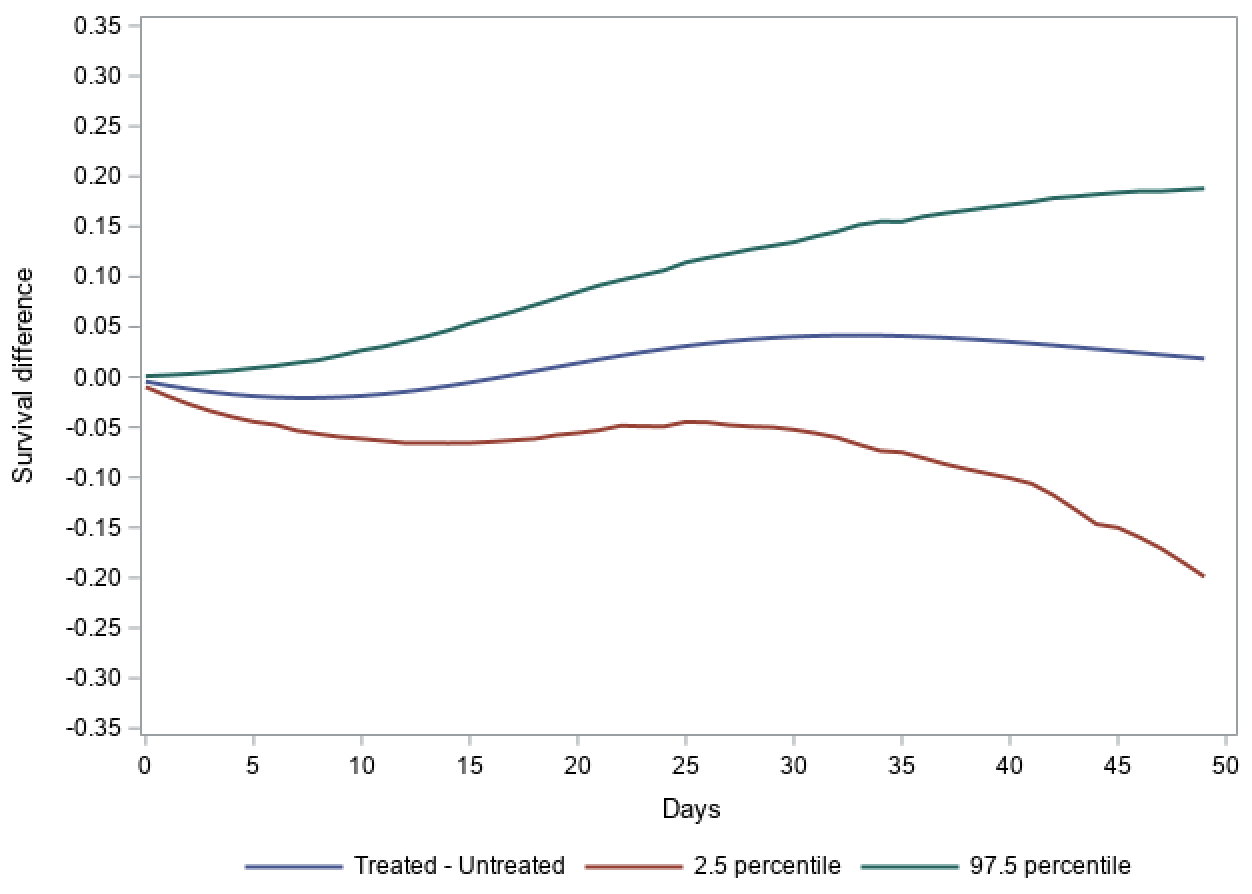 |
| Q.  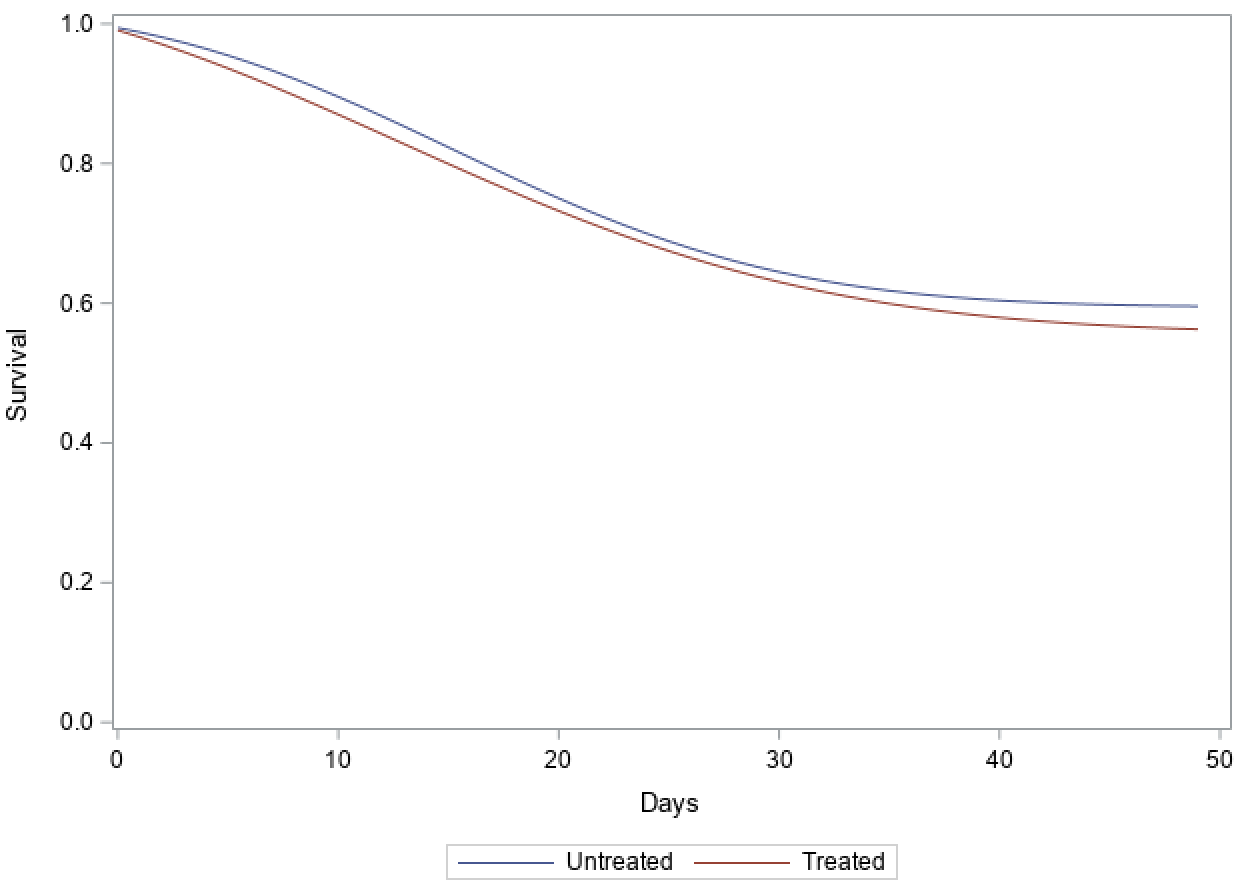 | R.  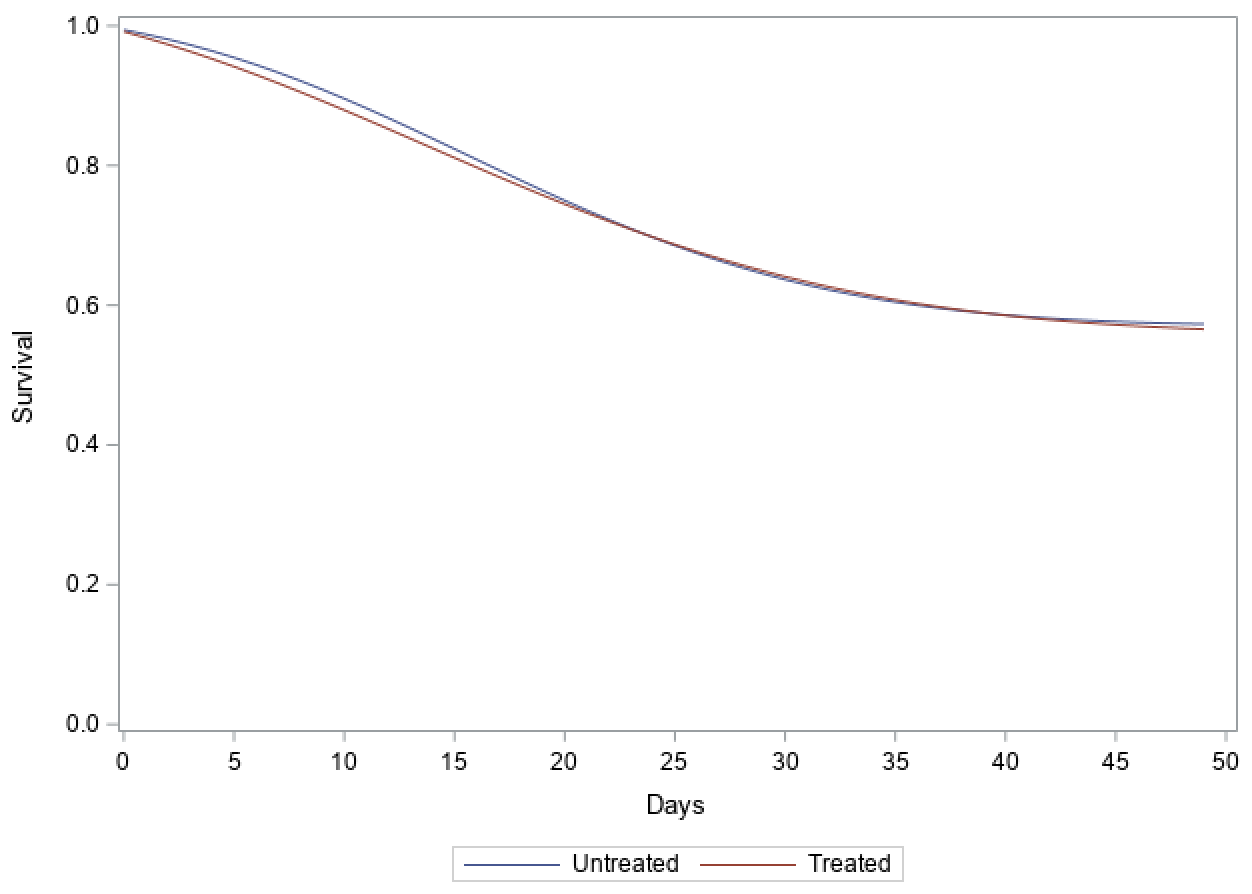 |
| S.  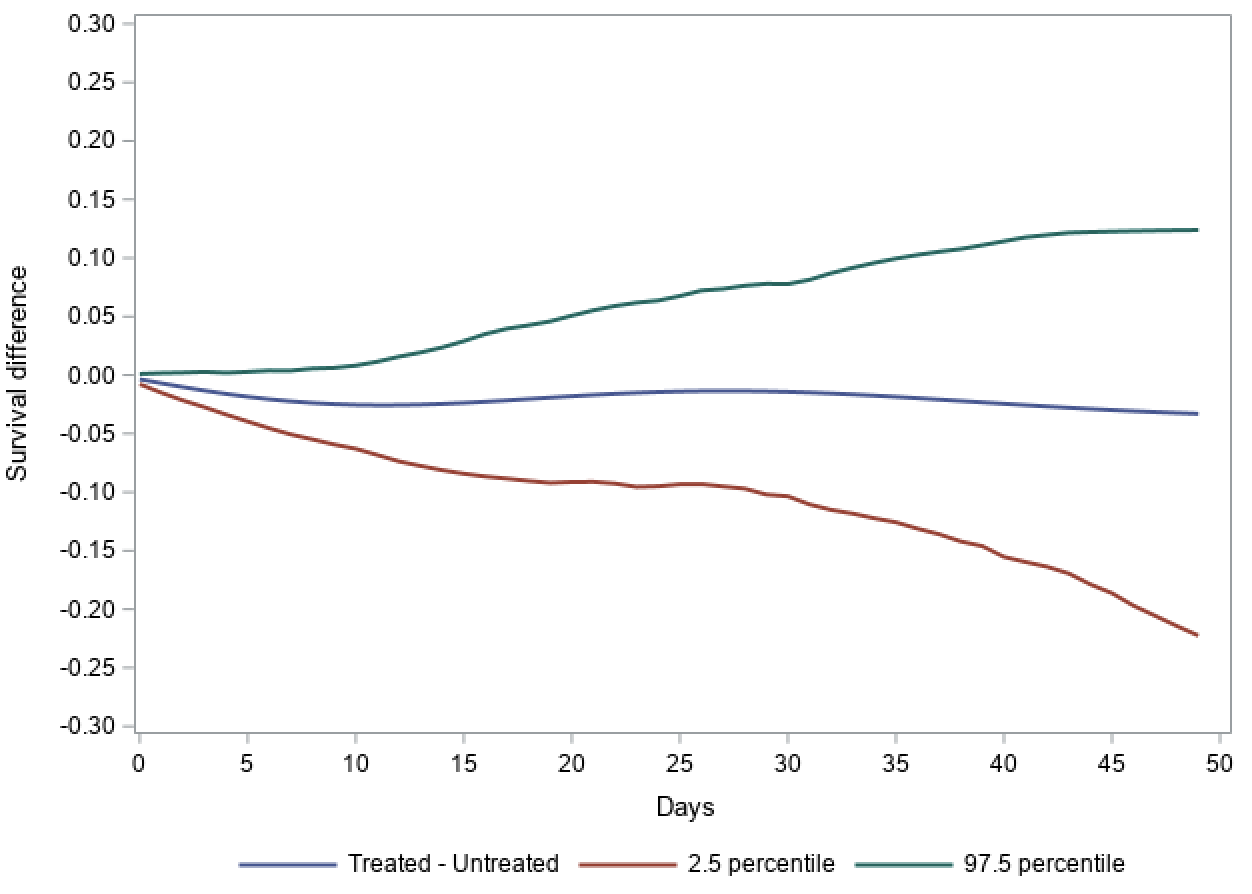 | T.  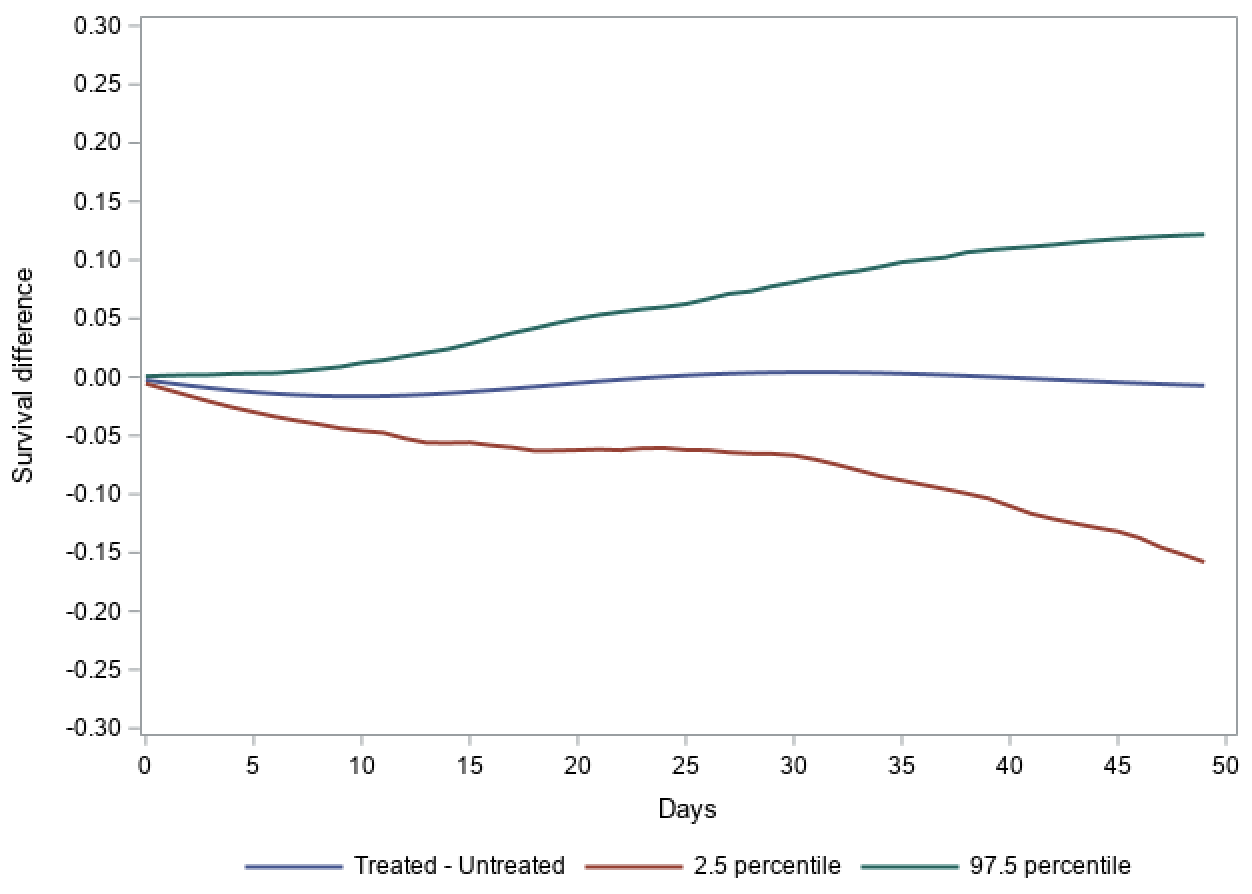 |
| U.  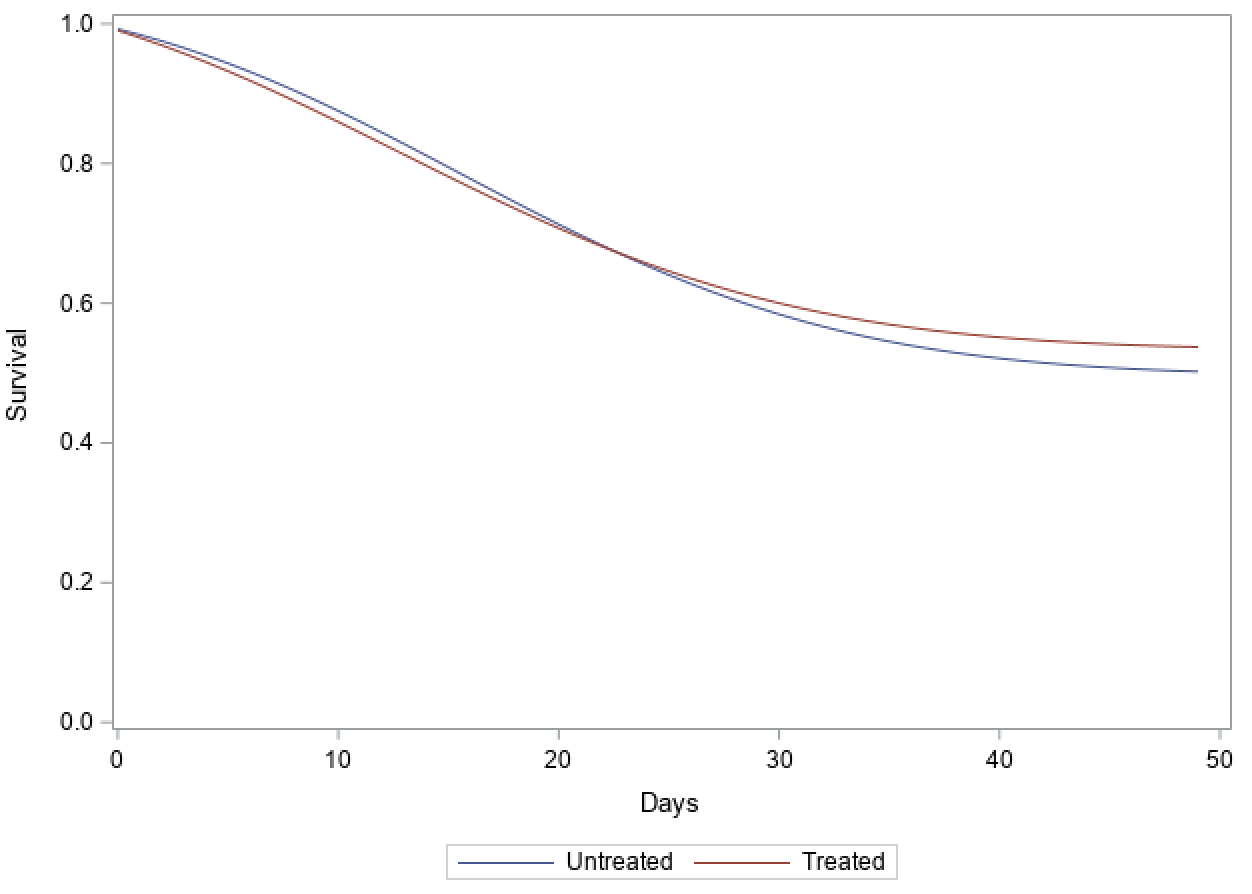 | V.  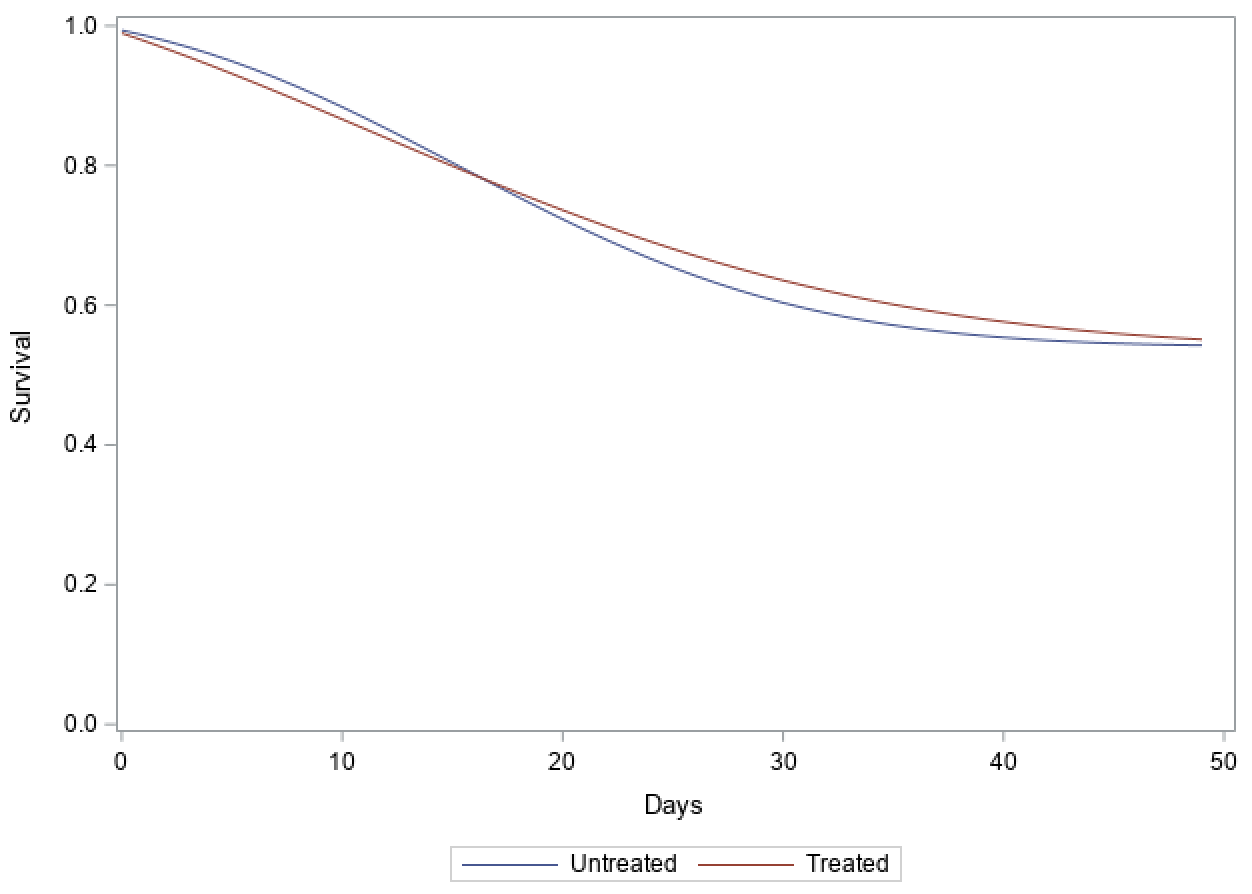 |
| W.  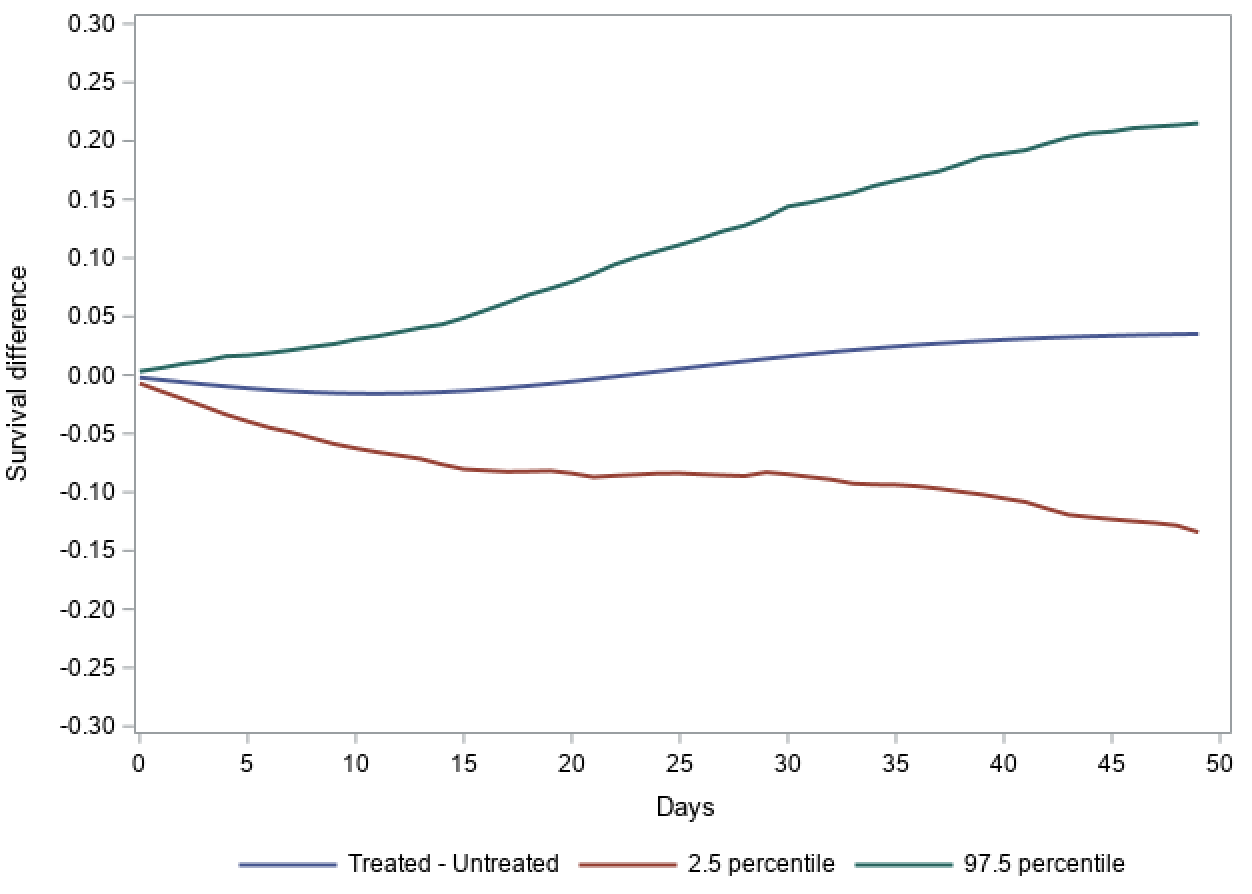 | X.  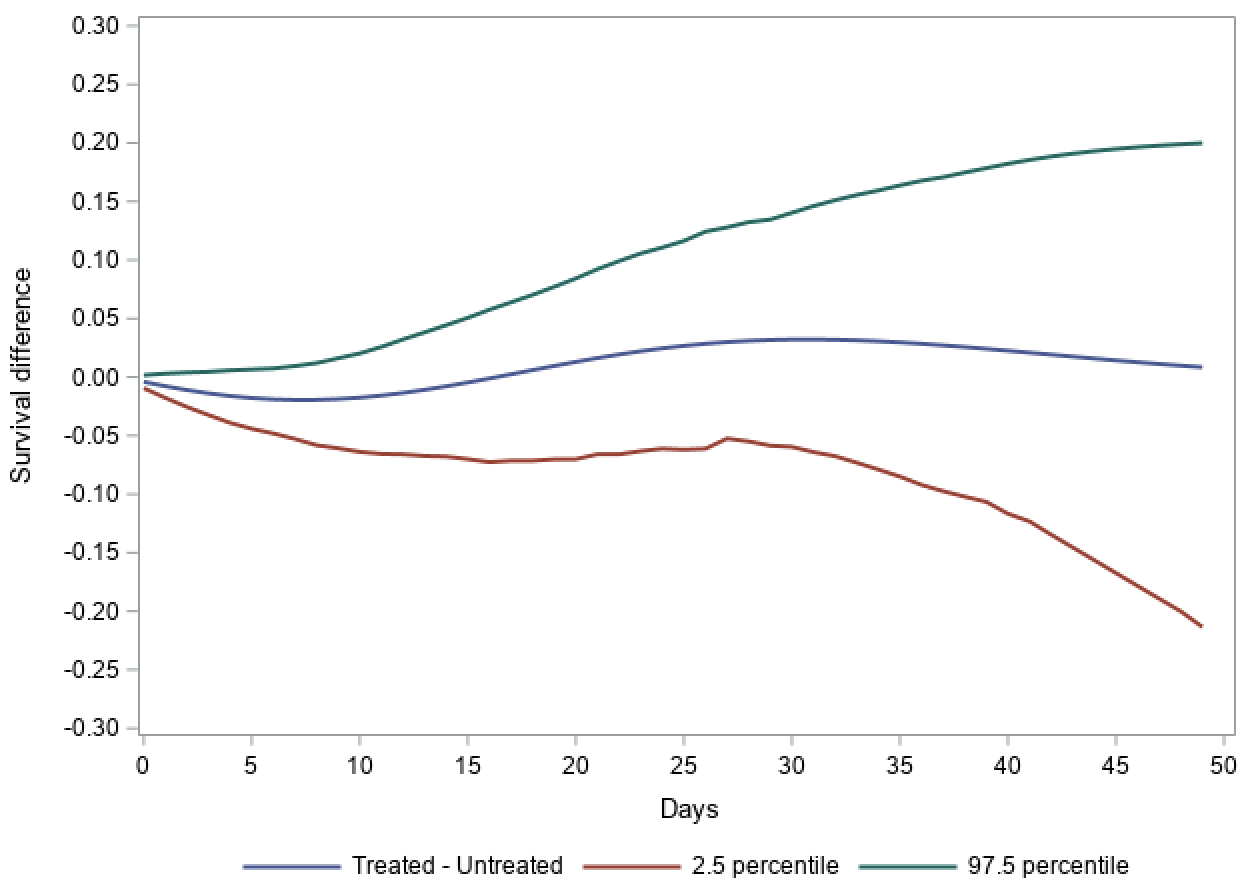 |
| Y.  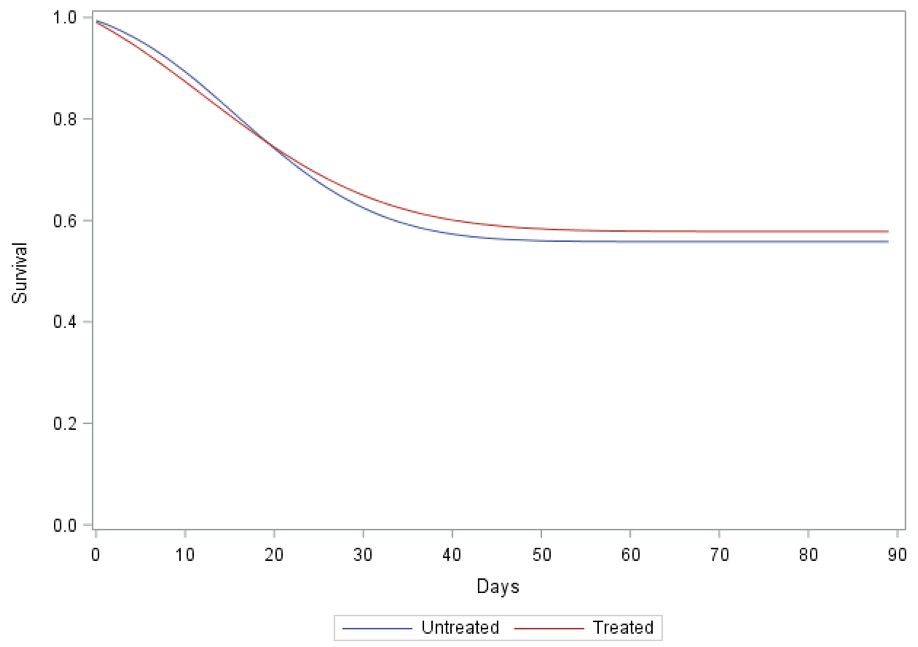 | Z.  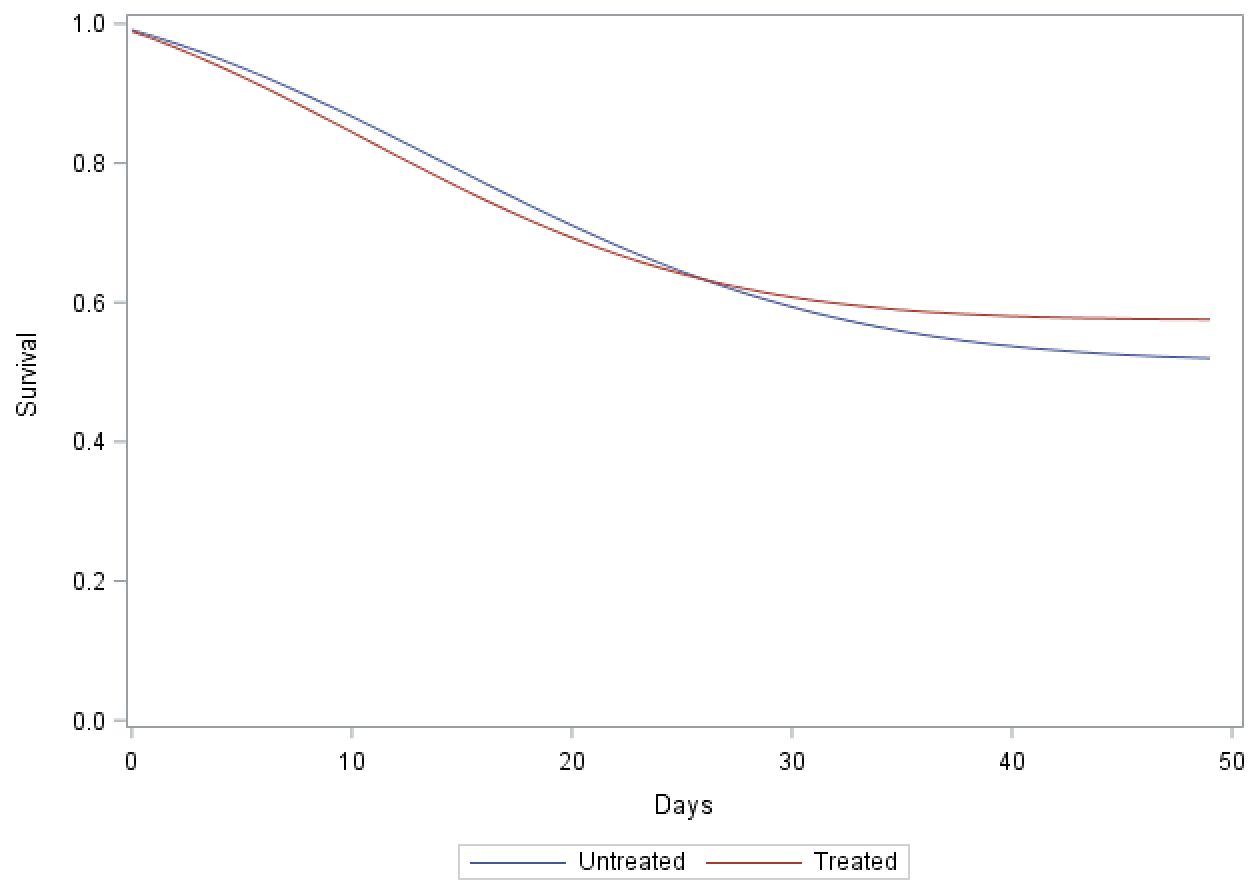 |
| AA.  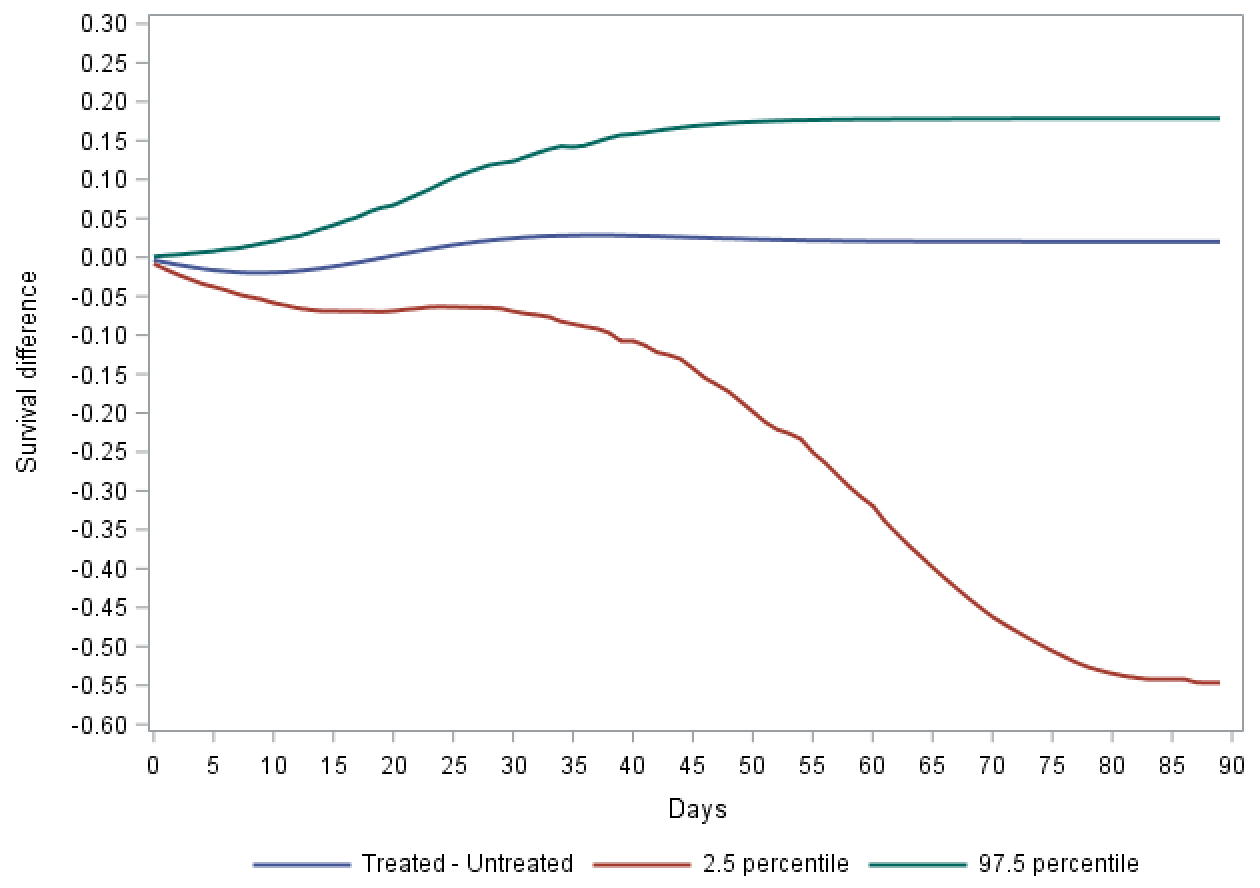 | AB.  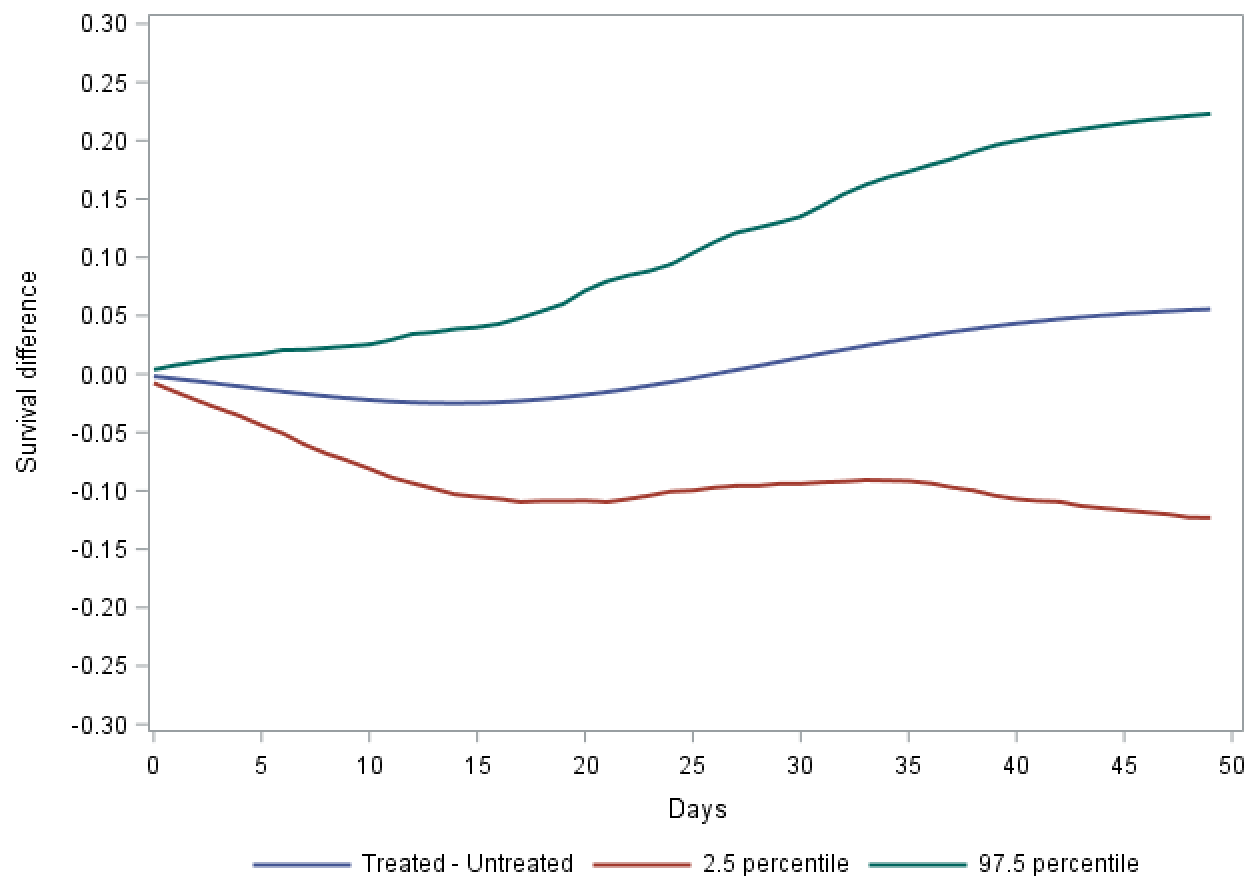 |
| AC.  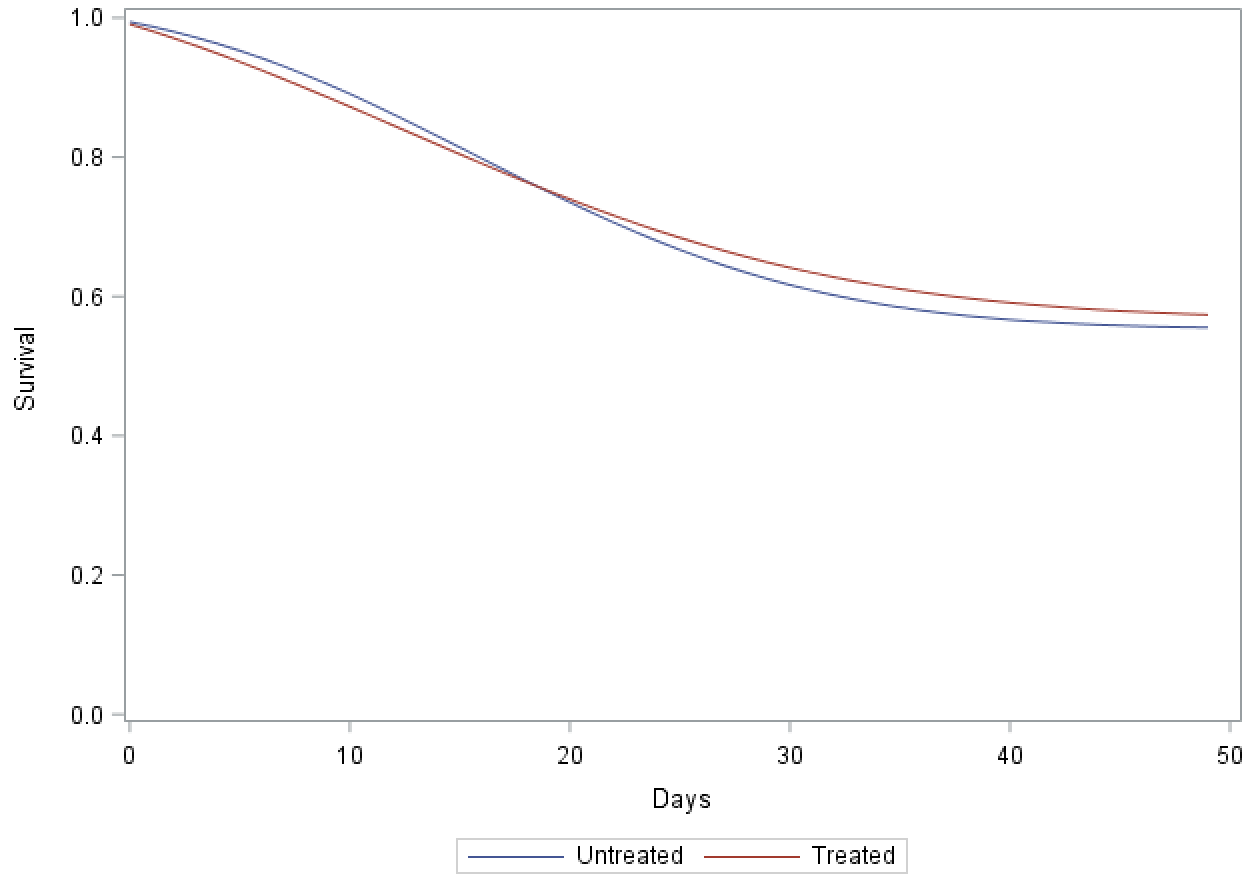 | AD.  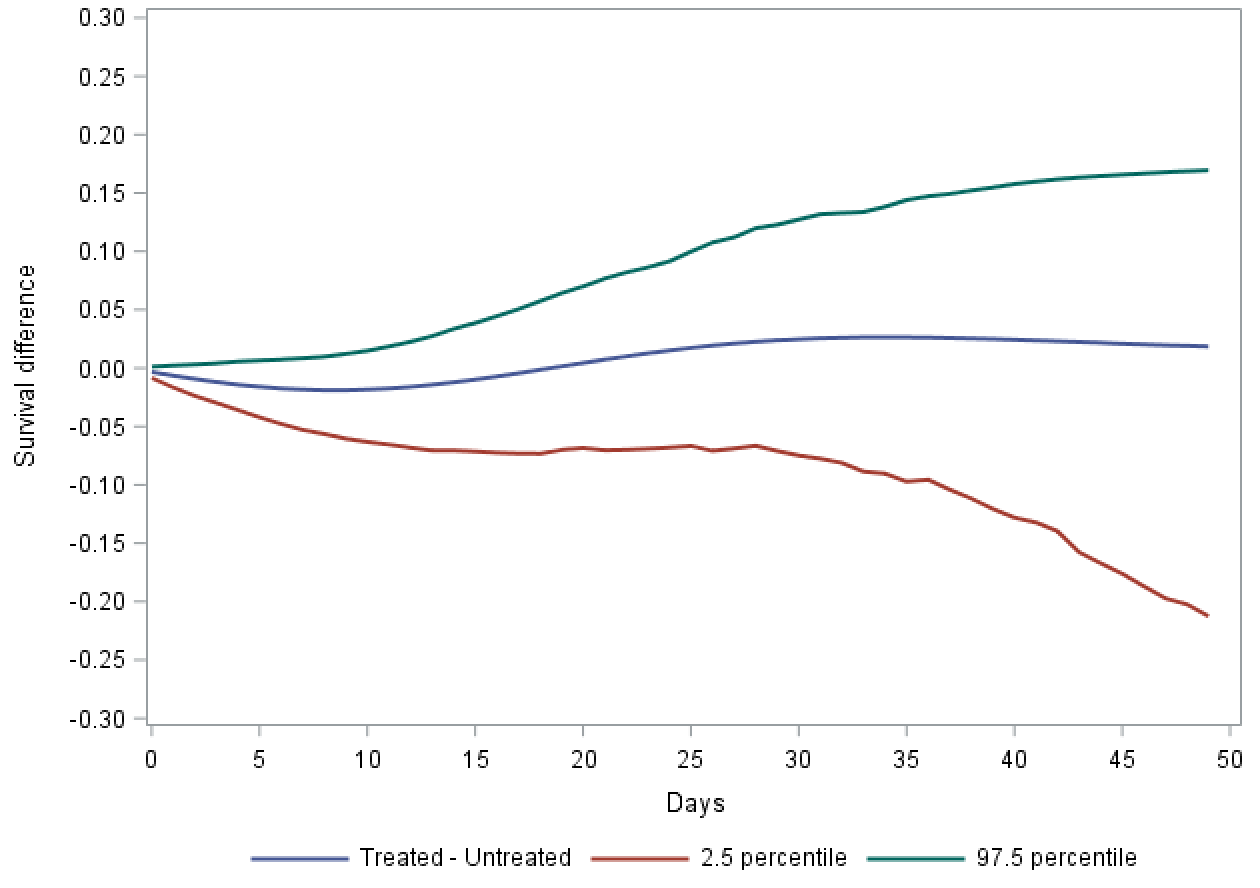 |
| AE.  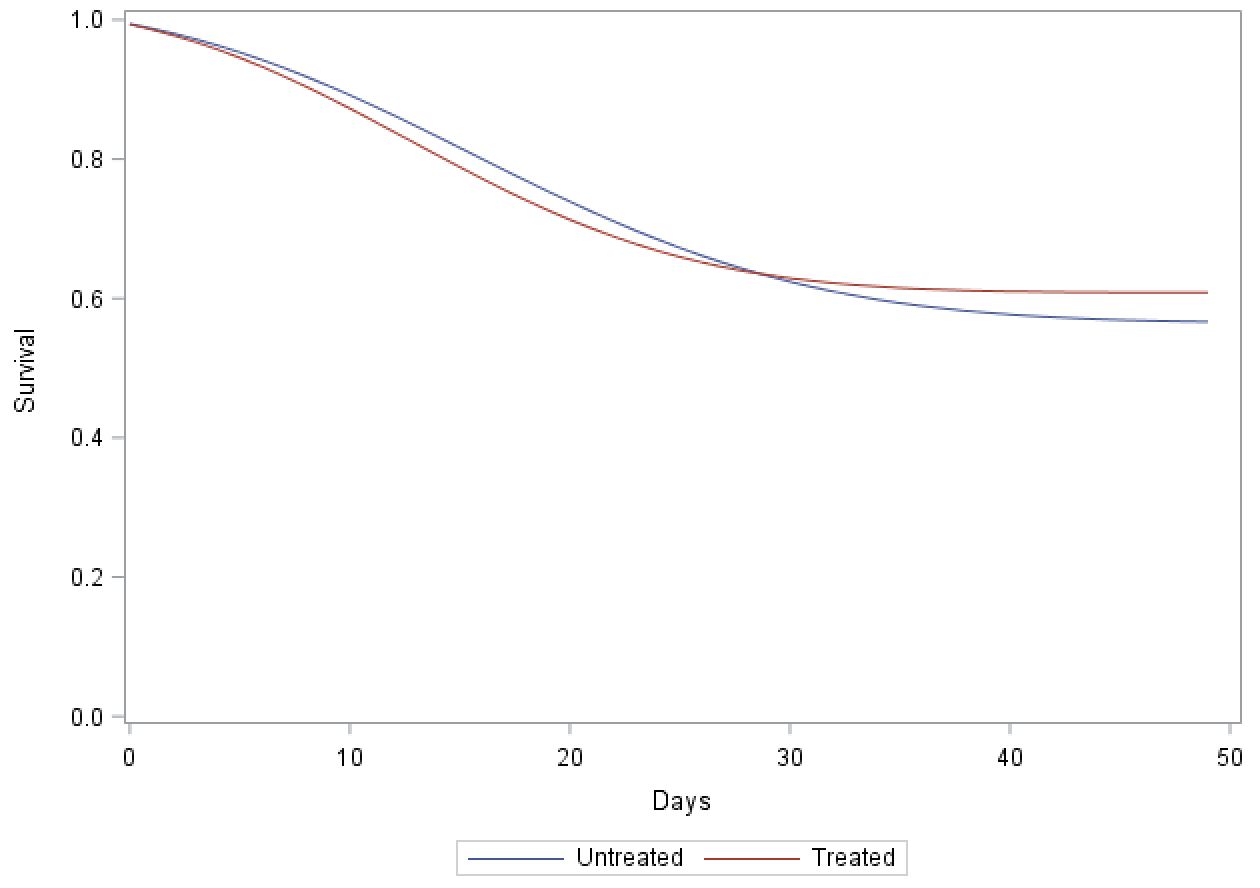 | AF.  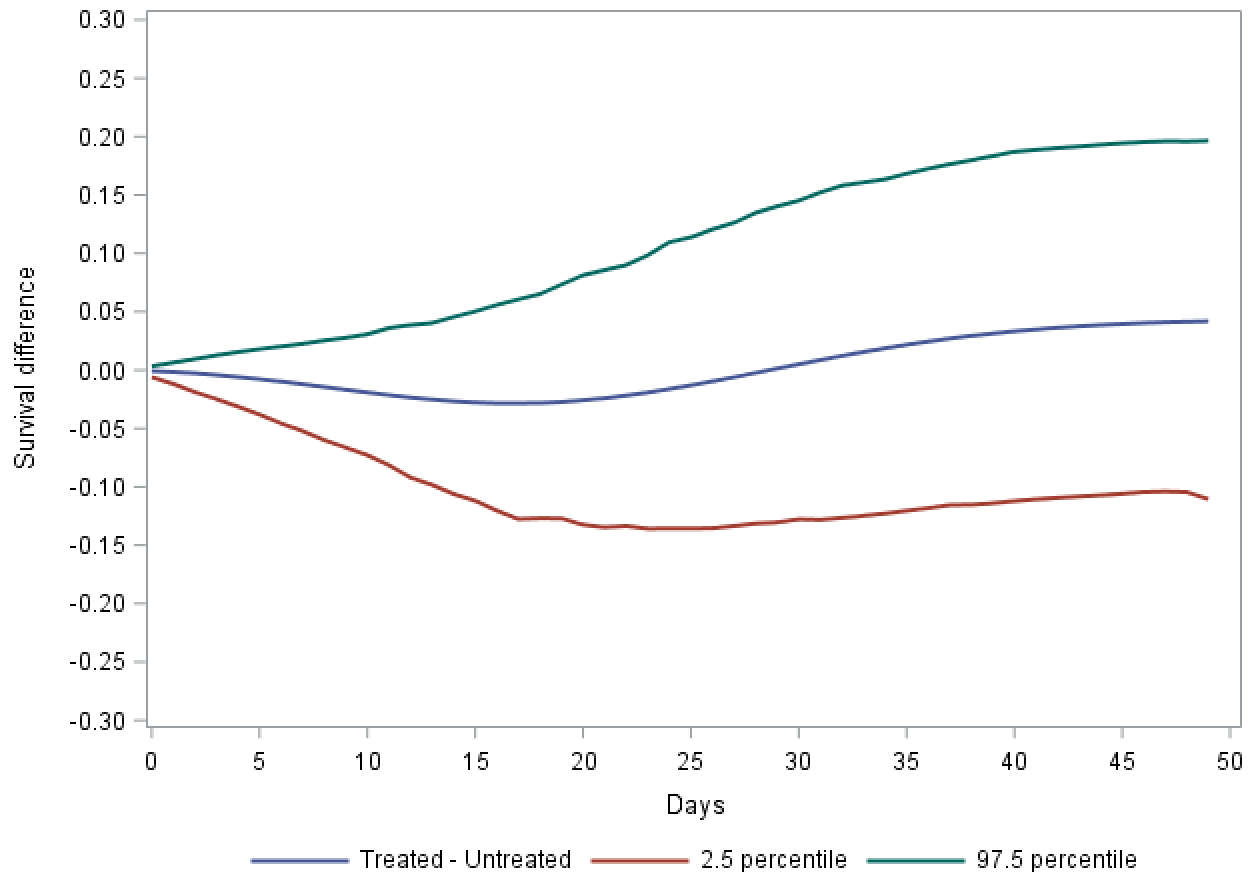 |
| AG.  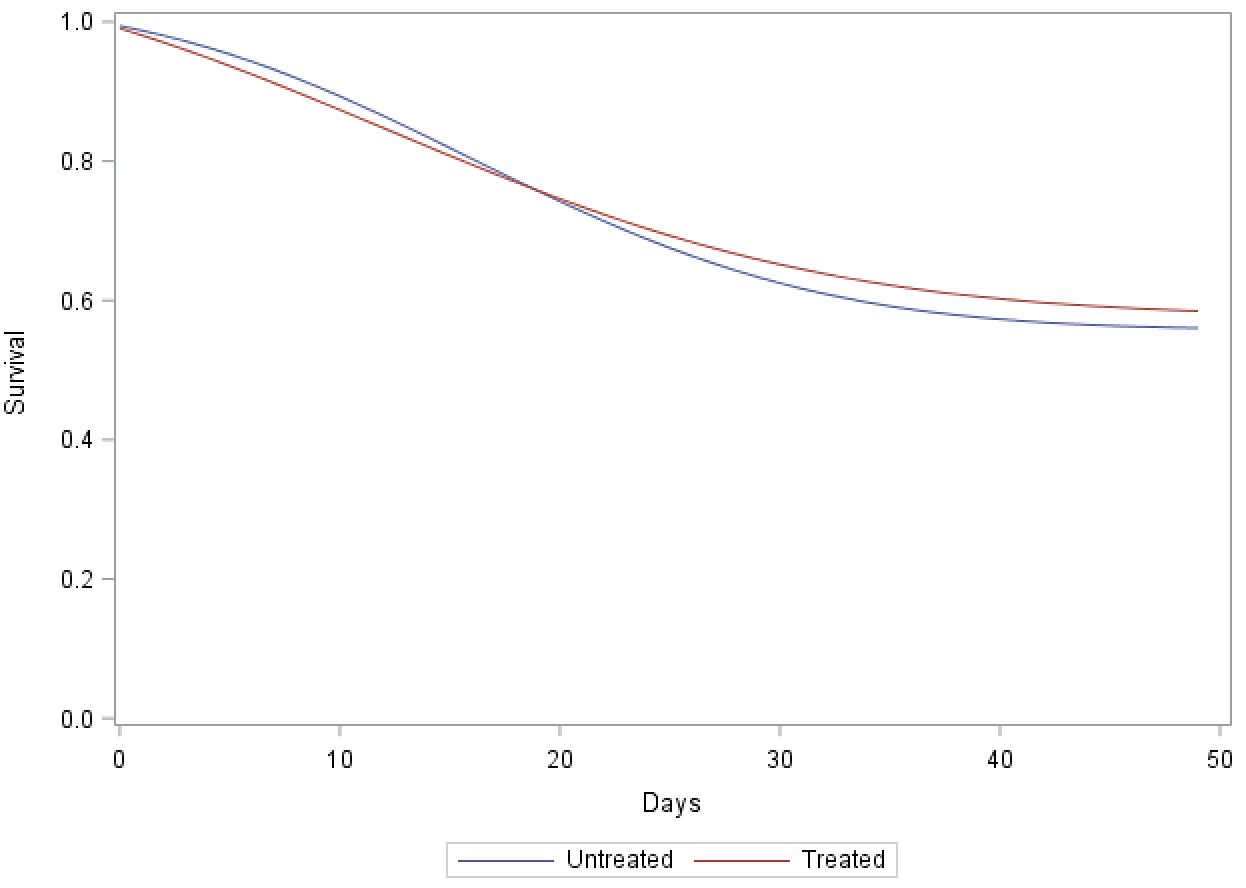 | AH.  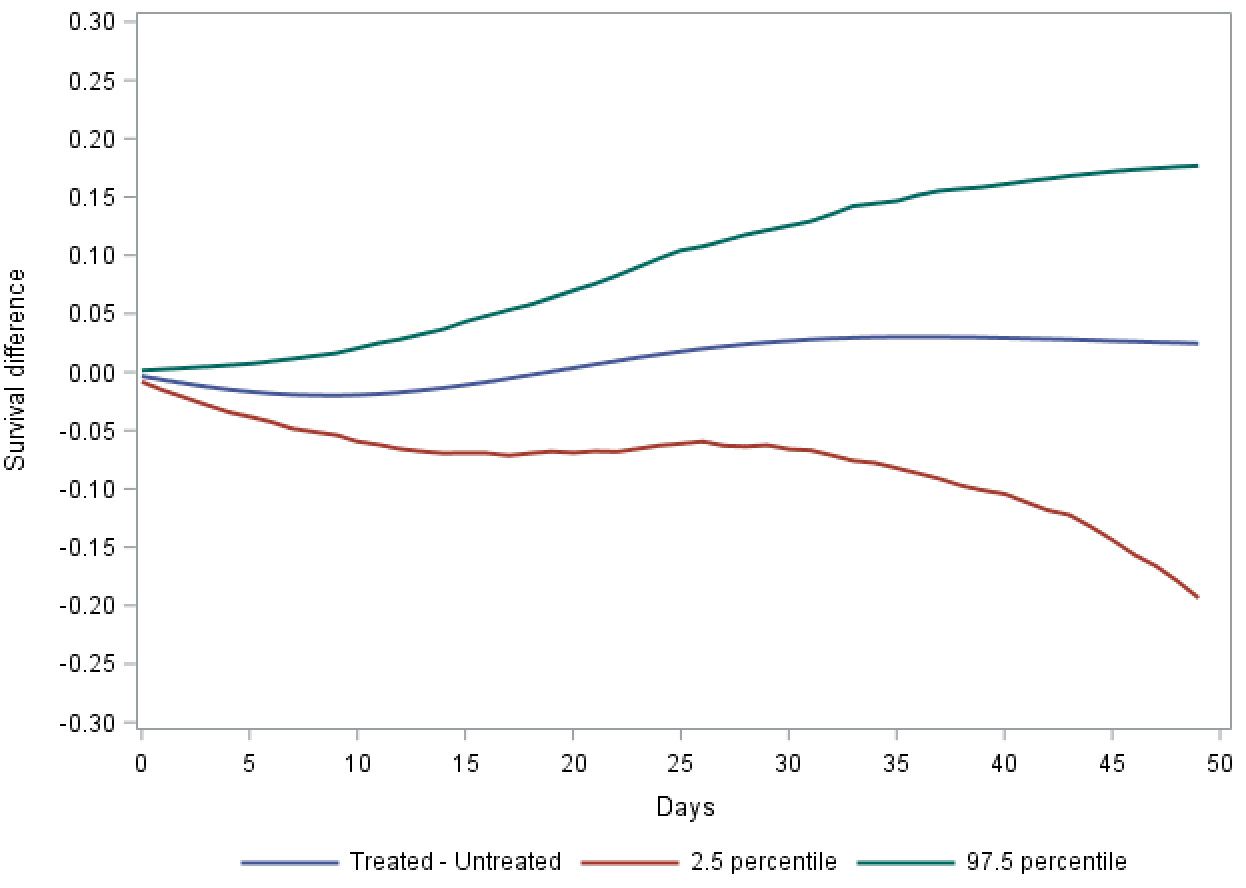 |
| AI.  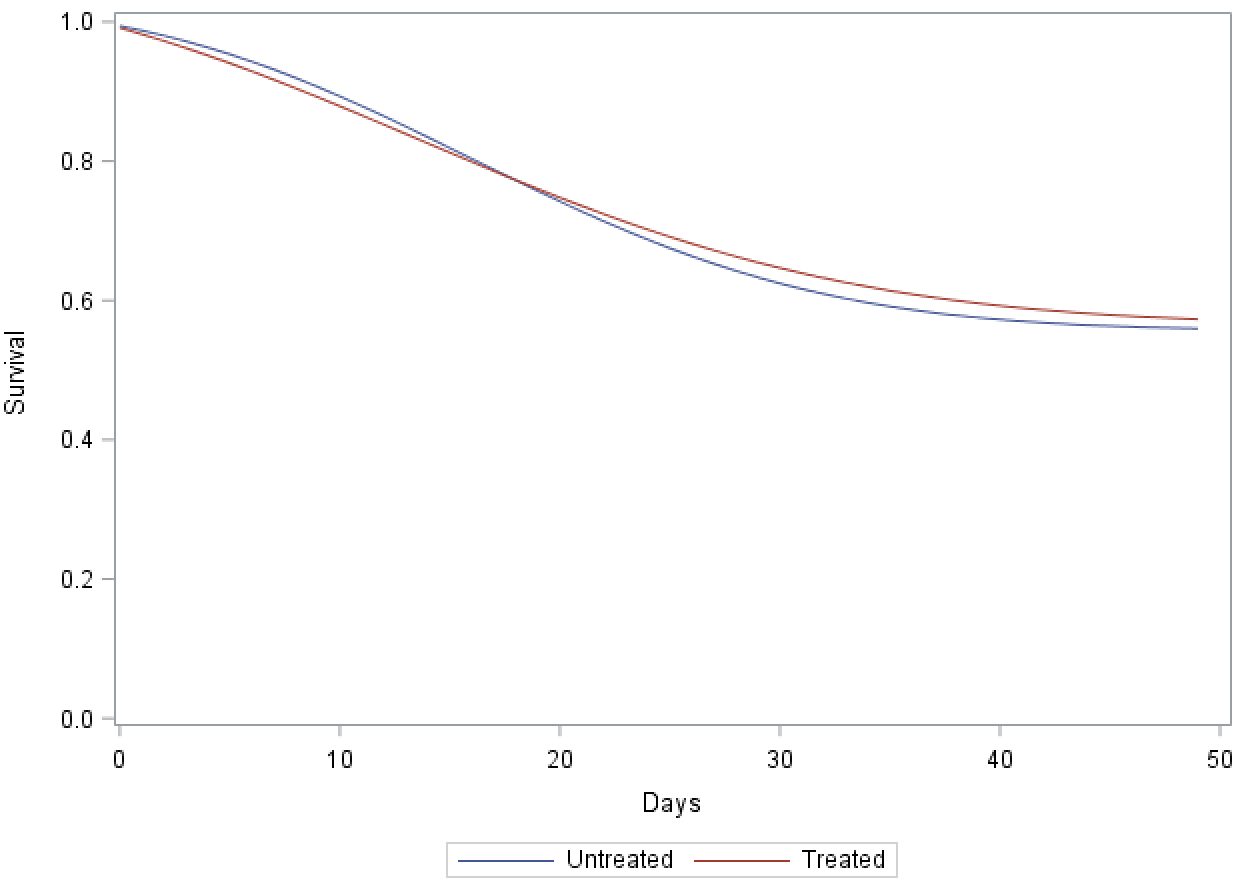 | AJ.  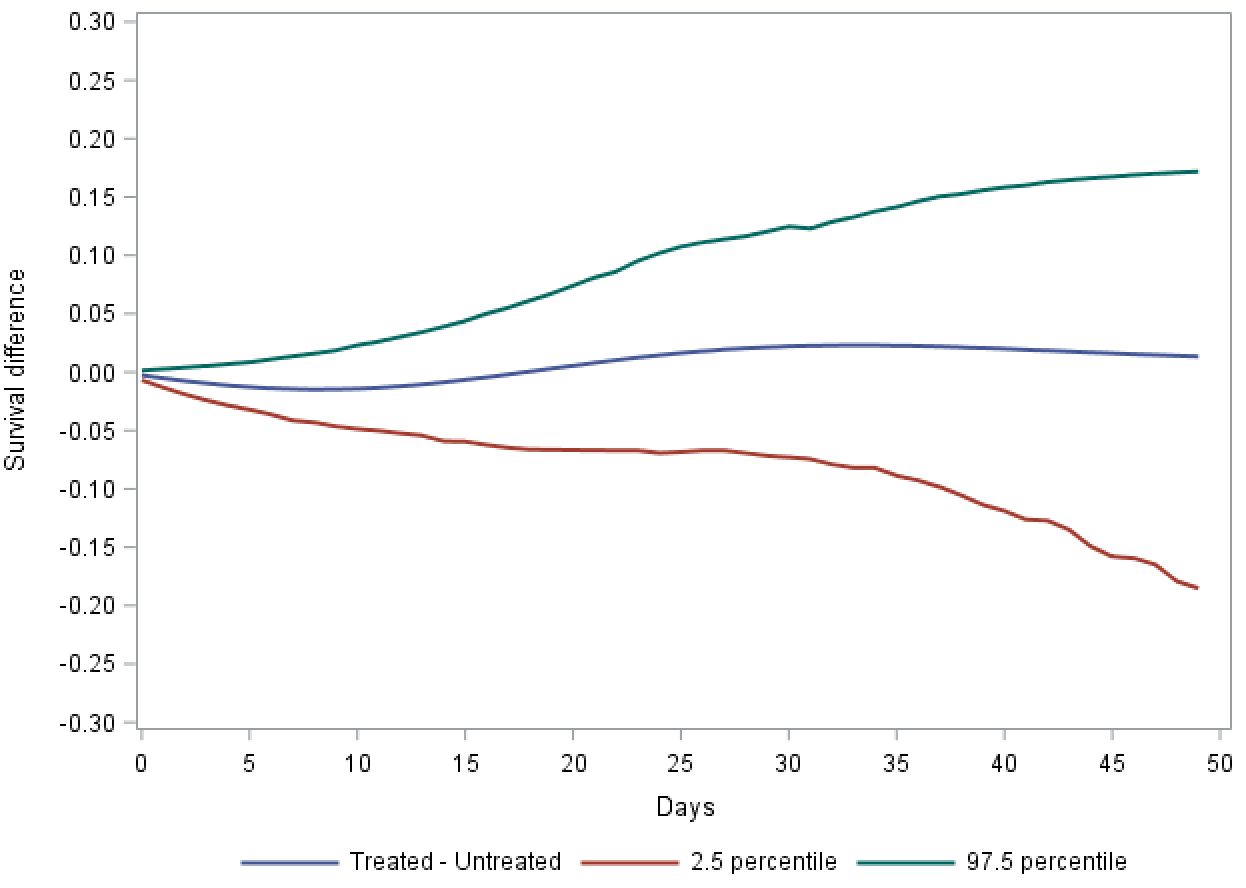 |

A. In-hospital Survival up to 50 days using 3-hour SOFA score assessment window;

B. In-hospital Survival up to 50 days using 12-hour SOFA score assessment window;

C. In-hospital Survival Difference using 3-hour SOFA score assessment window;

D. In-hospital Survival Difference using 12-hour SOFA score assessment window;

E. In-hospital Survival up to 50 days using 24-hour SOFA score assessment window;

F. In-hospital Survival up to 50 days using 1-day exposure window;

G. In-hospital Survival difference using 24-hour SOFA score assessment window;

H. In-hospital Survival difference using 1-day exposure window;

I. In-hospital Survival up to 50 days using 5-day SOFA score assessment window;

J. In-hospital Survival up to 50 days using Corticosteroid daily dose 200 to 400mg;

K. In-hospital Survival difference using 5-day SOFA score assessment window;

L. In-hospital Survival difference using Corticosteroid daily dose 200 to 400mg;

M. In-hospital Survival up to 50 days when individuals received no any corticosteroids as controls;

N. In-hospital Survival up to 50 days when excluding patients with asthma or COPD;

O. In-hospital Survival difference when individuals received no any corticosteroids as controls;

P. In-hospital Survival difference when excluding patients with asthma or COPD;

Q. In-hospital Survival up to 50 days when excluding hospitals that never prescribed corticosteroids;

R. In-hospital Survival up to 50 days when using g-formula;

S. In-hospital Survival difference when excluding hospitals that never prescribed corticosteroids;

T. In-hospital Survival difference when using g-formula;

U. In-hospital Survival up to 50 days using any dose of corticosteroids as controls;

V. In-hospital Survival up to 50 days when excluding >1 hospitalization due to sepsis or septic shock within 2014-2015;

W. In-hospital Survival difference using any dose of corticosteroids as controls;

X. In-hospital Survival difference when excluding >1 hospitalization due to sepsis or septic shock within 2014-2015;

Y. In-hospital Survival up to 90 days;

Z. In-hospital Survival up to 50 days when excluding patients without ventilation support;

AA. In-hospital Survival difference up to 90 days;

AB. In-hospital Survival difference when excluding patients without ventilation support;

AC. In-hospital Survival up to 50 days when excluding patients admitted for surgery;

AD. In-hospital Survival difference when excluding patients admitted for surgery;

AE. In-hospital Survival up to 50 days when using patients receiving hydrocortisone as treated;

AF. In-hospital Survival difference when using patients receiving hydrocortisone as treated;

AG. In-hospital Survival up to 50 days when all untested values assumed abnormal;

AH. In-hospital Survival difference when all untested values assumed abnormal;

AI. In-hospital Survival up to 50 days when excluding laboratory values with high percentage of untested patients from the analysis;

AJ. In-hospital Survival difference when excluding laboratory values with high percentage of untested patients from the analysis;
